# Sublethal disulfide stress facilitates TLR2-dependent NLRP3 inflammasome activation and pyroptosis in leukemia cells

**DOI:** 10.1038/s41419-026-09164-4

**Published:** 2026-08-01

**Authors:** Shoko Hosojima, Shenghui Zhi, Yamato Tanabe, Takeshi Kinoshita, Takayuki Hoshii, Takashi Suda, Kohsuke Tsuchiya

**Affiliations:** 1 Division of Immunology and Molecular Biology, Cancer Research Institute, Kanazawa University, Kakuma-machi, Kanazawa, Japan; 2 Division of Transcriptional Control and Engineering, Cancer Research Institute, Kanazawa University, Kakuma-machi, Kanazawa, Japan; 3 Molecular Genetics, Graduate School of Medical Sciences, Kanazawa University, Takara-machi, Kanazawa, Japan; 4 Division of Immune Environment Dynamics, Cancer Research Institute, Kanazawa University, Kakuma-machi, Kanazawa, Japan; 5 Immune Network Research Unit, Pursuit of Truth Research Core, Institute for Frontier Science Initiative, Kanazawa University, Kakuma-machi, Kanazawa, Japan; 6 Cancer–Immune System Interactions Unit, Cancer Research Institute, Kanazawa University, Kanazawa, Japan

**Keywords:** Cell death and immune response, NOD-like receptors

## Abstract

Inflammasomes are innate immune signaling platforms that activate caspase-1, which in turn cleaves gasdermin D (GSDMD) into its mature form, leading to pyroptosis, an inflammatory form of regulated necrosis. It has been reported that bacterial lipopeptides (BLPs) trigger pyroptosis through NLRP7 inflammasome activation. However, the underlying recognition mechanism remains entirely unclear, and the concept of BLP sensing by NLRP7 has not been well established. In this study, we sought to elucidate the mechanism by which BLPs induce inflammasome activation and pyroptosis in human acute myeloid leukemia cell lines. Through comprehensive analyses, we demonstrate that BLPs induce GSDMD-dependent pyroptosis not via the NLRP7 inflammasome, but through the NLRP3 inflammasome without the need for additional stimuli. Addition of KCl to the culture medium significantly suppressed BLP-induced pyroptosis, suggesting the involvement of K^+^ efflux in inflammasome activation. However, BLPs alone are generally thought to be insufficient to directly induce K^+^ efflux, and K^+^ channel inhibitors had minimal effects, prompting us to hypothesize that K^+^ efflux might result from sublethal membrane damage associated with a distinct cell death mechanism. Among several cell death inhibitors tested, reducing agents, inhibitors of disulfidptosis, which is necrotic cell death caused by excessive disulfide bonding in the actin cytoskeleton, markedly attenuated BLP-induced K^+^ efflux and pyroptosis. Consistent with this, BLP stimulation led to NADPH depletion and increased disulfide bond formation in β-actin, hallmarks of disulfidptosis. These findings suggest that disulfide stress contributes to NLRP3 inflammasome activation and pyroptosis triggered by BLPs in leukemia cells. To our knowledge, this is the first report linking disulfidptosis-associated disulfide stress to innate immune signaling, providing a conceptual framework for exploiting pyroptosis induction in leukemia cells.

## Introduction

The innate immune system serves as the first line of defense against pathogens and relies on pattern recognition receptors (PRRs) to detect pathogen- and damage-associated molecular patterns (PAMPs and DAMPs) [1–3]. PRRs include cell surface receptors such as Toll-like receptors (TLRs) and intracellular receptors such as NOD-like receptors (NLRs), which together enable broad pathogen sensing and initiate inflammatory responses through downstream signaling pathways. Several NLRs and absent in melanoma 2-like receptors (ALRs) form inflammasomes, multiprotein complexes that activate caspase-1 [2, 4]. Activated caspase-1 processes pro-IL-1β and pro-IL-18 into their mature forms and cleaves gasdermin D (GSDMD), inducing pyroptosis, a lytic form of regulated cell death [5–8]. GSDMD-mediated pore formation and subsequent plasma membrane rupture promote the release of inflammatory cytokines and DAMPs, thereby amplifying innate immune responses [9, 10]. Inflammasomes typically consist of an NLR or ALR, the adaptor ASC (apoptosis-associated speck-like protein containing a CARD), and pro-caspase-1 [2, 4].

Among inflammasome-forming NLRs, NLRP3 is the best-characterized member and requires two signals for full activation [2, 4]. Signal 1 (priming), typically provided by TLR or cytokine signaling, induces NLRP3 expression and licensing, whereas signal 2 (activation) is triggered by diverse stimuli, including microbial toxins, DAMPs, crystalline substances, and metabolic stress [2, 4, 11–14]. These stimuli converge on common cellular events such as K^+^ efflux, Ca^2+^ influx, reactive oxygen species (ROS) production, lysosomal damage, mitochondrial dysfunction, and organelle remodeling [2, 4, 15–19]. Among these upstream signals, K^+^ efflux remains a central and broadly conserved trigger for NLRP3 inflammasome assembly [16].

NLRP7 is an inflammasome-forming NLR that is highly expressed in reproductive tissues such as the testis, ovary, and early embryos [2]. In addition to inflammasome-related functions, NLRP7 has been implicated in maternal genomic imprinting and maintenance of genomic stability during human embryogenesis [20, 21]. NLRP7 inflammasome assembly has been reported to be induced by bacterial lipopeptides (BLPs), which are also known as TLR2 ligands [22, 23]. However, its activation mechanism remains largely unknown. It is unclear whether NLRP7 directly senses BLPs or is activated through indirect pathways. The deubiquitinase STAM-binding protein (STAMBP) has been suggested to enhance NLRP7 protein abundance and promote inflammasome formation in response to TLR ligands, but this does not sufficiently explain the activation phase [24]. However, BLPs have also been reported to activate the NLRP3 inflammasome in an NLR family CARD domain containing 5 (NLRC5)-dependent manner, and the relationship between this pathway and NLRP7 remains undefined [25, 26]. Furthermore, another study has suggested that overexpression of NLRP7 suppresses inflammasome activation by other NLRs [27]. At least three NLRP7 isoforms have been identified, and studies reporting pro- or anti-inflammasome functions of NLRP7 have employed different isoforms. Consequently, the field lacks a clear understanding of the activators and activation mechanisms of the NLRP7 inflammasome, and several key questions remain unresolved.

In this study, we initially aimed to determine whether NLRP7 senses BLPs and to elucidate its underlying molecular mechanism. We employed THP-1 cells, a human acute myeloid leukemia (AML)-derived monocytic cell line commonly used in previous NLRP7 inflammasome studies [22–24, 28, 29]. Our results showed that BLPs do not induce NLRP7 inflammasome activation. Instead, the results suggested that BLPs rapidly activate the NLRP3 inflammasome without requiring signal 2 stimuli. Furthermore, mechanistic analyses identified a potential role of disulfide stress in this process, representing as a novel aspect of inflammasome regulation.

## Results

### Bacterial lipopeptides induce inflammasome activation in near-confluent THP-1 cells

**Fig. 1:**
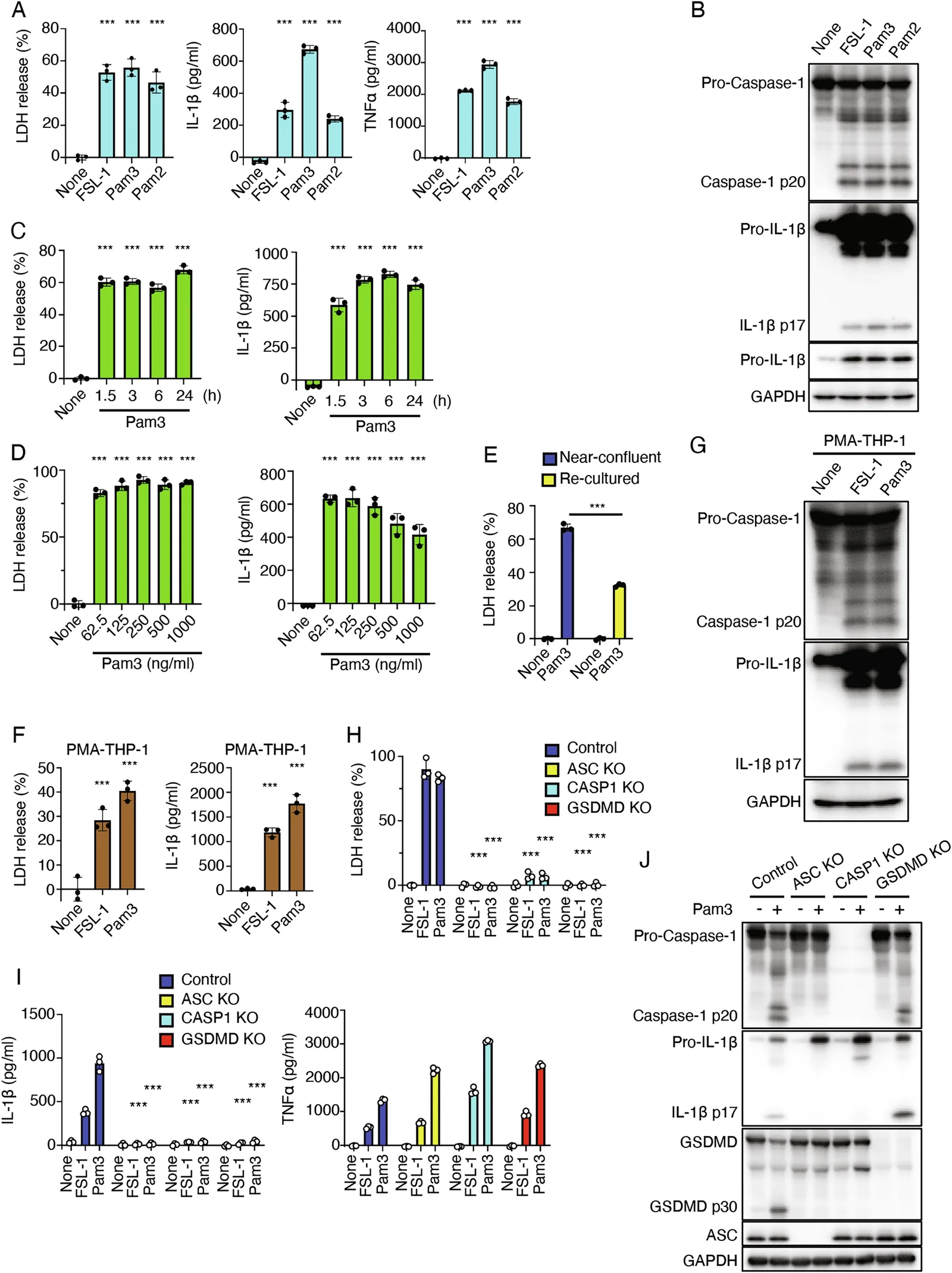
BLPs induce inflammasome activation and pyroptosis in near-confluent THP-1 cells. **A** THP-1 cells were stimulated with 500 ng/ml of FSL-1, Pam_3_CSK_4_ (Pam3), or Pam_2_CSK_4_ (Pam2) for 6 h. Cell lysis was monitored by LDH release assay (left), and levels of IL-1β (middle) and TNFα (right) in culture supernatants were assessed by ELISA. **B** Caspase-1 and IL-1β in culture supernatants plus cell lysates (S + L) were detected by immunoblotting. Release of LDH (left) and IL-1β (right) from THP-1 cells stimulated with Pam3 (500 ng/ml) for the indicated times (**C**) or with the indicated concentrations of Pam3 for 6 h (**D**). **E** Pam3-induced LDH release from near-confluent THP-1 cells or those re-cultured in fresh medium for 24 h before stimulation. **F**, **G** PMA-differentiated THP-1 cells were stimulated as in **A**. LDH release (**E**, left), IL-1β levels (**E**, right) in culture supernatants, and immunoblot analysis of caspase-1 and IL-1β (G) in S + L. **H–J** Control and gene-edited THP-1 cells (undifferentiated, near-confluent) were stimulated as in **A**. LDH release assay (**H**), secretion of IL-1β (I, left) and TNFα (**I** right), and immunoblot analysis of caspase-1, IL-1β, GSDMD, and ASC in S + L (**J**). The graphs depict the mean ± SD of triplicate cultures (**A**, **C**, **D**–**F**, **H**, **I**). Data are from one representative of three biologically independent experiments with similar results. *P* values were determined using one-way (**A**, **C**, **D**, **F**) or two-way (**H**, **I**) ANOVA followed by Dunnett’s multiple comparisons test or an unpaired *t* test with Welch’s correction (**E**). *P* values are shown only for comparisons of interest, including between the indicated groups or versus the respective control groups. *P < 0.05; **P < 0.01; ***P < 0.001; n.s., not significant.

We firstly examined whether bacterial lipopeptide (BLP) stimulation induces inflammasome activation in THP-1 cells. The synthetic BLP analogs FSL-1, Pam_3_CSK_4_, and Pam_2_CSK_4_ induced LDH release, an indicator of plasma membrane damage, and IL-1β secretion from THP-1 cells (Fig. 1A). Furthermore, these BLPs promoted caspase-1 activation and IL-1β maturation, suggesting inflammasome activation (Fig. 1B). Using Pam_3_CSK_4_, we then evaluated the time- and dose-dependent kinetics of inflammasome activation. Cell death and IL-1β release occurred as early as 1.5 h after stimulation, indicating that the response occurred rapidly (Fig. 1C). Responses were observed at concentrations ranging from 1 µg/ml to 62.5 ng/ml (Fig. 1D). During repeated experiments, we noticed that the culture conditions of THP-1 cells had a major impact on their responsiveness to BLPs. When THP-1 cells were cultured to near confluency before stimulation, Pam_3_CSK_4_ strongly induced LDH release (Fig. 1E). In contrast, when these near-confluent cells were re-cultured in fresh medium for 24 h before the experiment, LDH release was markedly reduced. These observations suggest that the metabolic state of THP-1 cells influences their inflammasome responses to BLPs. Therefore, unless otherwise stated, we used THP-1 cells that had been grown to near confluency for subsequent experiments. PMA-differentiated THP-1 cells also exhibited LDH release, IL-1β secretion, caspase-1 activation, and IL-1β maturation upon BLP stimulation (Fig. 1F, G). To determine whether GSDMD and inflammasome components contribute to BLP-induced cell death, we generated THP-1 cell lines deficient in ASC, caspase-1, or GSDMD. Loss of these molecules did not affect TNF-α production but almost completely abolished LDH release and IL-1β secretion (Fig. 1H, I). In addition, caspase-1 was not activated in ASC-deficient cells, and ASC- or caspase-1-deficient cells did not produce the N-terminal fragment of GSDMD after stimulation with Pam_3_CSK_4_ (Fig. 1J). These results demonstrate that BLPs induce inflammasome activation and trigger pyroptosis in THP-1 cells.

### NLRP7 does not enhance pyroptosis triggered by bacterial lipopeptides

**Fig. 2:**
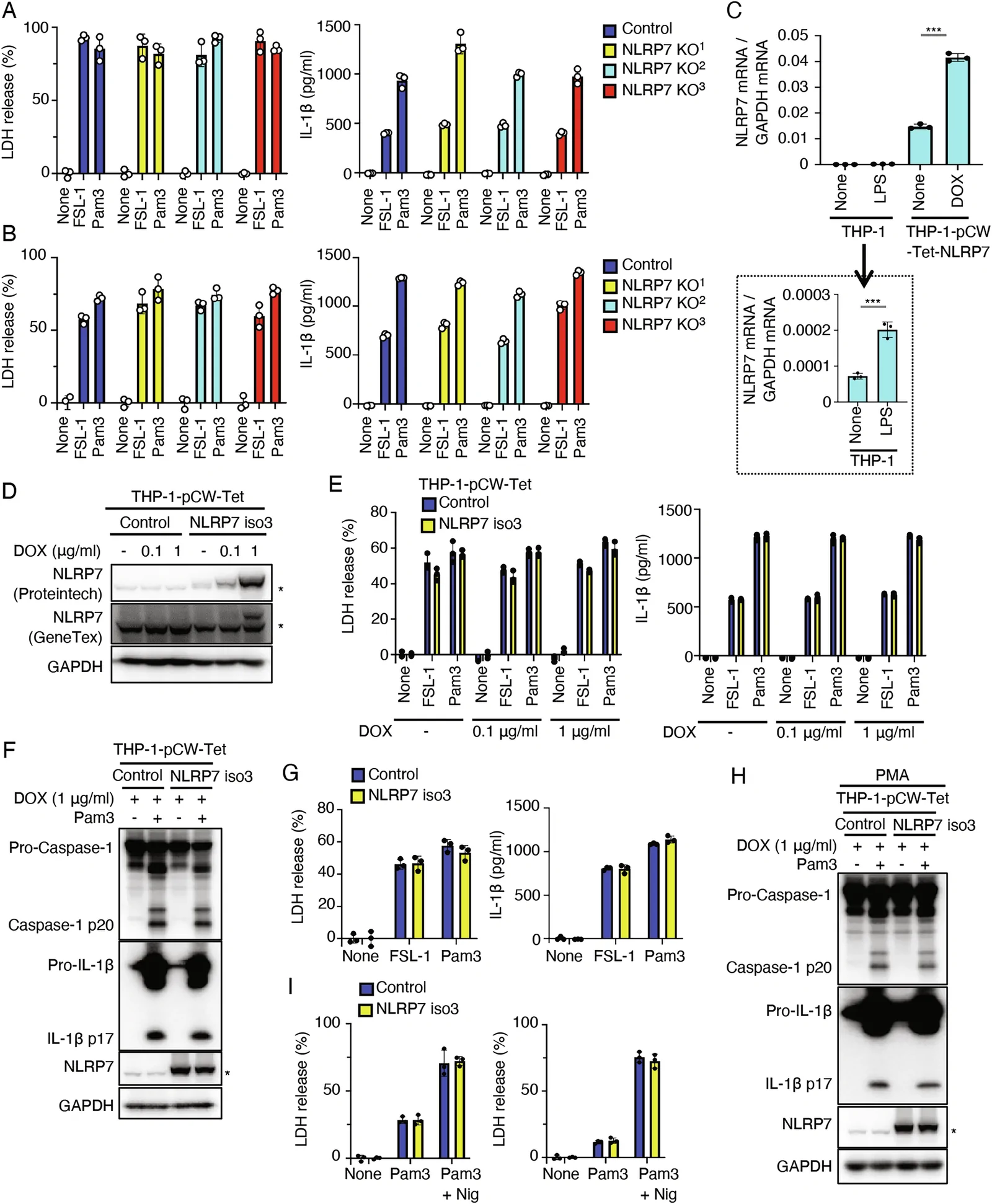
Expression of NLRP7 does not enhance BLP-induced inflammasome activation. Control and NLRP7 KO THP-1 cells were left undifferentiated (**A**) or differentiated with PMA (**B**) and then stimulated with FSL-1 or Pam3 (500 ng/ml) for 5 h. LDH release (left) and IL-1β levels in culture supernatants (right) were measured. **C** Quantitative RT-PCR analysis of NLRP7 mRNA expression in WT THP-1 cells stimulated with or without LPS (100 ng/ml) for 24 h or in THP-1 cells transduced with DOX-inducible NLRP7 isoform 2 and treated with or without DOX (100 ng/ml) for 24 h. An inset graph outlined by a dotted box shows a magnified view of the WT THP-1 cell data. Control (DOX-inducible BFP) and DOX-inducible NLRP7 isoform 3-transduced THP-1 cells were treated with DOX for 24 h (**D**) and then stimulated as in **A** (**E**, **F**). Immunoblot analysis of NLRP7 (**D**, **F**), caspase-1, and IL-1β (**F**) in cell lysates (**E**) or S + L (F). The asterisks (*) indicate nonspecific bands. LDH release (left) and IL-1β levels in culture supernatants (right) were measured (**E**). **G**, **H** PMA-differentiated THP-1 cells harboring DOX-inducible NLRP7 isoform 3 or control were treated with DOX (1 μg/ml) for 24 h and then stimulated as in **A**. LDH release assay (**G**, left), IL-1β levels (**G**, right) in culture supernatants, and immunoblot analysis (**H**) in S + L. **I** Undifferentiated (left) and PMA-differentiated (right) THP-1 cells harboring DOX-inducible NLRP7 isoform 3 or control were re-cultured in fresh medium in the presence of DOX (1 μg/ml) for 24 h. The cells were then stimulated with or without Pam3 (500 ng/ml) for 3 h and additionally stimulated with or without nigericin (10 μM) for 2 h. LDH release was measured. The graphs depict the mean ± SD of triplicate cultures (**A**–**C**, **E**, **G**, **I**). Data are from one representative of three biologically independent experiments with similar results. *P* values were determined using two-way ANOVA followed by Tukey’s multiple comparisons test (**C**). *P* values are shown only for comparisons of interest, including between the indicated groups or versus the respective control groups. *P < 0.05; **P < 0.01; ***P < 0.001; n.s., not significant.

To test whether NLRP7 contributes to BLP-induced inflammasome activation, we generated NLRP7-knockout (KO) THP-1 cell lines (Supplementary Fig. 1A). However, knockout of NLRP7 did not affect BLP-induced LDH release or IL-1β secretion, regardless of whether the cells were differentiated (Fig. 2A, B). In THP-1 cells, the expression levels of NLRP7 mRNA were low under both unstimulated and LPS-stimulated conditions, while NLRP3 was expressed at high levels (Fig. 2C, Supplementary Fig. 1B). Endogenous NLRP7 protein was also undetectable by Western blotting (Supplementary Fig. 1C). The results are consistent with a large-scale transcriptional analysis of human cell lines showing that NLRP7 mRNA is not detectable in THP-1 cells [30]. We next generated THP-1 cell lines in which either isoform 3 of NLRP7 (the longest isoform) or isoform 2 (a shorter isoform) could be expressed in a doxycycline (DOX)-dependent manner. In these cell lines, DOX treatment increased NLRP7 mRNA levels and allowed detection of each isoform at the protein level (Fig. 2C, D, and Supplementary Fig. 2A). However, forced expression of NLRP7 did not significantly enhance BLP-induced cell death, IL-1β secretion, or caspase-1 activation (Fig. 2E, F, and Supplementary Fig. 2B-2D). Similar results were obtained in PMA-differentiated THP-1 cells (Fig. 2G, H). Even when THP-1 cell lines were cultured in fresh medium for 24 h prior to stimulation, BLP-induced cell death was not increased by overexpression of NLRP7 (Fig. 2I). Moreover, activation of the NLRP3 inflammasome by nigericin stimulation was not affected by NLRP7 overexpression. Taken together, these results suggest that NLRP7 does not contribute to BLP-induced inflammasome activation in THP-1 cells.

### Essential role of NLRP3 in pyroptosis induction by bacterial lipopeptides

**Fig. 3:**
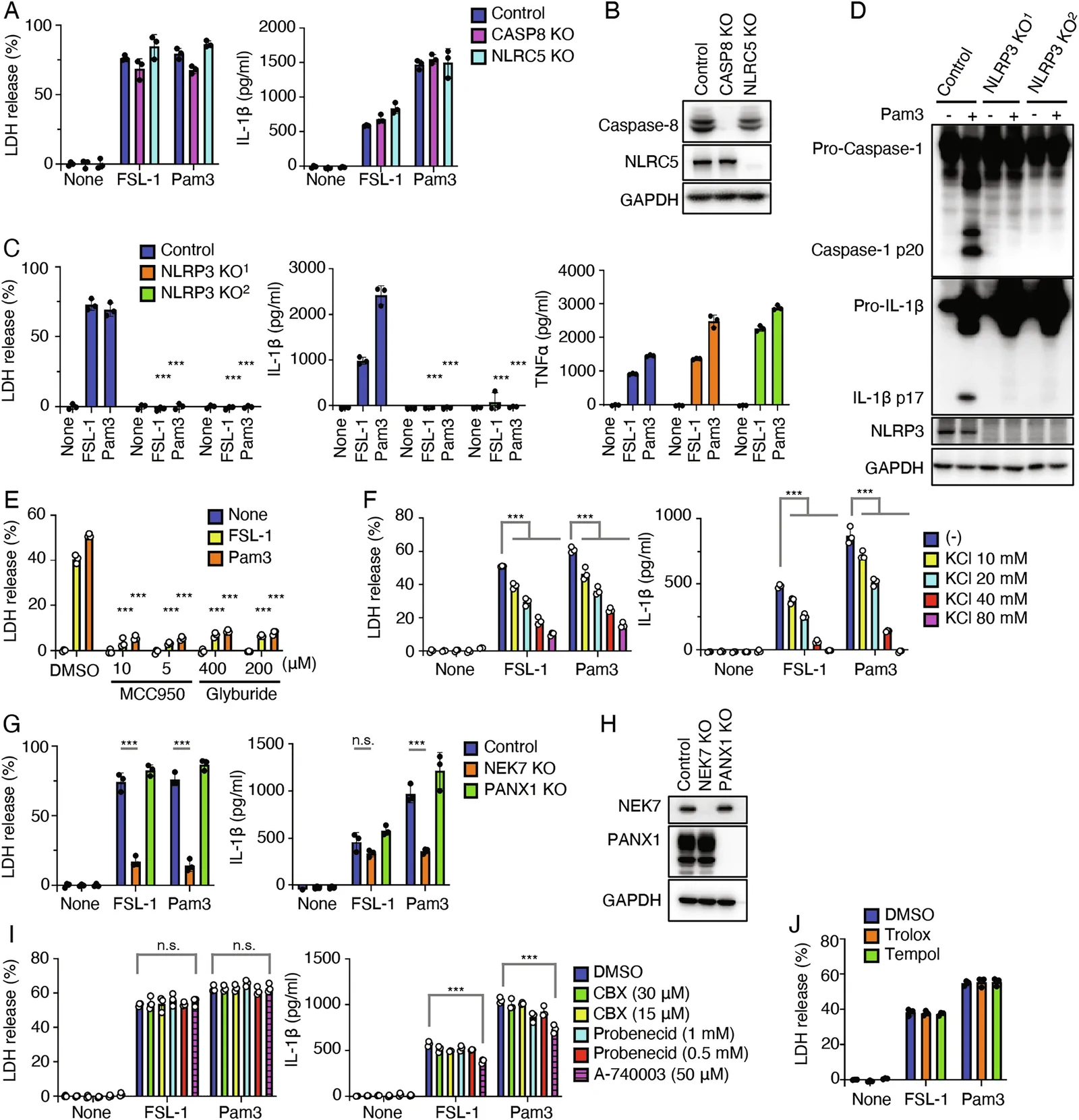
NLRP3 mediated BLP-induced inflammasome activation. **A–D**, **G**, **H** Control and gene-edited THP-1 cells (undifferentiated, near-confluent) were stimulated with FSL-1 or Pam3 (500 ng/ml) for 6 h. LDH release assay (left panels in **A**, **C**, **G**), IL-1β levels (right panels in **A**, **G** and middle panel in **C**), TNFα levels (**C**, right) in culture supernatants, and immunoblot analysis of the indicated proteins in cell lysates (**B**, **H**) or in S + L (**D**). **E**, **F**, **I**, **J** WT THP-1 cells were pretreated with NLRP3 inhibitors (MCC950, glyburide in **E**), pannexin-1 inhibitors (CBX, probenecid in **I**), the P2X7 inhibitor A-740003 (**I**), or ROS inhibitors (100 μM of trolox or tempol in **J**) for 30 min. KCl was added to the cultures just before stimulation. The cells were then stimulated as in **A**. LDH release (**E**, **J**, and left panels in **F**, **I**) and IL-1β levels (right panels in **F**, **I**) in culture supernatants were measured. The graphs depict the mean ± SD of triplicate cultures (**A**, **C**, **E**–**G**, **I**, **J**). Data are from one representative of three biologically independent experiments with similar results. *P* values were determined using two-way ANOVA followed by Dunnett’s multiple comparisons test (**C**, **E**–**G**, **I**). *P* values are shown only for comparisons of interest, including between the indicated groups or versus the respective control groups. *P < 0.05; **P < 0.01; ***P < 0.001; n.s., not significant.

NLRC5 has been reported to participate in inflammasome activation in response to PAMPs such as LPS, Pam_3_CSK_4_ and FSL-1 in THP-1 cells [25]. More recently, a PANoptosome complex composed of NLRC5, NLRP12, ASC, NLRP3, and caspase-8 has been shown to mediate cell death induced by stimuli including heme and various PAMPs [31]. We therefore examined whether caspase-8, NLRC5, or NLRP3 contributes to BLP-induced pyroptosis. In THP-1 cells deficient in caspase-8 or NLRC5, neither cell death nor IL-1β secretion induced by FSL-1 or Pam_3_CSK_4_ was affected (Fig. 3A, B). In contrast, NLRP3 KO cells failed to undergo LDH release, IL-1β secretion, caspase-1 activation, and IL-1β maturation (Fig. 3C, D). NLRP3-deficient THP-1 cells did not release LDH even after 24 h of BLP stimulation (Supplementary Fig. 3A). Furthermore, the NLRP3 inhibitors MCC950 and glyburide markedly reduced both cell death induction and IL-1β secretion (Fig. 3E). Forced expression of NLRP7 did not increase BLP-induced pyroptosis and IL-1β secretion even in the presence of MCC950 (Supplementary Fig. 3B, C). These results strongly suggest that BLPs activate the NLRP3 inflammasome independently of NLRP7, NLRC5, or the PANoptosome. K^+^ efflux is a critical event in most NLRP3 activation models. When various concentrations of KCl were added to the culture medium to block K^+^ efflux, BLP-induced cell death and IL-1β secretion decreased in a KCl dose-dependent manner (Fig. 3F). In addition, BLP stimulation of THP-1 cells lacking NEK7, which acts to support NLRP3 activation, resulted in substantially reduced, but not completely abolished, cell death and IL-1β secretion compared with control cells (Fig. 3G, H) [2, 32]. Both NEK7-dependent and NEK7-independent (yet K^+^ efflux–dependent) pathways have been described for human NLRP3 inflammasome activation, and our results are consistent with the previous study [13]. A previous study proposed a model in which TLR2 agonists induce IL-1β secretion from THP-1 cells through activation of the purinagic receptor P_2_X_7_ by ATP released via pannexin-1 [33]. However, knockout of pannexin-1 had no effect on BLP-induced cell death and IL-1β secretion (Fig. 3G, H), and pannexin-1 inhibitors (carbenoxolone, probenecid) likewise had no effect (Fig. 3I). Moreover, the P_2_X_7_ antagonist A-740003 did not inhibit LDH release and only slightly reduced IL-1β secretion (Fig. 3I), suggesting that the previously proposed model does not substantially account for BLP-induced NLRP3 inflammasome activation. Additionally, the ROS scavengers Trolox and Tempol did not affect responses to BLPs (Fig. 3J). Taken together, these results demonstrate that BLPs induce NLRP3 activation in a K^+^ efflux-dependent manner.

### Induction of pyroptosis by bacterial lipopeptides involves the TLR2-IKKβ signaling pathway

**Fig. 4:**
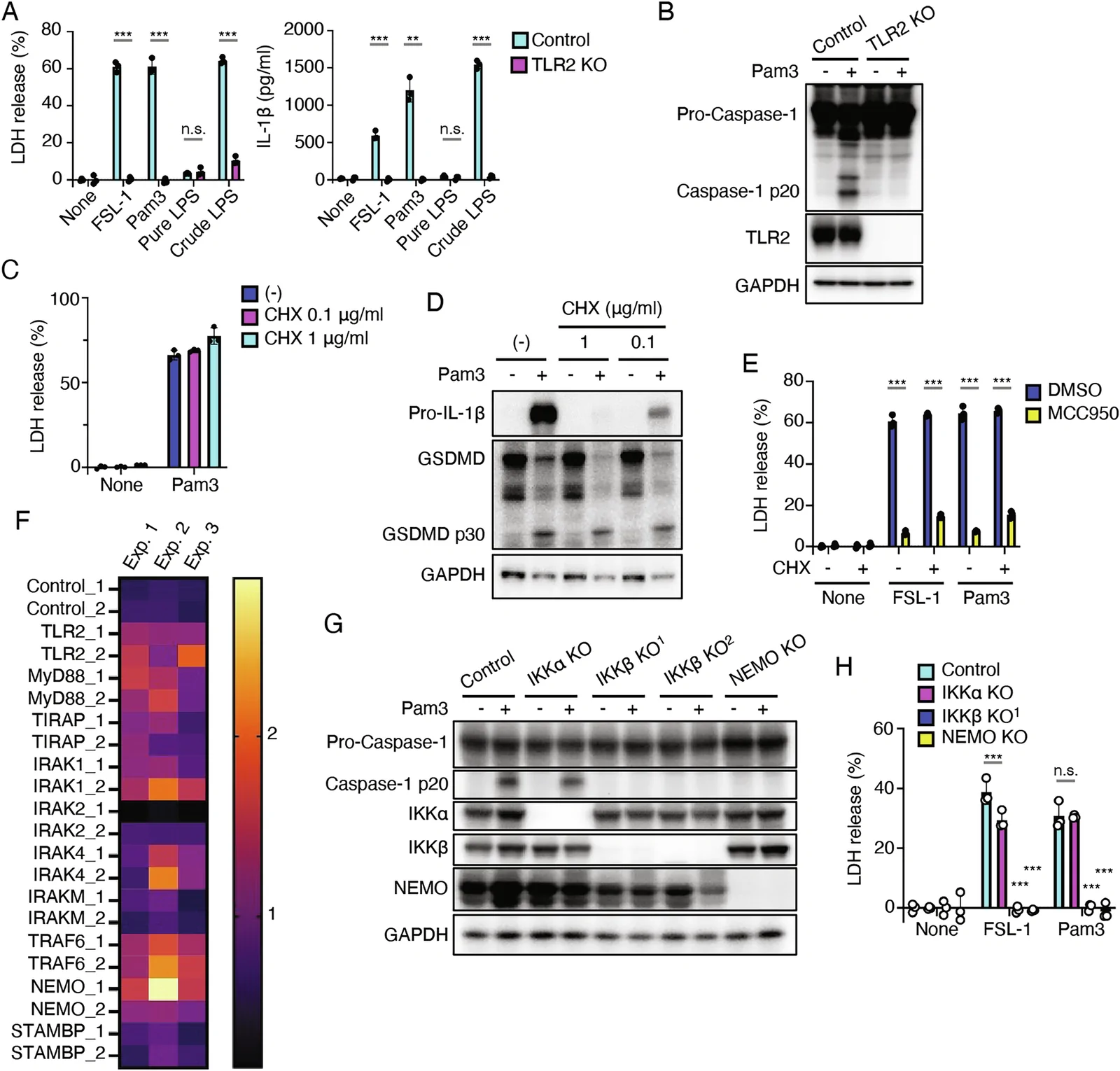
BLP-induced inflammasome activation involves TLR2 signaling molecules, but not de novo protein synthesis. **A**, **B**, **G**, **H** Control and gene-edited THP-1 cells (undifferentiated, near-confluent) were stimulated with FSL-1, Pam3 (500 ng/ml), ultrapure LPS, or crude LPS (100 ng/ml) for 6 h. LDH release (**H** and left panel in **A**) and IL-1β levels in culture supernatants (**A**, right) were measured. Immunoblot analysis of caspase-1, TLR2, and IKKs in S + L (**B**, **G**). **C–E** WT THP-1 cells were pretreated with cycloheximide (CHX) along with or without MCC950 for 30 min and then stimulated as in **A**. LDH release assay (**C**, **E**) and immunoblot analysis of IL-1β in S + L (**D**). **F** A gene-edited THP-1 cell population was generated by introducing a panel of gRNAs targeting TLR signaling components into Cas9-expressing THP-1 cells. The cells were stimulated with Pam3 (500 ng/ml) for 6 h, and surviving cells were isolated. Changes in the relative abundance of each gRNA before and after stimulation were quantified, and the results are presented as fold changes (post-/pre-stimulation) in the heat map. The graphs depict the mean ± SD of triplicate cultures (**A**, **C**, **E**, **H**). Data are from one representative of three biologically independent experiments with similar results (**A**–**E**, **G**, **H**) or pooled from three independent experiments (**F**). *P* values were determined using two-way ANOVA followed by Dunnett’s multiple comparisons test (**H**) or an unpaired *t* test with Welch’s correction (**A**, **E**). *P* values are shown only for comparisons of interest, including between the indicated groups or versus the respective control groups. *P < 0.05; **P < 0.01; ***P < 0.001; n.s., not significant.

To investigate whether TLR2, a recognition receptor for BLPs, contributes to inflammasome activation [1]. Knockout of TLR2 abolished BLP-induced cell death and secretion of IL-1β (Fig. 4A). In addition, Pam_3_CSK_4_-induced caspase-1 activation was totally suppressed in TLR2 KO cells (Fig. 4B). On the other hand, inhibition of de novo protein synthesis by cycloheximide did not block BLP-induced, NLRP3-dependent cell death (Fig. 4C–E). These results indicate that TLR2 contributes to NLRP3 inflammasome activation independently of de novo protein synthesis. We therefore sought to identify signaling molecules downstream of TLR2 that are involved in inflammasome activation [1]. To this end, we lentivirally introduced a panel of gRNAs targeting TLR signaling molecules into Cas9-expressing THP-1 cells to generate an gene-edited cell population. After stimulating the cell population with Pam_3_CSK_4_, surviving cells were collected by cell sorting, and changes in the abundance of each gRNA before and after stimulation were analyzed. Cells transduced with gRNAs targeting *MYD88*, *TIRAP*, *IRAK1*, *IRAK4*, *TRAF6*, and *IKBKG* were relatively enriched after Pam_3_CSK_4_ stimulation, and this suggested that the signaling pathway from MyD88 to the IκB kinase (IKK) complex is required for inflammasome activation (Fig. 4F, and Supplementary Fig. 4). NF-κB essential modulator (NEMO), encoded by *IKBKG*, forms a complex with IKKα and IKKβ and plays a crucial role in their activation [1]. We therefore generated THP-1 cell lines lacking NEMO, IKKα, or IKKβ and stimulated them with BLPs (Fig. 4G). Loss of NEMO or IKKβ completely abolished caspase-1 activation and LDH release, whereas loss of IKKα had little effect (Fig. 4G, 4H). Recent studies have shown that IKKβ interacts with NLRP3 to facilitate its recruitment to the trans-Golgi and plays a critical role for NLRP3 priming in a de novo synthesis-independent manner [12, 13, 26]. Our results are in line with these previous reports.

### Necroptosis and ferroptosis are not involved in pyroptosis induction by bacterial lipopeptides

**Fig. 5:**
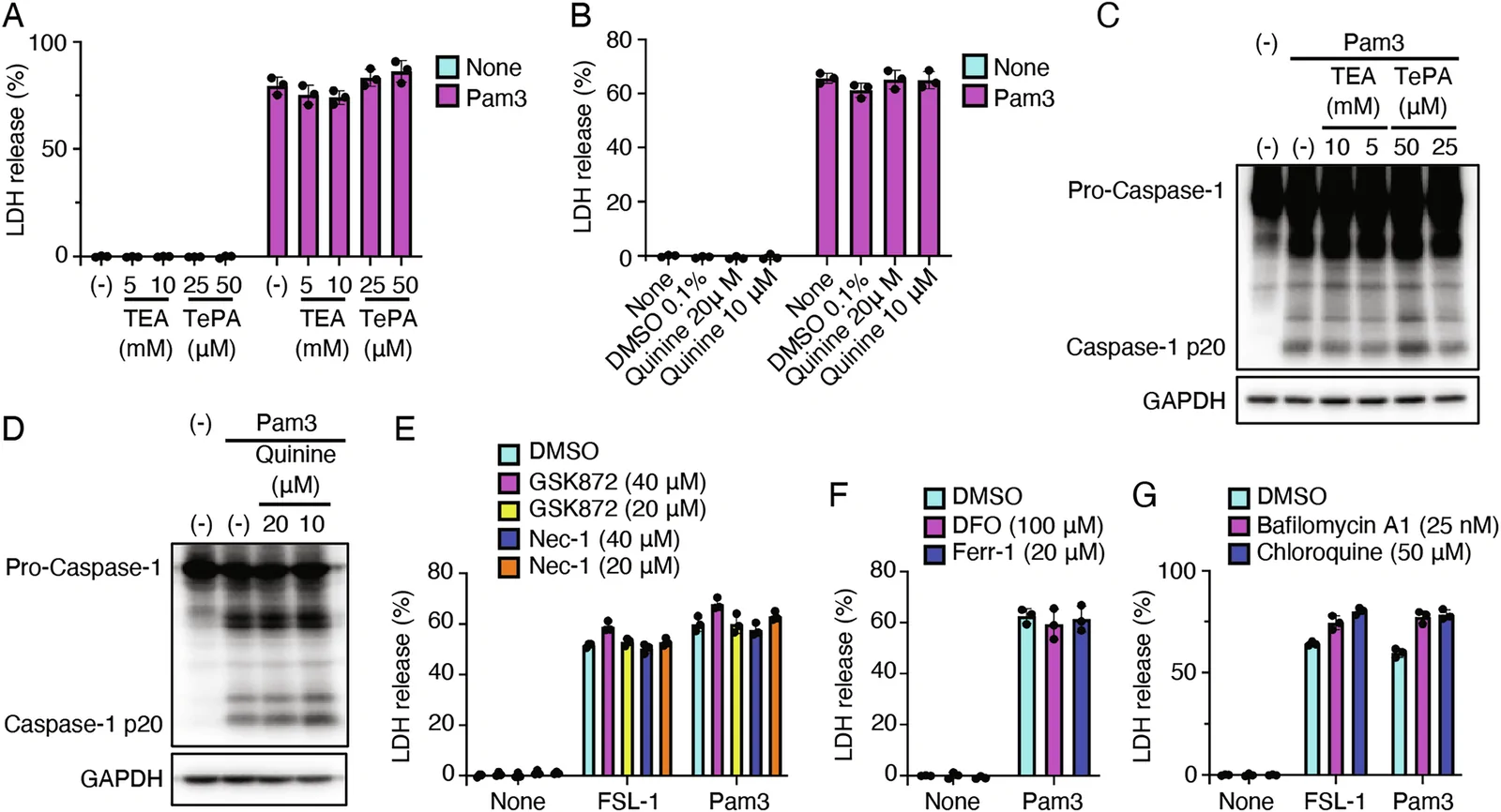
No significant effect of inhibitors for K^+^ channels, necroptosis, ferroptosis, and lysosomes on BLP-induced pyroptosis. **A–G** WT THP-1 cells (undifferentiated, near-confluent) were pretreated with K^+^ channel inhibitors (**A**–**D**), necroptosis inhibitors (**E**), ferroptosis inhibitors (**F**), or lysosome inhibitors (**G**) and then stimulated with FSL-1 or Pam3 (500 ng/ml) for 3 h (**A**–**D**) or 6 h (**E**–**G**). LDH release was measured (**A**, **B**, **E**–**G**). Immunoblot analysis of caspase-1 in S + L (**C**, **D**). The graphs depict the mean ± SD of triplicate cultures. Data are from one representative of three biologically independent experiments with similar results.

**Fig. 6:**
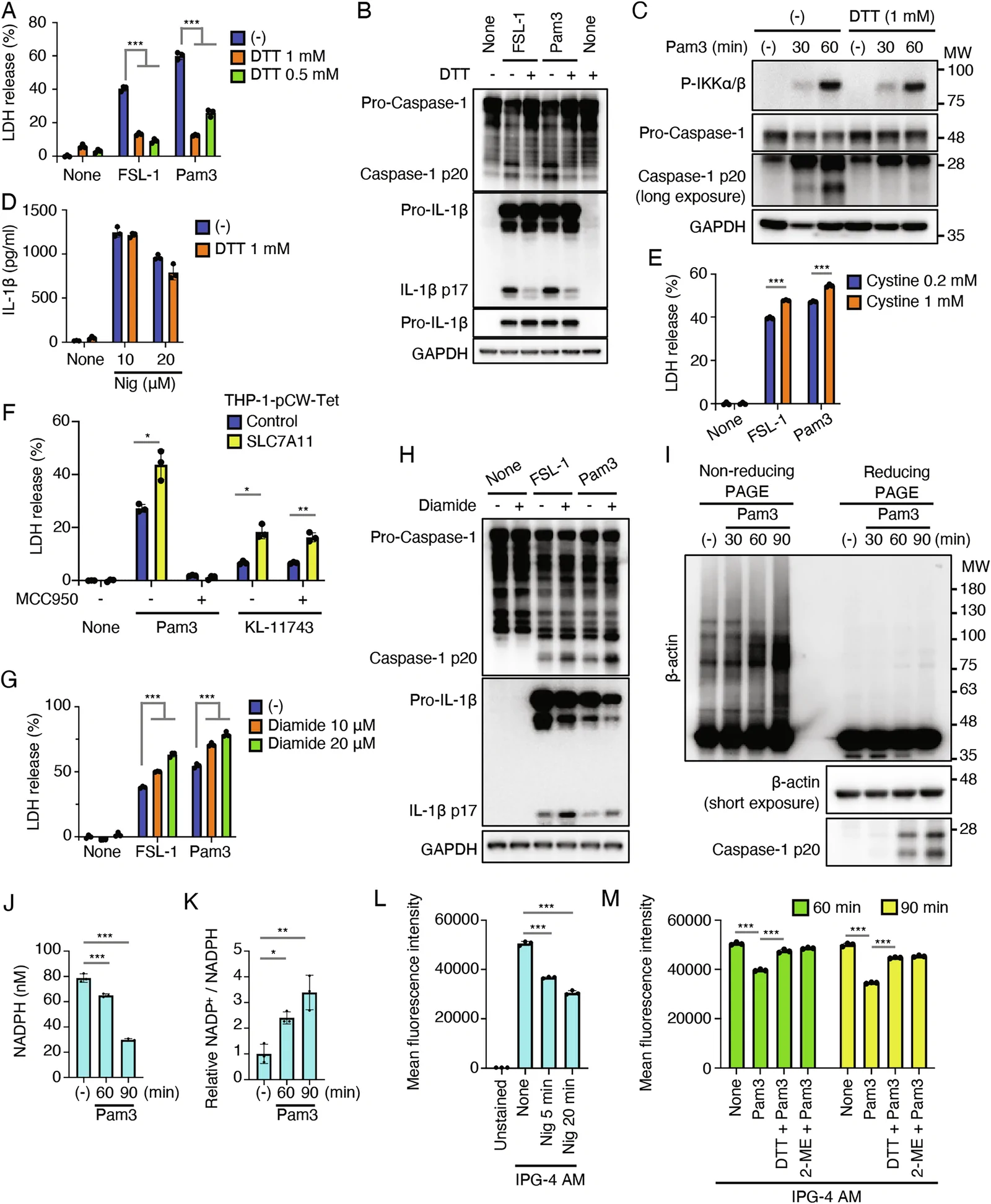
Disulfide stress facilitates BLP-induced NLRP3 inflammasome activation. **A** WT THP-1 cells were stimulated with FSL-1 or Pam3 (500 ng/ml) in the presence or absence of DTT for 6 h. LDH release was measured. **B**, **C** GSDMD KO THP-1 cells were stimulated with FSL-1 or Pam3 (500 ng/ml) in the presence or absence of DTT for 30, 60 (D), or 90 (C) min. Immunoblot analysis of caspase-1, IL-1β, and phospho-IKKα/β in cell lysates. **D** PMA-differentiated THP-1 cells were stimulated with nigericin for 1.5 h, and IL-1β levels in the culture supernatants were measured. **E** WT THP-1 cells incubated in high (1 mM) or normal (0.2 mM) concentration of cystine for 24 h were stimulated as in A. LDH release assay. **F** Control and SLC7A11-overexpressing THP-1 cells were pretreated with or without MCC950 (10 μM) for 30 min and then stimulated as in A or treated with the GLUT1-4 inhibitor KL-11743 (10 μM) for 5.5 h. LDH release assay. WT (**G**) or GSDMD KO (**H**) THP-1 cells were stimulated as in A for 6 h (**G**) or 90 min (**H**). Diamide was added to the cultures 30 min after stimulation. LDH release assay (**G**). Immunoblot analysis (**H**). **I–K** GSDMD KO THP-1 cells were stimulated with Pam3 for 30, 60, and 90 min. Reducing and non-reducing SDS-PAGE followed by detection of β-actin and caspase-1 by immunoblotting (**I**). Cellular NADPH and NAPD^+^ levels were measured (**J**, **K**). The results are presented as relative NAPD^+^/NADPH ratio (**K**). **L**, **M** IPG-4 AM-labeled GSDMD KO THP-1 cells were stimulated with 10 μM nigericin (**L**) or Pam3 (**M**) in the presence or absence of DTT (0.5 mM) or 2-ME (2 mM) for the indicated times. Intracellular K^+^ levels were analyzed by flow cytometry based on IPG-4 fluorescence, and the corresponding mean fluorescence intensity is shown (**L**, **M**). The graphs depict the mean ± SD of triplicate cultures (**A**, **D**, **E**–**G**, **J**–**M**). Data are from one representative of three biologically independent experiments with similar results. *P* values were determined using one-way (**J**–**L**) or two-way (**A**, **G**) ANOVA followed by Dunnett’s multiple comparisons test or two-way ANOVA followed by Tukey’s multiple comparisons test (**M**) or an unpaired t test with Welch’s correction (**E**, **F**). *P* values are shown only for comparisons of interest, including between the indicated groups or versus the respective control groups. *P < 0.05; **P < 0.01; ***P < 0.001; n.s., not significant.

In general, activation of the NLRP3 inflammasome involves a two-step process consisting of priming and subsequent activation by signal 2 stimuli such as potassium ionophores [2, 4]. Typically, BLPs are commonly used as a priming stimulus through TLR2 and are considered insufficient on their own to induce K^+^ efflux. Therefore, the mechanism by which K^+^ efflux is triggered in THP-1 cells stimulated with BLPs remains unclear. Previous reports have demonstrated that several K^+^ channels play a critical role in ATP-induced NLRP3 activation [34, 35]. However, tetraethylammonium (TEA), tetrapentylammonium (TePA), and quinine, inhibitors of these K^+^ channels, did not significantly affect BLP-induced pyroptosis or caspase-1 activation (Fig. 5A-D). During regulated necrosis, such as pyroptosis or necroptosis, endogenous cellular machinery compromises the plasma membrane [5, 36, 37]. Among these pathways, pores formed by MLKL (mixed lineage kinase domain-like) or GSDMD have been proposed to induce K^+^ efflux, thereby activating the NLRP3 inflammasome [8, 38–40]. Ionic fluxes can also occur during ferroptosis [41, 42]. We thus hypothesized that a distinct execution mechanism of regulated necrosis contributes to BLP-induced NLRP3 activation. The necroptosis inhibitors GSK872 and necrostatin-1 (Nec-1) effectively suppressed necroptosis induced by LPS plus z-VAD-fmk (Supplementary Fig. 5A), but did not inhibit BLP-induced cell death (Fig. 5E). Similarly, the ferroptosis inhibitors deferoxamine (DFO) and ferrostatin-1 (Fer-1) blocked RSL3-induced ferroptosis (Supplementary Fig. 5B), but failed to suppress BLP-induced cell death (Fig. 5F). Furthermore, bafilomycin A1 and chloroquine, which are used as inhibitors of autophagic cell death or lysosomal cell death, did not diminish the cell death response to BLPs (Fig. 5G). In contrast, the disulfidptosis inhibitor dithiothreitol (DTT) markedly reduced BLP-induced cell death, suggesting that disulfidptosis contributes to this response (Fig. 6A) [43].

### Disulfide stress contributes to NLRP3 inflammasome activation

Disulfidptosis is a necrotic form of cell death triggered by a decrease in intracellular NADPH levels, which leads to the accumulation of disulfide bonds among actin cytoskeletal proteins [43, 44]. Excessive crosslinking within the actin cytoskeleton is thought to cause its contraction and collapse, ultimately compromising the plasma membrane as it loses structural support from the cytoskeleton. Because our results suggested that disulfidptosis contributes to BLP-induced inflammasome activation, we further examined this possibility. BLP-induced caspase-1 activation and IL-1β maturation were markedly suppressed when DTT was added to the culture medium (Fig. 6B). Similar results were obtained with other reducing agents, 2-mercaptoethanol (2-ME) and Tris(2-carboxyethyl)phosphine (TCEP) (Supplementary Fig. 6A-C). In contrast, DTT and 2-ME did not affect the up-regulation of pro–IL-1β or phosphorylation of IKKα/β in response to BLPs, indicating that the reducing agents do not inhibit the TLR2 signaling pathway (Fig. 6B, C, and Supplementary Fig. 6B). Furthermore, NLRP3 inflammasome activation induced by the potassium ionophore nigericin was not affected by DTT and 2-ME, demonstrating that they do not interfere with NLRP3 activation itself (Fig. 6D, and Supplementary Fig. 6D). Thus, the inhibitory effects of these reducing agents on BLP-induced NLRP3 inflammasome activation are likely independent of both the downstream signaling events of TLR2 and the NLRP3 inflammasome signaling cascade downstream of K^+^ efflux. Excessive cystine uptake or thiol oxidants such as diamide acts as sensitizers of disulfidptosis by increasing intracellular disulfide bonds [43]. When THP-1 cells were cultured under conditions of cystine overload, BLP-induced cell death was enhanced (Fig. 6E). Similarly, overexpression of SLC7A11, a cystine transporter, increased pyroptosis induction by Pam_3_CSK_4_ in a NLRP3-dependent manner (Fig. 6F, and Supplementary Fig. 7A). THP-1 cells cultured to near confluency expressed higher levels of endogenous SLC7A11 than those re-cultured in fresh medium for 24 h (Supplementary Fig. 7B), correlating with the extent of BLP-induced pyroptosis (Fig. 1E). Furthermore, addition of diamide during BLP stimulation augmented LDH release (Fig. 6G). As diamide failed to enhance LDH release in NLRP3-deficient THP-1 cells, this enhancement was likely to be inflammasome-dependent (Supplementary Fig. 7C). Diamide also promoted BLP-induced caspase-1 activation but did not induce caspase-1 activation alone (Fig. 6H). Disulfidptosis is characterized by reduced intracellular NADPH levels and increased disulfide bonding among actin cytoskeletal proteins [43, 44]. When β-actin from Pam_3_CSK_4_-stimulated THP-1 cells was analyzed by non-reducing SDS-PAGE followed by immunoblotting, bands with decreased electrophoretic mobility appeared at the same time that caspase-1 activation was induced (Fig. 6I). These bands disappeared under reducing SDS-PAGE conditions, suggesting that Pam_3_CSK_4_ stimulation increases disulfide bonding among β-actin proteins. Consistently, Pam_3_CSK_4_ stimulation also caused abnormalities in cortical actin structures (Supplementary Fig. 7D). Additionally, intracellular NADPH levels decreased in Pam_3_CSK_4_-stimulated THP-1 cells (Fig. 6J, K). Taken together, these findings suggest that activation of TLR2 signaling in THP-1 cells generates a cellular environment resembling disulfidptosis, which contributes to NLRP3 inflammasome activation. Consistent with this notion, Pam_3_CSK_4_ stimulation, as well as nigericin, reduced intracellular K^+^ levels, and this reduction was attenuated by reducing agents, suggesting disulfide stress-dependent K^+^ effulx (Fig. 6L, M, and Supplementary Fig. 8A, B).

### Disulfide stress-dependent NLRP3 activation in leukemia cells

**Fig. 7:**
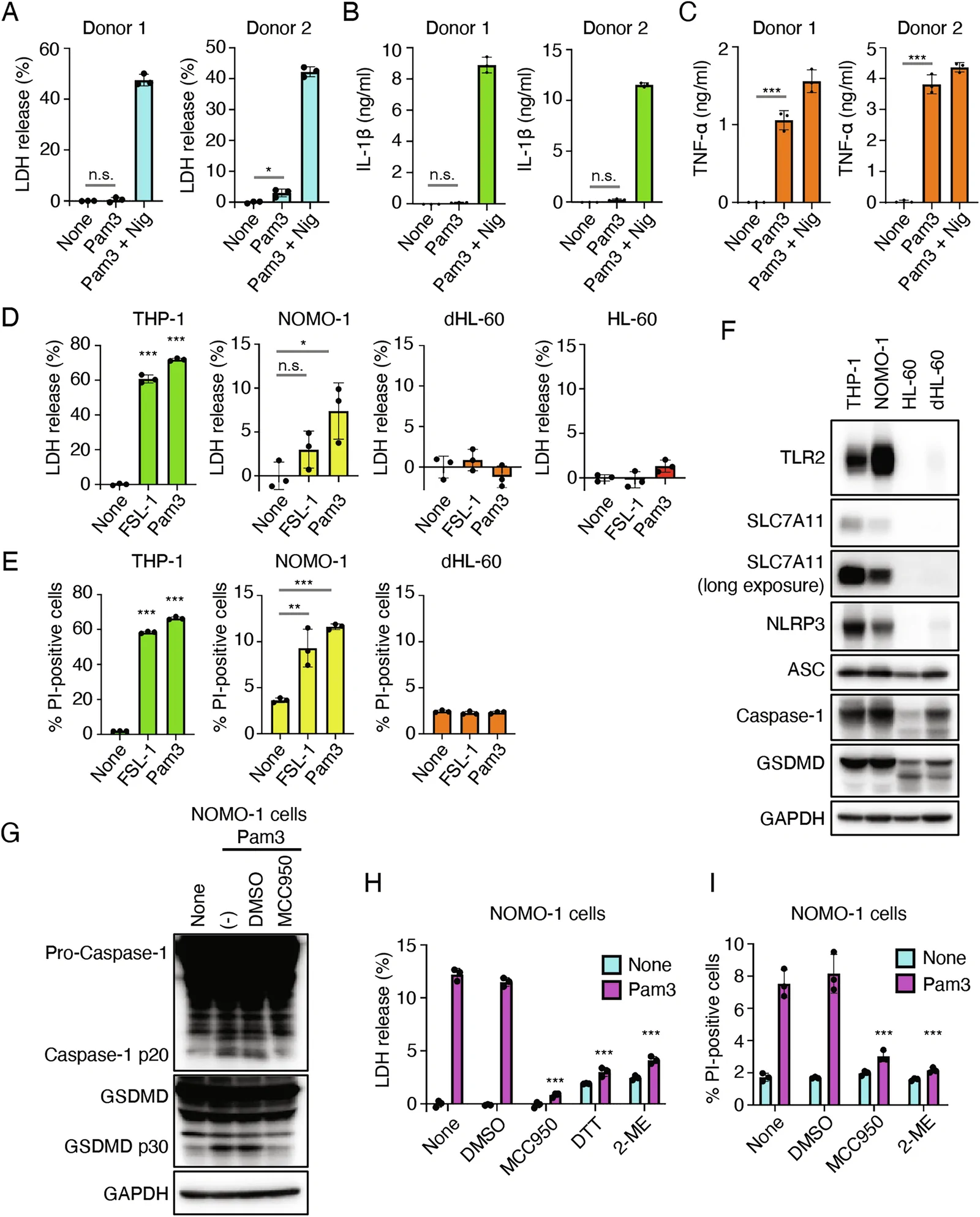
Disulfide stress-dependent NLRP3 inflammasome activation in leukemia cells. **A**–**C** Primary monocytes derived from individual donors were stimulated with Pam3 (500 ng/mL) for 6 h or left unstimulated. Alternatively, nigericin (10 μM) was added after 4 h of Pam3 stimulation, followed by an additional 2 h incubation. LDH release was measured (**A**), and IL-1β (**B**) and TNF-α (**C**) levels in culture supernatants were quantified by ELISA. **D**, **E**, **G**–**I** THP-1, NOMO-1, HL-60, and dHL-60 cells were stimulated with FSL-1 or Pam3CSK4 (500 ng/mL) or left unstimulated for 4 h. In some experiments, NOMO-1 cells were pretreated with MCC950 (10 μM) for 30 min or stimulated in the presence of DTT (0.5 mM) or 2-ME (2 mM) (**G**–**I**). LDH release was measured (**D**, **H**). The proportion of PI-positive cells was analyzed by flow cytometry (**E**, **I**). Immunoblot analysis of caspase-1 and GSDMD in S + L (**G**). **F** Immunoblot analysis of the indicated proteins in cell lysates from the cells shown in D. Data are shown as mean ± SD of triplicate cultures (**A**–**E**, **H**, **I**). Except for **A**–**C**, data are representative of one of three independent experiments with similar results. *P* values were determined using one-way ANOVA followed by Dunnett’s multiple comparisons test (**A**–**E**) or Tukey’s multiple comparisons test (**H**, **I**). *P* values are shown only for comparisons of interest, including between the indicated groups or versus the respective control groups. *P < 0.05; **P < 0.01; ***P < 0.001; n.s., not significant.

Previous reports have shown that human primary monocytes do not undergo pyroptosis in response to Pam_3_CSK_4_ [45, 46]. In our own experiments, primary monocytes robustly secreted TNF-α upon Pam_3_CSK_4_ stimulation, whereas LDH release and IL-1β secretion were minimal (Fig. 7A-C, Supplementary Fig. 9A). In peripheral blood mononuclear cells (PBMCs), Pam_3_CSK_4_ stimulation induced modest LDH release and IL-1β secretion (Supplementary Fig. 9B-D). We next examined whether TLR2 stimulation induces NLRP3 activation in leukemic cell lines other than THP-1. In the monocytic leukemia cell line NOMO-1, Pam3CSK4 stimulation increased LDH release and propidium iodide (PI) uptake, although to a lesser extent than in THP-1 cells, suggesting that a part of the cells underwent necrotic cell death (Fig. 7D, E, and Supplementary Fig. 10A). In contrast, the granulocytic leukemia cell line HL-60 and its DMSO-differentiated form (dHL-60) did not exhibit necrotic cell death upon BLP stimulation. Expression of TLR2, SLC7A11, and NLRP3 was detected in THP-1 and NOMO-1 cells but was markedly lower in HL-60 and dHL-60 cells, which may account for their differential responsiveness to BLPs (Fig. 7F). In Pam_3_CSK_4_-stimulated NOMO-1 cells, activation of caspase-1 and cleavage of GSDMD were observed and were suppressed by MCC950, indicating the involvement of NLRP3 (Fig. 7G). Furthermore, Pam_3_CSK_4_-induced necrotic cell death in NOMO-1 cells was inhibited by MCC950 and reducing agents, suggesting that this response represents disulfide stress-dependent pyroptosis and is mediated by a mechanism similar to that observed in THP-1 cells (Fig. 7H, I, and Supplementary Fig. 11B, C). Finally, NLRP7 mRNA expression in NOMO-1 cells was low, comparable to that in THP-1 cells (Supplementary Fig. 11A), and doxycycline-inducible overexpression of NLRP7 did not enhance Pam_3_CSK_4_-induced pyroptosis (Supplementary Fig. 11B, C). Taken together, TLR2 stimulation induces disulfide stress-dependent pyroptosis via NLRP3, but not NLRP7, in monocytic leukemia cells.

## Discussion

In this study, we demonstrate that bacterial lipopeptides activate the NLRP3 inflammasome and induce GSDMD-dependent pyroptosis in nearly confluent THP-1 cells. This process requires TLR2-IKKβ signaling and K^+^ efflux, despite the general notion that BLPs alone are insufficient to induce K^+^ efflux. Our data also suggest that TLR2 signaling induces a disulfidptosis-like state, and we speculate that this causes sublethal plasma membrane damage that allows K^+^ efflux (Supplementary Fig. 12). To our knowledge, this represents the first example in which disulfidptosis-like processes contribute to innate immune signal transduction.

It is notable that the BLP-induced inflammasome activation observed in this study occurred under particular culture conditions of THP-1 cells and NOMO-1 cells. On the other hand, consistent with a previous report, primary monocytes neither underwent pyroptosis nor released substantial levels of IL-1β following stimulation with Pam_3_CSK_4_ alone (Fig. 7A, B) [45]. Although PBMCs exhibited modest LDH release and IL-1β secretion in response to Pam_3_CSK_4_, it remains unclear whether these responses are attributable to monocytes or other cell populations, warranting further investigation and careful interpretation (Supplementary Fig. 9B, C). Notably, a fraction of PBMCs underwent spontaneous apoptosis irrespective of stimulation, and it cannot be excluded that the observed responses partly reflect artifacts such as DAMP-dependent NLRP3 activation. The absence of BLP-induced pyroptosis in primary monocytes suggests a degree of leukemia cell specificity. THP-1 and NOMO-1 cells are AML-derived lines with oncogenic alterations that may affect metabolic and immune signaling pathways. Moreover, under culture conditions approaching confluency, glucose and other nutrients are likely to be substantially depleted, while metabolic byproducts such as lactate may accumulate. Because glucose is a major source of NADPH production via the pentose phosphate pathway, nutrient depletion may contribute to the TLR2-mediated induction of disulfide stress observed in this study. In addition, Pam_3_CSK_4_ stimulation has been shown to promote assembly of the NADPH oxidase 2 (NOX2) complex in THP-1 cells, resulting in increased ROS production [47]. As NOX2 consumes NADPH to generate ROS, TLR2 stimulation may further reduce intracellular NADPH levels through NOX2 activation. Taken together, responses to TLR2 ligands, altered metabolic conditions, and intrinsic properties of THP-1 cells likely act in concert to drive the reduction in cellular NADPH levels.

Activation of TLR2 signaling by BLPs induced a disulfidptosis-like cellular state. However, THP-1 cells lacking GSDMD or NLRP3 inflammasome components did not undergo BLP-induced cell death, indicating that these disulfidptosis-like alterations were insufficient to cause cell lysis in this model. Disulfidptosis was originally described as a necrotic form of cell death occurring in cells with high expression of the cystine/glutamate antiporter SLC7A11/SLC3A2 under glucose-deprived conditions, where excessive cystine uptake and impaired NADPH supply promote disulfide crosslinking of the actin cytoskeleton [43, 44]. In contrast, THP-1 cells express SLC7A11/SLC3A2 at substantially lower levels than disulfidptosis-sensitive cell lines such as A549 cells, suggesting that full execution of disulfidptosis is unlikely in this context [30]. Indeed, inhibition of glucose uptake by KL-11743 only modestly induced LDH release in THP-1 cells not transduced with DOX-inducible SLC7A11 (Fig. 6F). Moreover, antioxidant stress responses elicited by TLR2-mediated ROS production may render the disulfidptosis-like state transient [48, 49].

Previous studies proposed that BLPs activate an NLRP7-dependent inflammasome in THP-1 cells [22–24]. However, we were unable to reproduce these findings, and our analyses demonstrated that THP-1 cells did not express substantial levels of NLRP7. Consistently, across multiple independent transcriptome datasets (Jin et. al. [30], GDS4137, GDS2251, GDS4781, GDS4258, GDS4041, GDS1604, and GDS4659), NLRP7 transcripts in THP-1 cells were reported to be very low levels (percentile rank <25%), in sharp contrast to the high expression of NLRP3 (percentile rank ~90%). These observations indicate that low NLRP7 expression represents the more typical phenotype of THP-1 cells, highlighting the importance of carefully evaluating NLRP7 expression levels in THP-1 substrains. Moreover, Stehlik and colleagues reported that RNAi-mediated knockdown of NLRP7 markedly reduced BLP-induced IL-1β secretion, whereas Bednash et al. showed that knockdown of either NLRP3 or NLRP7 decreased IL-1β release by approximately 50%, indicating discrepancies among the previous studies [22, 24]. Given that CRISPR/Cas9-based gene disruption is generally considered to have fewer off-target effects than RNAi approaches, we employed CRISPR/Cas9 to disrupt the NLRP7 gene and found that this did not affect BLP-induced pyroptosis. Moreover, while Stehlik’s group demonstrated that stable overexpression of NLRP7 isoform 3 enhanced BLP-induced inflammasome activation in THP-1 cells, we observed no enhancement when either isoform 2 or isoform 3 was expressed [23]. Their experiments utilized constructs driven by the strong CMV-IE promoter, whereas we employed a DOX-inducible system without clonal selection to minimize artifacts from prolonged overexpression of NLRP7 or clonal variation. Furthermore, although Kinoshita et al. reported that transient overexpression of NLRP7 isoform 2 suppresses inflammasome activation by other NLRs, DOX-induced expression of the same isoform did not alter BLP-induced NLRP3 inflammasome activation [27]. Besides, although LPS has been reported to activate the NLRP3 inflammasome in THP-1 cells [24, 26, 47], we observed only minimal activation with ultrapure LPS. In contrast, crude LPS strongly induced IL-1β secretion and LDH release, which were abolished in TLR2-deficient cells (Fig. 4A), indicating that TLR2 ligand contamination likely accounted for the observed inflammasome activation. Therefore, discrepancies between our findings and previous reports likely arise from differences in experimental conditions, THP-1 substrain variability, and reagent quality, reinforcing the need for careful validation of experimental models and reagents.

Although reducing agents such as DTT have been reported to enhance caspase-1 activity by reversing inhibitory oxidative modifications of cysteine residues, including S-sulfhydration, our results showed that DTT suppressed BLP-induced caspase-1 activation [50, 51]. This apparent discrepancy may reflect differences in the level at which DTT acts. In previous studies, DTT was proposed to directly enhance caspase-1 protease activity, whereas in our system, reducing agents appear to act upstream of the NLRP3 inflammasome. Consistently, we observed that DTT inhibited K^+^ efflux, a key trigger for NLRP3 activation.

In summary, we show that BLPs activate the NLRP3 inflammasome in leukemia cells under specific culture conditions and that disulfide stress is a critical upstream contributor. Our findings link disulfidptosis-associated stress to innate immune signaling, revealing that transient, sublethal disulfide stress induced by metabolic conditions and TLR2 signaling can facilitate NLRP3-dependent pyroptosis. This previously unrecognized crosstalk may have implications for strategies aimed at inducing pyroptosis in acute myeloid leukemia.

## Supplementary information

Supplementary Information

Supplementary Figures

Original Raw Data_Western blots

## Data Availability

Primary data and materials are available on request from the corresponding author.
