# Supplementary material for: Sublethal disulfide stress facilitates TLR2-dependent NLRP3 inflammasome activation and pyroptosis in leukemia cells
Source: Cell Death Dis. 2026 Aug 1;17(1):919. doi: 10.1038/s41419-026-09164-4 (PMC13720097; doi:10.1038/s41419-026-09164-4)
Supplement: Supplementary file 1 [file 41419_2026_9164_MOESM2_ESM.pdf]

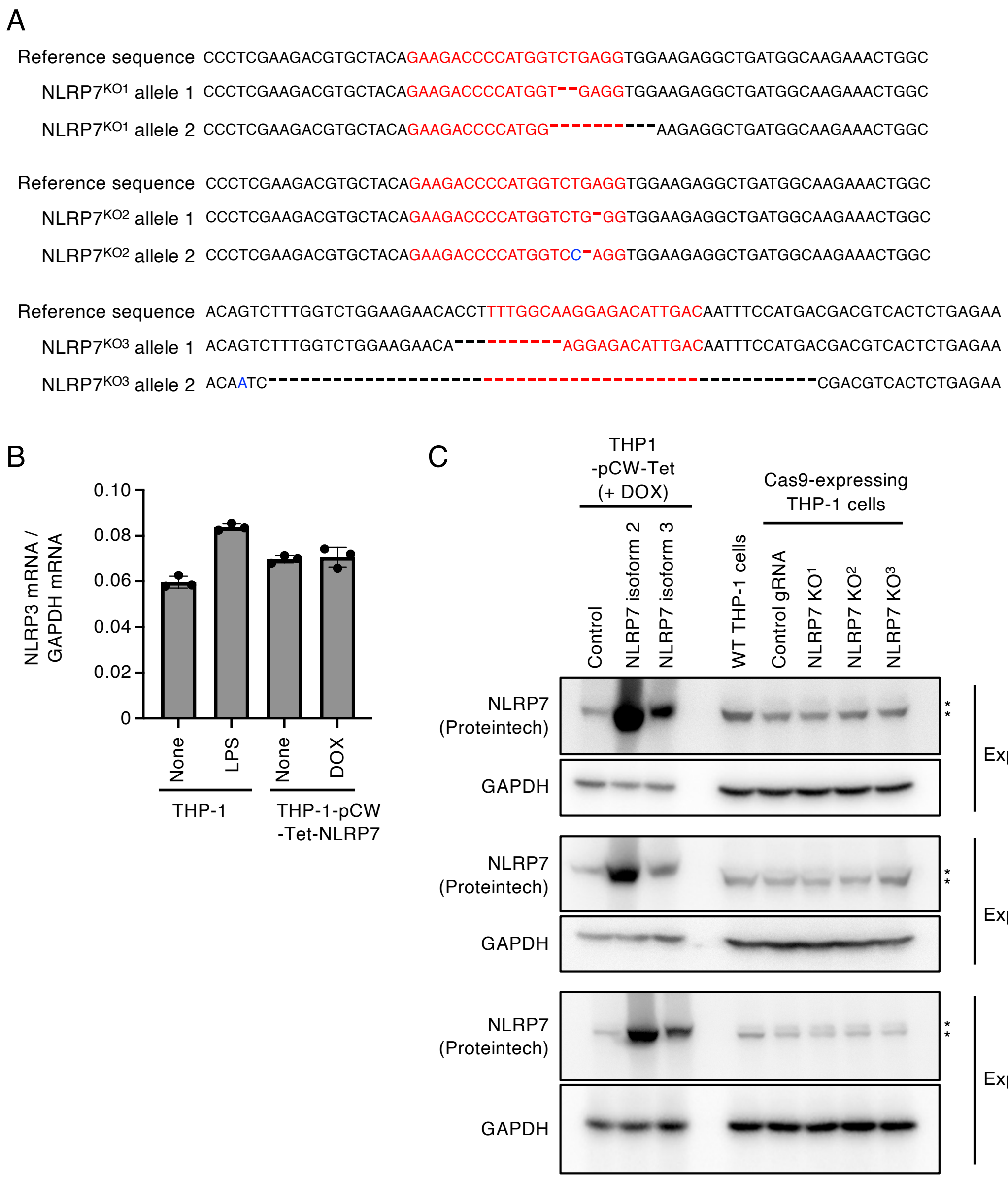

Supplementary Fig. 1. THP-1 cells do not express NLRP7 at substantial levels.
A Genomic sequences within the *NLRP7* locus of each NLRP7 KO THP-1 clone (NLRP7 KO^1, KO^2, and KO^3). The gRNA target sequences are highlighted in red. Dashes indicate deletions, and nucleotide substitutions are shown in blue.
B Quantitative RT-PCR analysis of NLRP3 mRNA expression in WT THP-1 cells stimulated with or without LPS (100 ng/ml) for 24 h or in THP-1 cells transduced with DOX-inducible NLRP7 isoform 2 and treated with or without DOX (100 ng/ml) for 24 h.
C Immunoblot detection of NLRP7 protein in cell lysates from WT THP-1 cells and control and NLRP7 KO THP-1 clones. Cell lysates from THP-1 cells expressing DOX-inducible NLRP7 (isoforms 2 and 3) were used as positive controls. The asterisks (*) indicate nonspecific bands. These bands were detected at comparable levels in control and *NLRP7*-disrupted cells, suggesting that they represent nonspecific signals rather than endogenous NLRP7.
The graphs depict the mean ± SD of triplicate cultures (B). Data are from one representative of (B) or from (C) three biologically independent experiments with similar results.

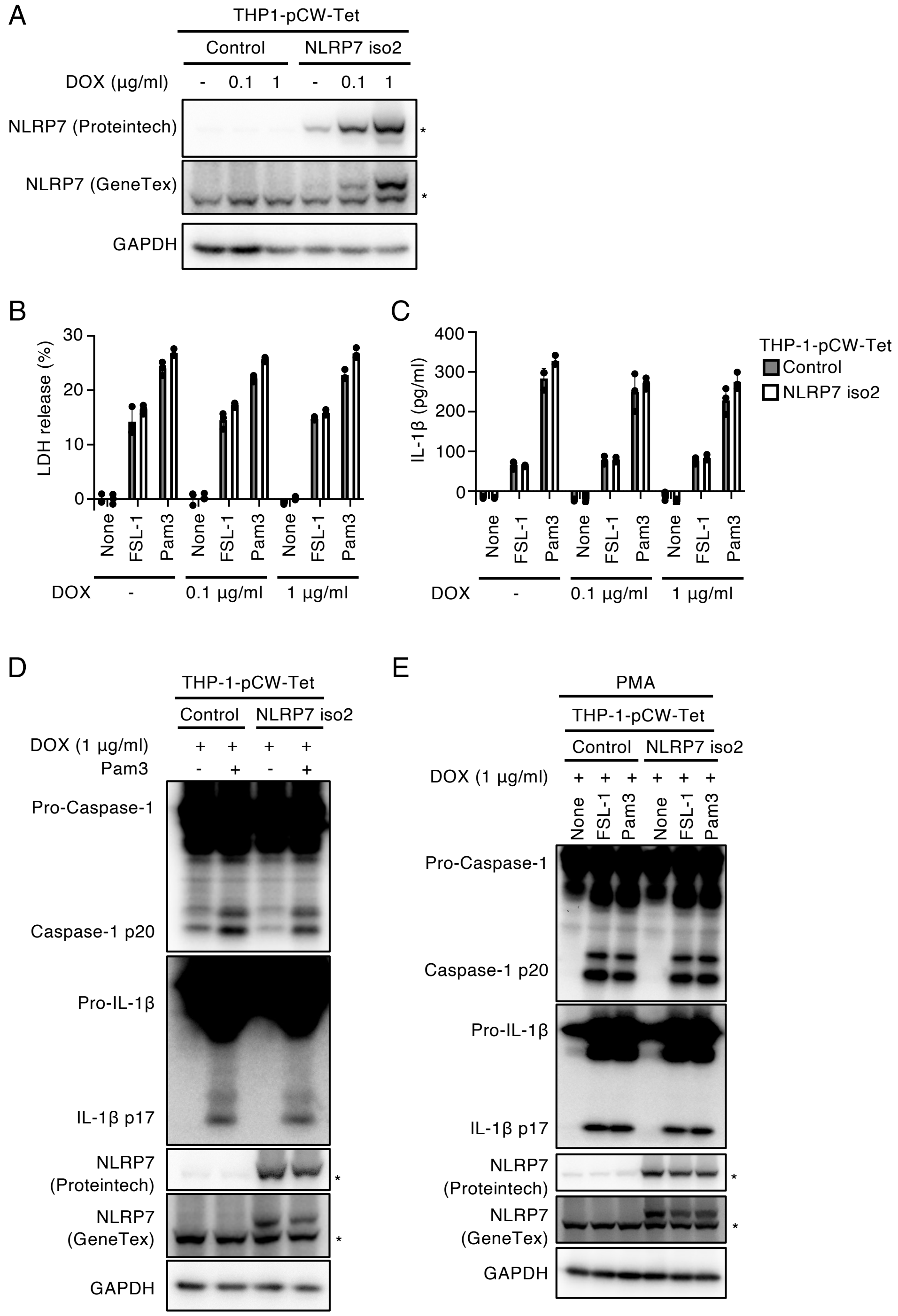

Supplementary Fig. 2. Overexpression of NLRP7 does not enhance BLP-induced inflammasome activation.

A-D Control (DOX-inducible BFP) and DOX-inducible NLRP7 isoform 2-transduced THP-1 cells were treated with DOX for 24 h (A-D) and then stimulated with FSL-1 or Pam3 (500 ng/ml) for 6 h (B-D). Immunoblot analysis of NLRP7 (A, D), caspase-1, and IL-1β (D) in cell lysates (A) or S+L (D). LDH release (B) and IL-1β levels in culture supernatants (C) were measured.

E PMA-differentiated THP-1 cells harboring DOX-inducible NLRP7 isoform 2 or control were treated with DOX (1 µg/ml) for 24 h and then stimulated as in A. Immunoblot analysis in S+L. The asterisks (*) indicate nonspecific bands.

The graphs depict the mean ± SD of triplicate cultures (B, C). Data are from one representative of three biologically independent experiments with similar results.

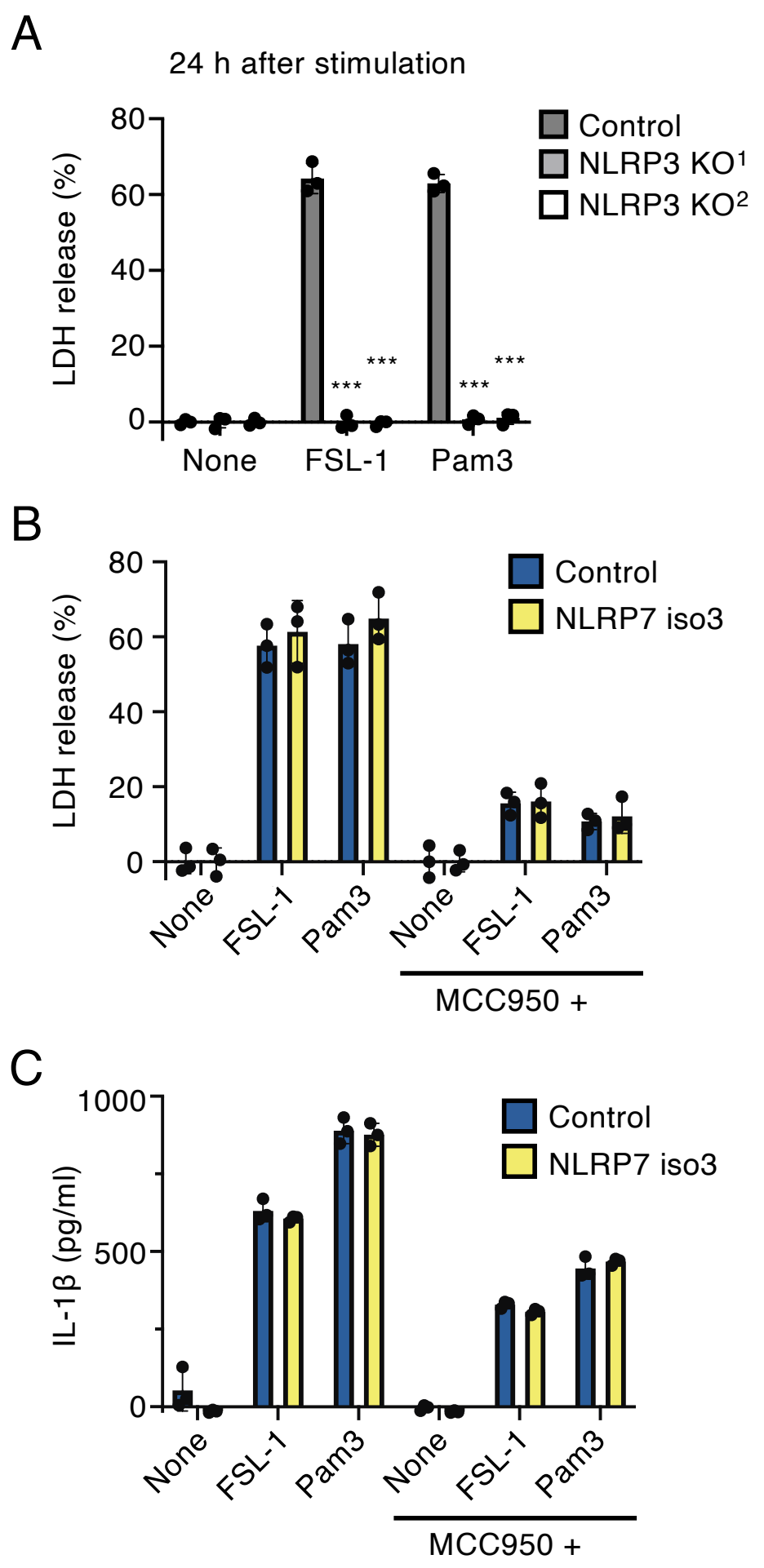

Supplementary Fig. 3. BLP-induced pyroptosis depends on NLRP3.
A Control and NLRP3 KO THP-1 cells (undifferentiated, near-confluent) were stimulated with FSL-1 or Pam3 (500 ng/ml) for 24 h.
B, C THP-1 cells harboring DOX-inducible NLRP7 isoform 3 or control were treated with DOX (1 µg/ml) for 24 h, pretreated with or without MCC950 (10 µM) for 30 min, and then stimulated as in A for 24 h. LDH release assay was performed (A, B). IL-1β levels in culture supernatants (C).
The graph depicts the mean ± SD of triplicate cultures. Data are from one representative of three biologically independent experiments with similar results. P values were determined using two-way ANOVA followed by Dunnett's multiple comparisons test (A). P values are shown only for comparisons of interest, including between the indicated groups or versus the respective control groups. *P < 0.05; **P < 0.01; ***P < 0.001; n.s., not significant.

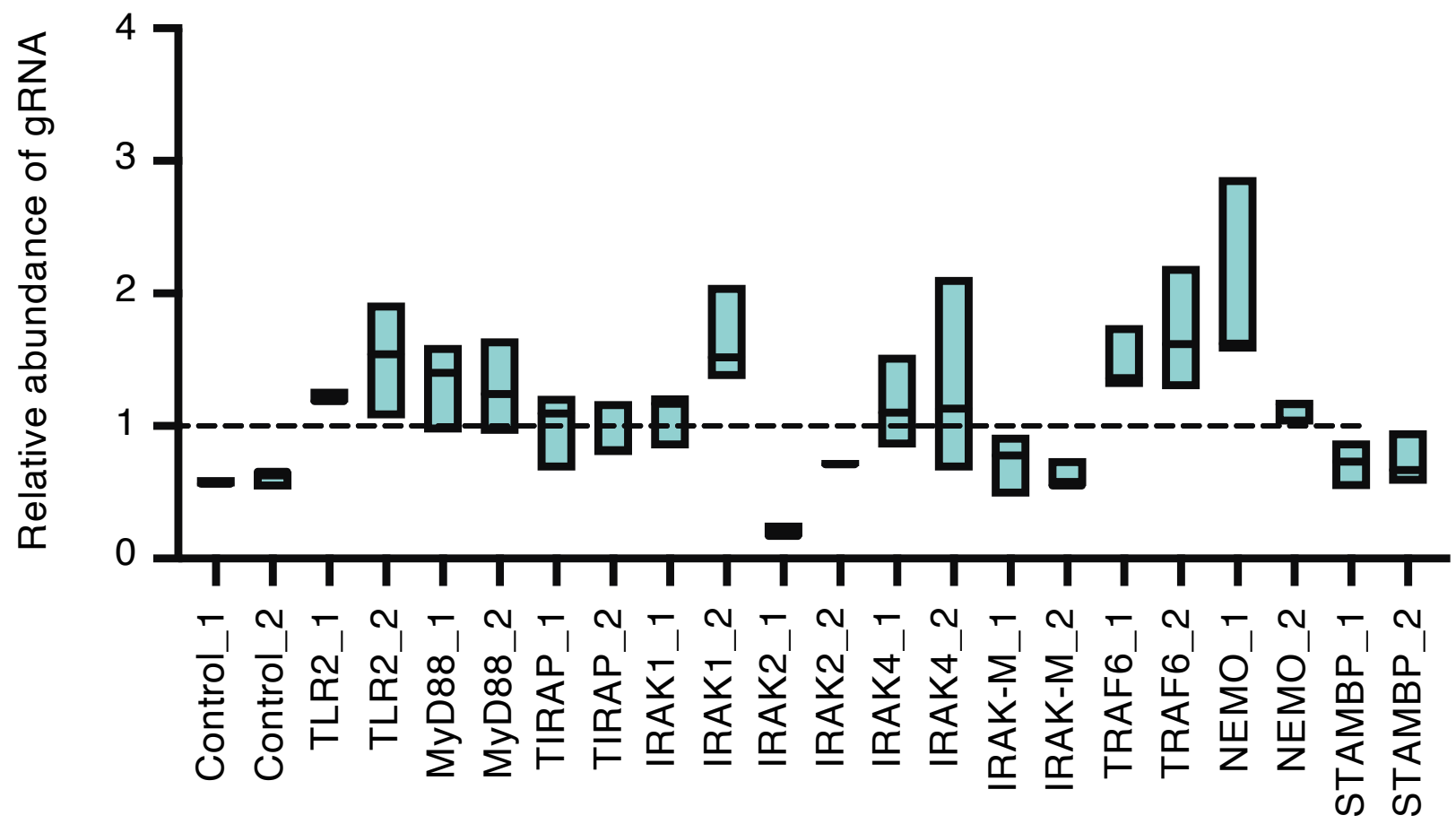

Supplementary Fig. 4. Screening for TLR2 signaling components involved in pyroptosis induction by Pam$_3$CSK$_4$.

A gene-edited THP-1 cell population was generated by introducing a panel of gRNAs targeting TLR signaling components into Cas9-expressing THP-1 cells. The cells were stimulated with Pam3 (500 ng/ml) for 6 h, and surviving cells were isolated. Changes in the relative abundance of each gRNA before and after stimulation were quantified and expressed as fold changes (post-/pre-stimulation). The data correspond to those shown in the heat map in Fig. 4F and are presented here as a floating bar chart. The line within each bar indicates the median. Data are pooled from three biologically independent experiments.

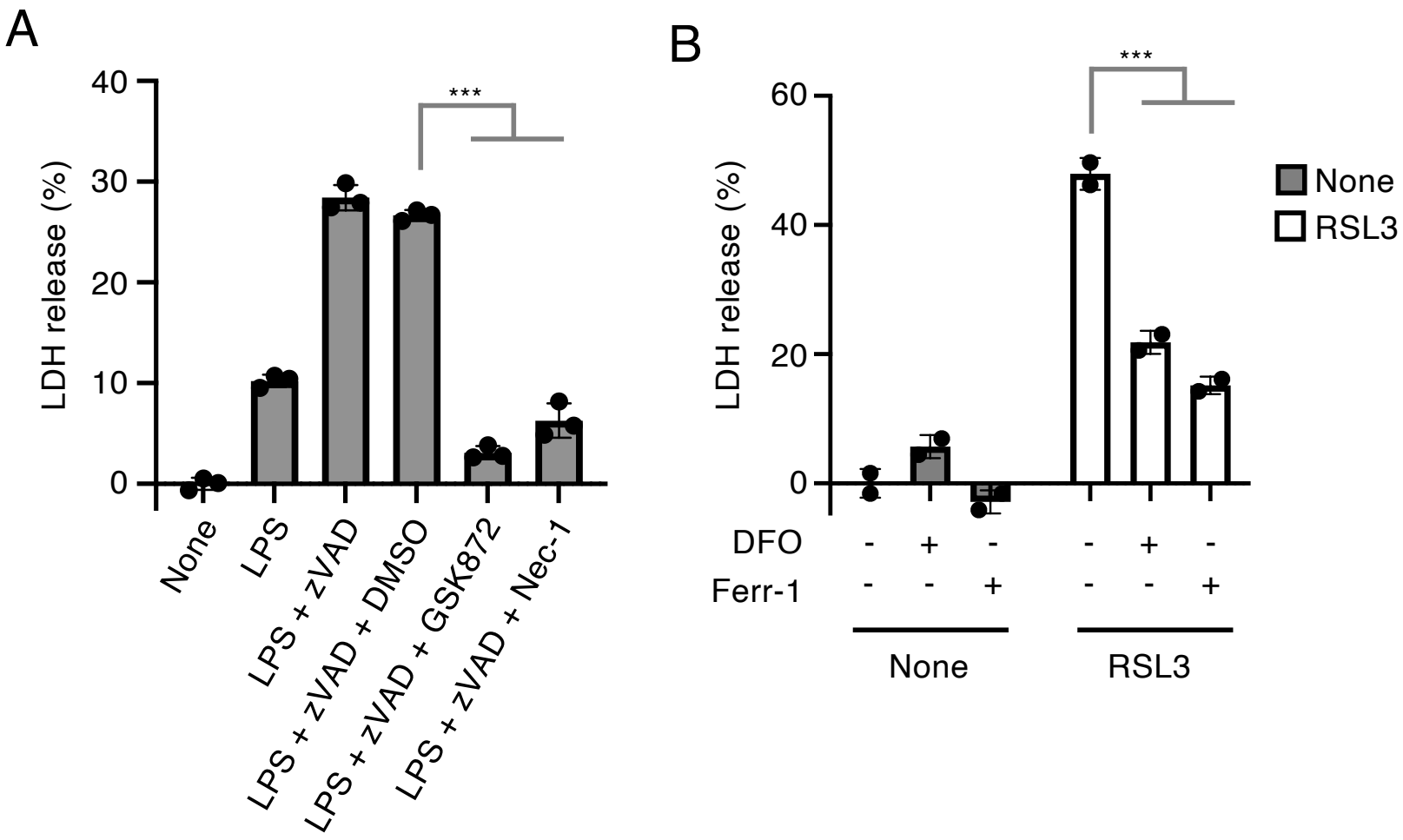

Supplementary Fig. 5. Validation of necroptosis and ferroptosis inhibitors used in this study.

A THP-1 cells were pretreated with the necroptosis inhibitors GSK872 (40 µM) or necrostatin-1 (Nec-1, 40 µM), or with DMSO as a control, for 30 min, and then treated with LPS (100 ng/ml) plus z-VAD-fmk (30 µM) for 24 h to induce necroptosis.

B THP-1 cells were pretreated with the ferroptosis inhibitors DFO (100 µM) or ferrostatin-1 (Ferr-1, 20 µM), or with DMSO as a control, for 30 min, and then treated with RSL3 (20 µM) for 24 h to induce ferroptosis.

Cell lysis was monitored by LDH release assay (A, B). The graphs depict the mean ± SD of duplicate (B) or triplicate (A) cultures. Data are from one representative of two biologically independent experiments with similar results. P values were determined using one-way (A) or two-way (B) ANOVA followed by Dunnett's multiple comparisons test. P values are shown only for comparisons of interest, including between the indicated groups or versus the respective control groups. $^{*}P < 0.05$; $^{**}P < 0.01$; $^{***}P < 0.001$; n.s., not significant.

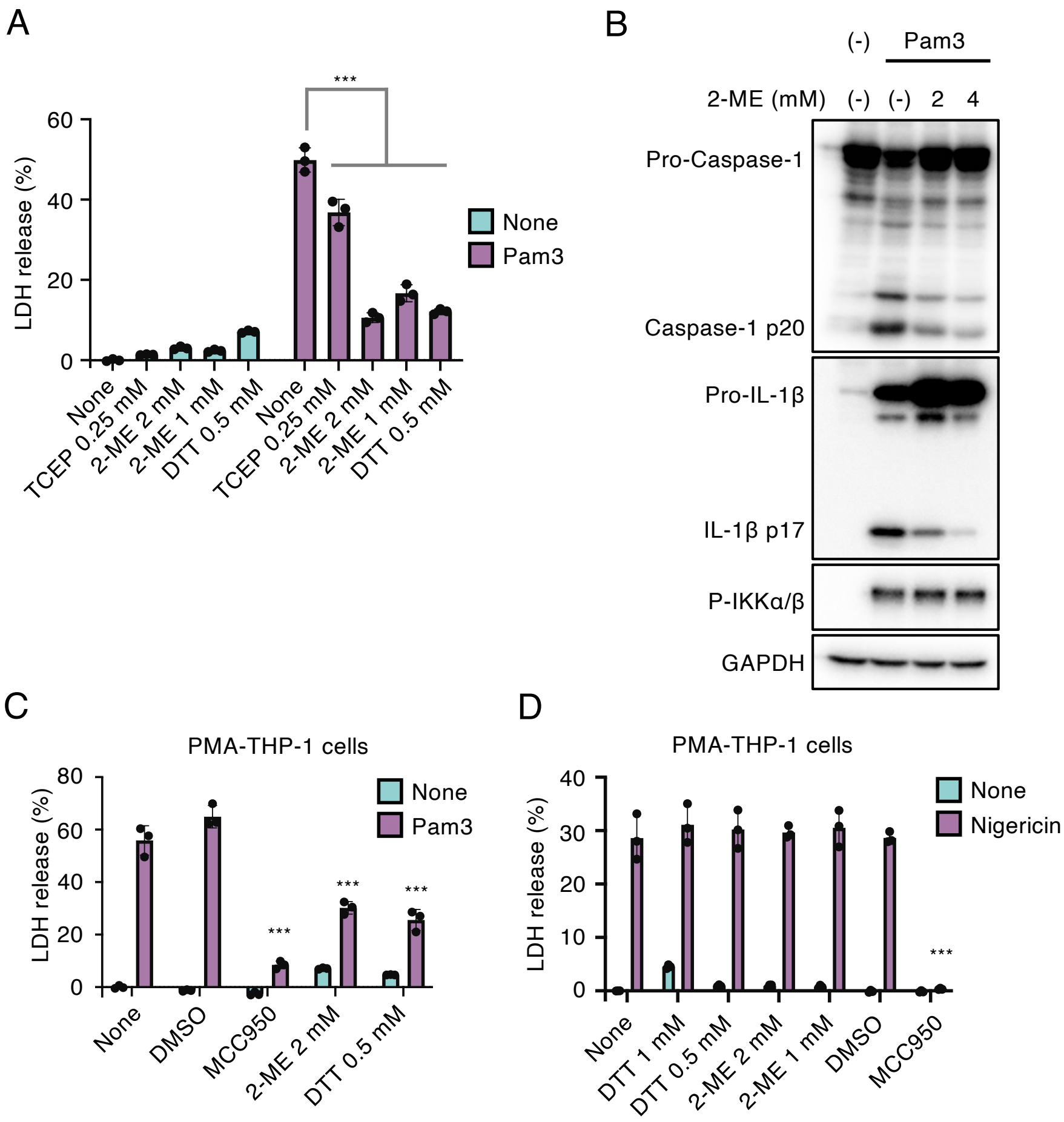

Supplementary Fig. 6. Effects of reducing agents on BLP- or nigericin-induced NLRP3 activation.

A-D THP-1 cells (A), PMA-differentiated THP-1 cells (C, D), or GSDMD KO THP-1 cells (B) were stimulated with 500 ng/mL of Pam3 (A-C) or 10 µM of nigericin (D) for 60 min (B), 90 min (A, D) or 3 h (C) in the presence or absence of the indicated concentrations of reducing agents. LDH release assay was performed (A-C). Immunoblot analysis of caspase-1, IL-1β, and phosphorylated IKKα/β in cell lysates (B).

The graphs depict the mean ± SD of triplicate cultures. Data are from one representative of three biologically independent experiments with similar results. Data are from one representative of three biologically independent experiments with similar results. P values were determined using two-way ANOVA followed by Dunnett's multiple comparisons test (A, D) or Tukey's multiple comparisons test (C). P values are shown only for comparisons of interest, including between the indicated groups or versus the respective control groups. *P < 0.05; **P < 0.01; ***P < 0.001; n.s., not significant.

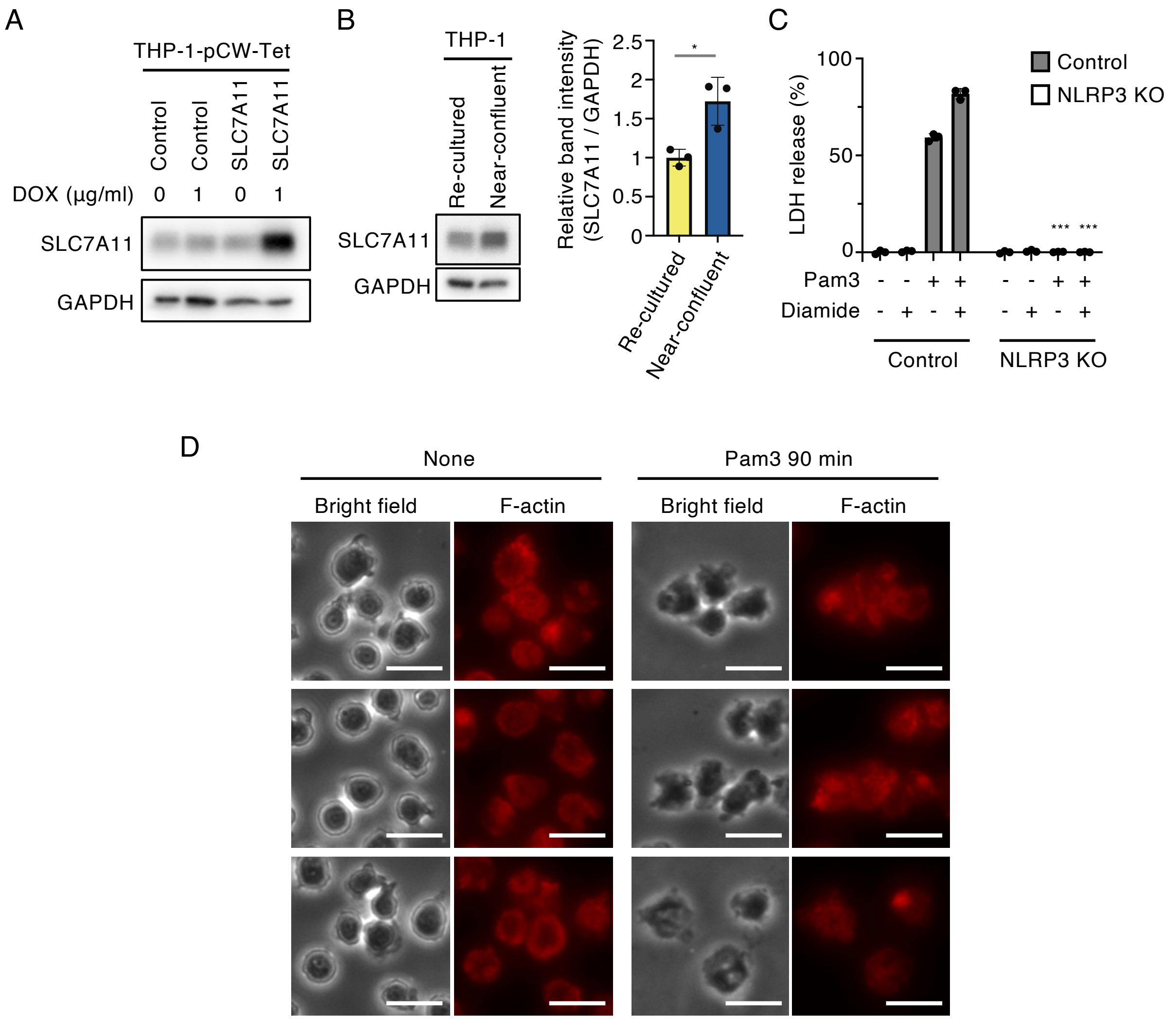

Supplementary Fig. 7. NLRP3 is essentially required for the enhancement of Pam_3CSK_4-induced pyroptosis by diamide.

A Immunoblot analysis of SLC7A11 in cell lysates from control (DOX-inducible BFP) and DOX-inducible SLC7A11-transduced THP-1 cells treated with or without DOX for 24 h.

B Immunoblot analysis of SLC7A11 in THP-1 cells cultured in fresh medium for 1 day (Re-cultured) or 7 days (Near-confluent). Band intensity was quantified (right panel).

C Control and NLRP3 KO THP-1 cells were stimulated with Pam3 (500 ng/ml) for 3 h. Diamide was added to the cultures 30 min after stimulation. LDH release assay was performed.

D Fluorescence microscopy images of F-actin staining in PMA-differentiated GSDMD KO THP-1 cells stimulated with or without Pam3 for 90 min. Scale bars, 20 µm.

The graphs depict the mean ± SD of triplicate cultures (B, C). Data are from one representative of three biologically independent experiments with similar results. P values were determined using an unpaired t test with Welch's correction (B, C). P values are shown only for comparisons of interest, including between the indicated groups or versus the respective control groups. *P < 0.05; **P < 0.01; ***P < 0.001; n.s., not significant.

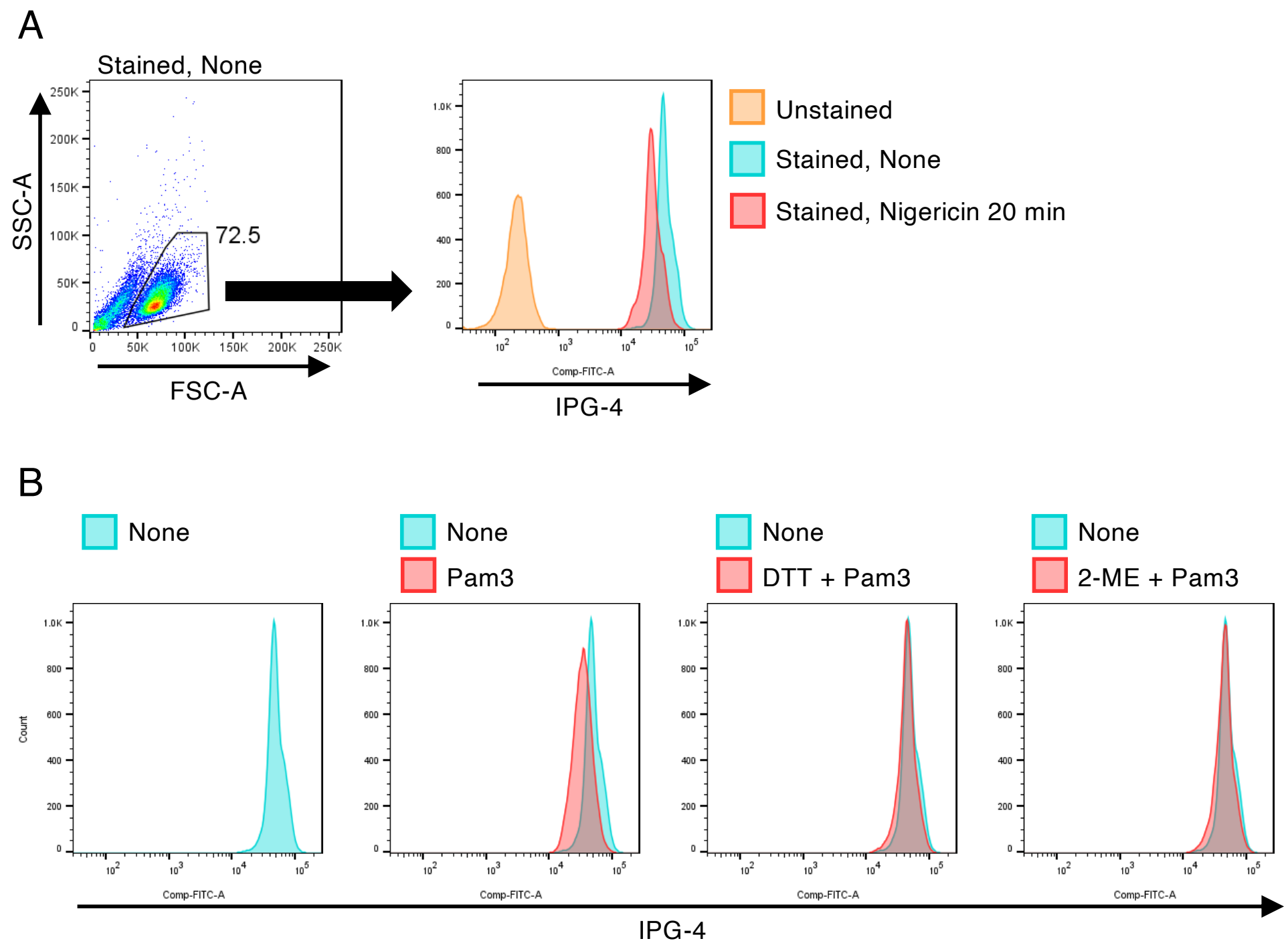

Supplementary Fig. 8. Disulfide stress-dependent K^+ efflux upon TLR2 stimulation.

A, B GSDMD KO THP-1 cells were stained with or without IPG-4 AM and stimulated with or without nigericin (10 µM) for 20 min (A) or Pam3 (500 ng/ml) for 90 min (B). Left panel and right panel show gating strategy and representative histograms of IPG-4 fluorescence, respectively (A). Representative histograms of IPG-4 fluorescence in cells stimulated with or without Pam3 in the presence or absence of DTT (0.5 mM) or 2-ME (2 mM) (B). The same unstimulated control histogram is overlaid in each panel to allow direct comparison with each treatment condition (B). Data are from one of three independent experiments with similar results.

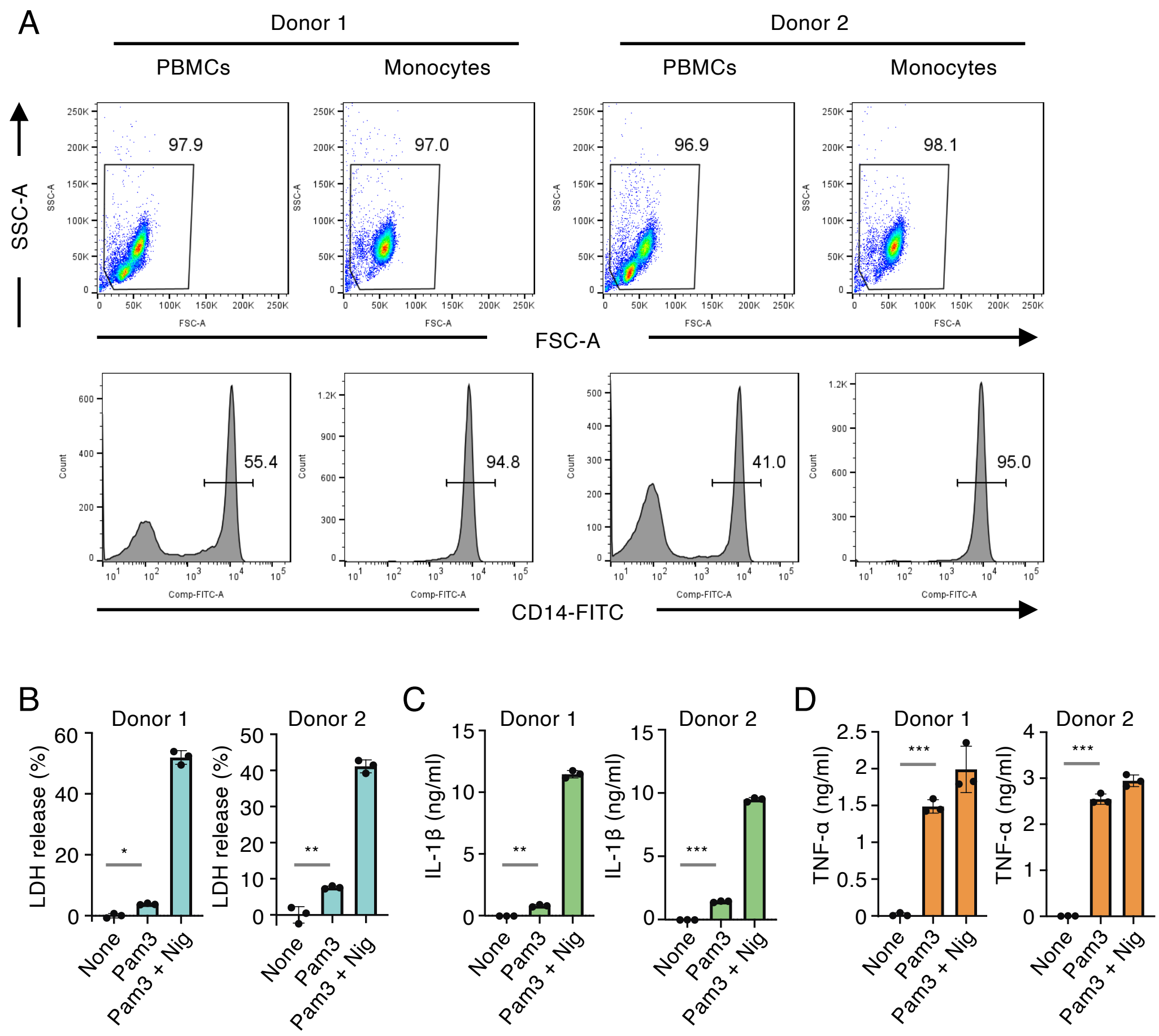

Supplementary Fig. 9. Responses of PBMCs to TLR2 stimulation.
A Flow cytometric analysis of PBMCs and enriched monocytes derived from individual donors. Upper panels: forward scatter versus side scatter dot plots with gating strategy. Lower panels: histograms of CD14-FITC staining.
B-D PBMCs were stimulated with Pam3 (500 ng/mL) for 6 h or left unstimulated. Alternatively, nigericin (10 µM) was added after 4 h of Pam3 stimulation, followed by an additional 2 h incubation. LDH release was measured (B), and IL-1β (C) and TNF-α (D) levels in culture supernatants were quantified by ELISA.
The graphs depict the mean ± SD of triplicate cultures (B-D). P values were determined using one-way ANOVA followed by Dunnett's multiple comparisons test (B-D). P values are shown only for comparisons of interest, including between the indicated groups or versus the respective control groups. $^{*}P < 0.05$; $^{**}P < 0.01$; $^{***}P < 0.001$; n.s., not significant.

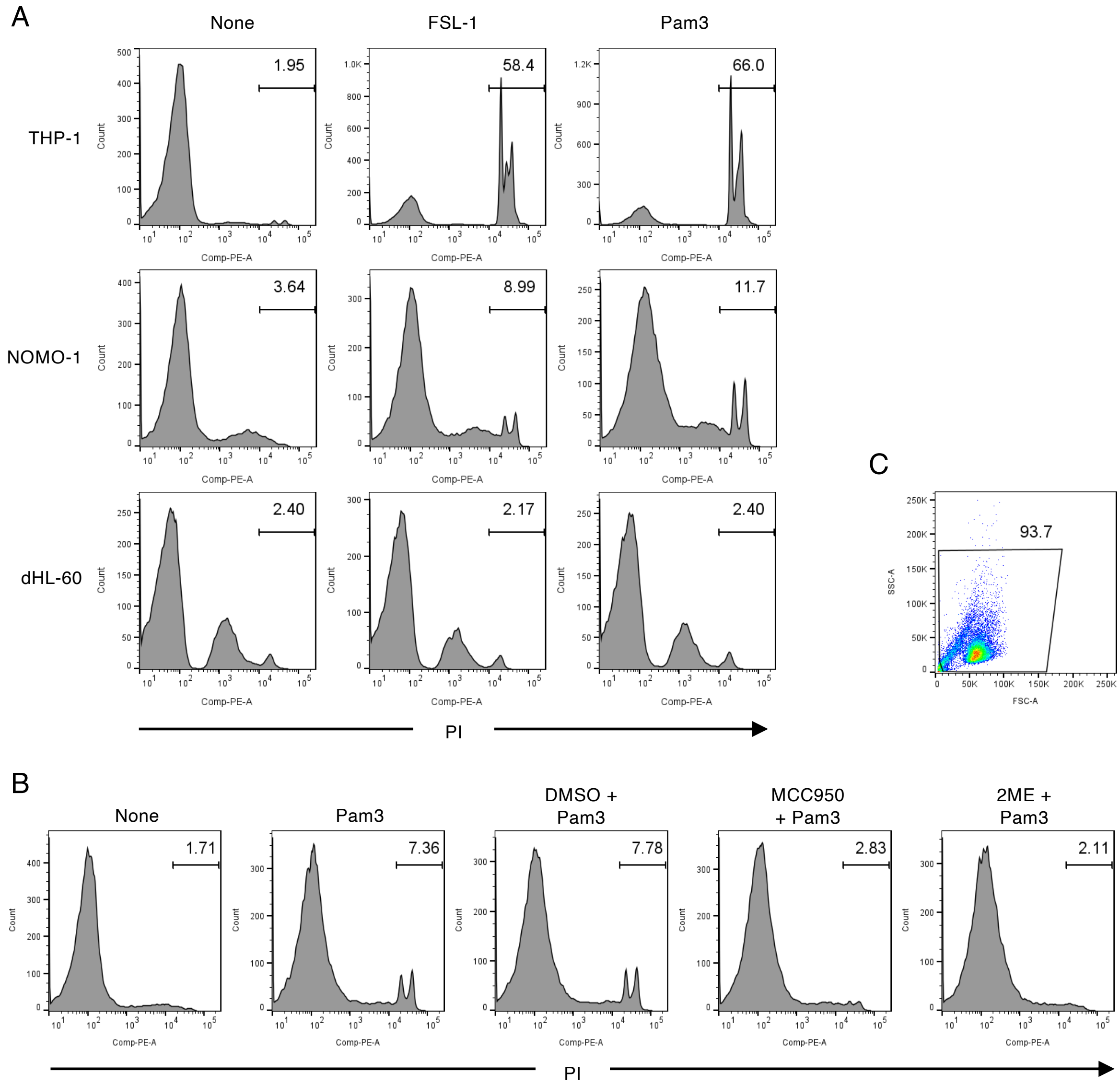

Supplementary Fig. 10. Differential induction of pyroptosis among leukemia cell lines following TLR2 stimulation.

A, B THP-1, NOMO-1, and dHL-60 cells were stimulated with FSL-1 or Pam3CSK4 (500 ng/mL) or left unstimulated for 4 h. In some experiments, NOMO-1 cells were pretreated with MCC950 (10 µM) for 30 min or stimulated in the presence of 2-ME (2 mM) (B). Representative histograms of PI fluorescence in PI-stained cells analyzed by flow cytometry are shown (A, B).

C Gating strategy.

Data are representative of three independent biological experiments with similar results.

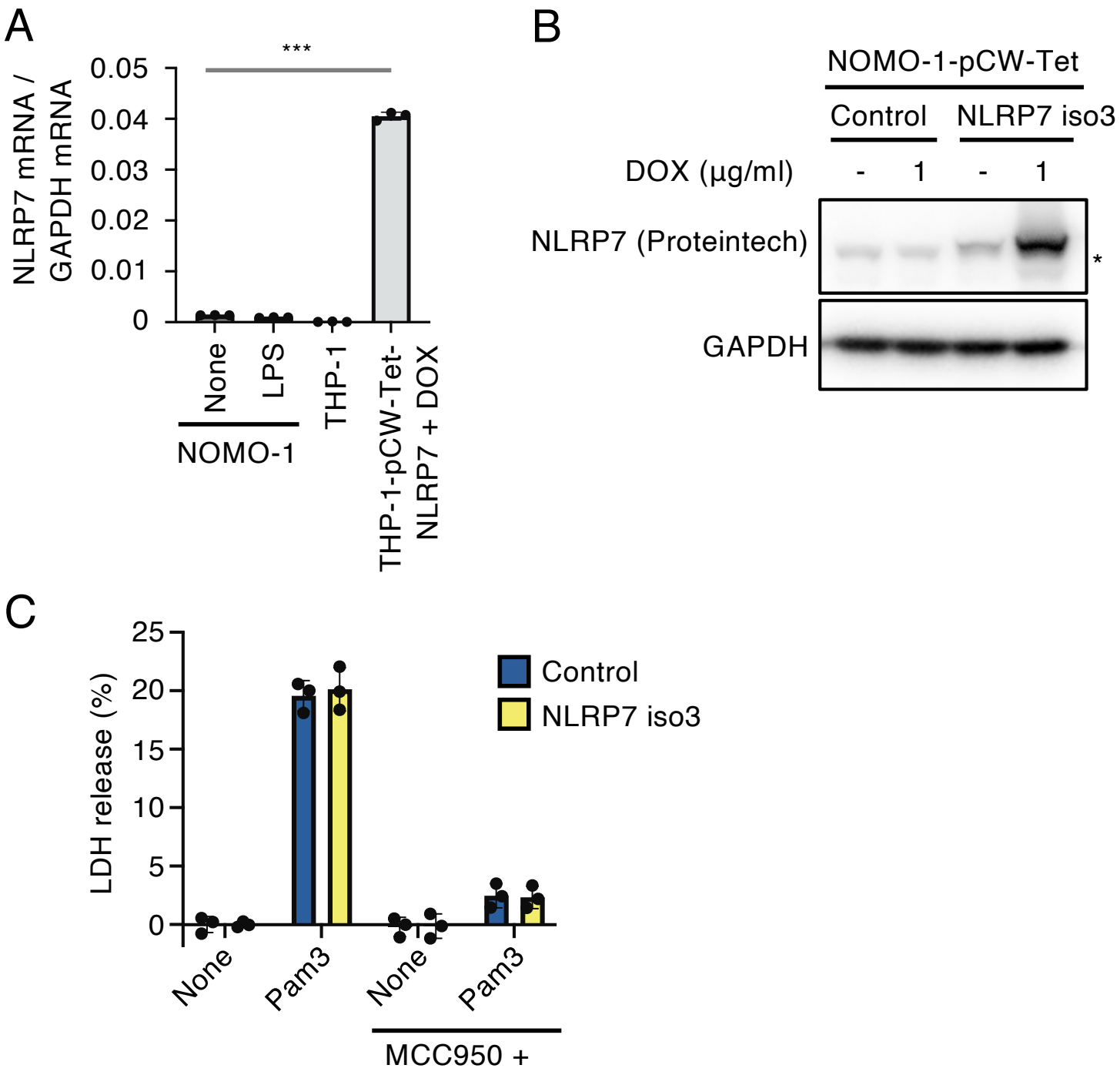

Supplementary Fig. 11. NLRP7 does not promote BLP-induced pyroptosis in NOMO-1 cells.

A Quantitative RT-PCR analysis of NLRP7 mRNA in NOMO-1 cells stimulated with or without LPS (100 ng/ml) for 24 h, alongside THP-1 cells expressing DOX-inducible NLRP7 isoform 2 as a positive control.

B DOX-inducible BFP (as a control) or NLRP7 isoform 3-transduced NOMO-1 cells were treated with DOX for 24 h, and NLRP7 protein levels were analyzed by immunoblotting.

C Cells shown in B were pretreated with or without MCC950 (10 µM) for 30 min and then stimulated with Pam3 (500 ng/mL) or left unstimulated for 4 h. LDH release assay was performed.

The graph depicts the mean ± SD of triplicate cultures. Data are from one of three independent experiments with similar results. P values were determined using one-way ANOVA followed by Dunnett's multiple comparisons test (A). P values are shown only for comparisons of interest, including between the indicated groups or versus the respective control groups. *$P < 0.05$; **$P < 0.01$; ***$P < 0.001$; n.s., not significant.

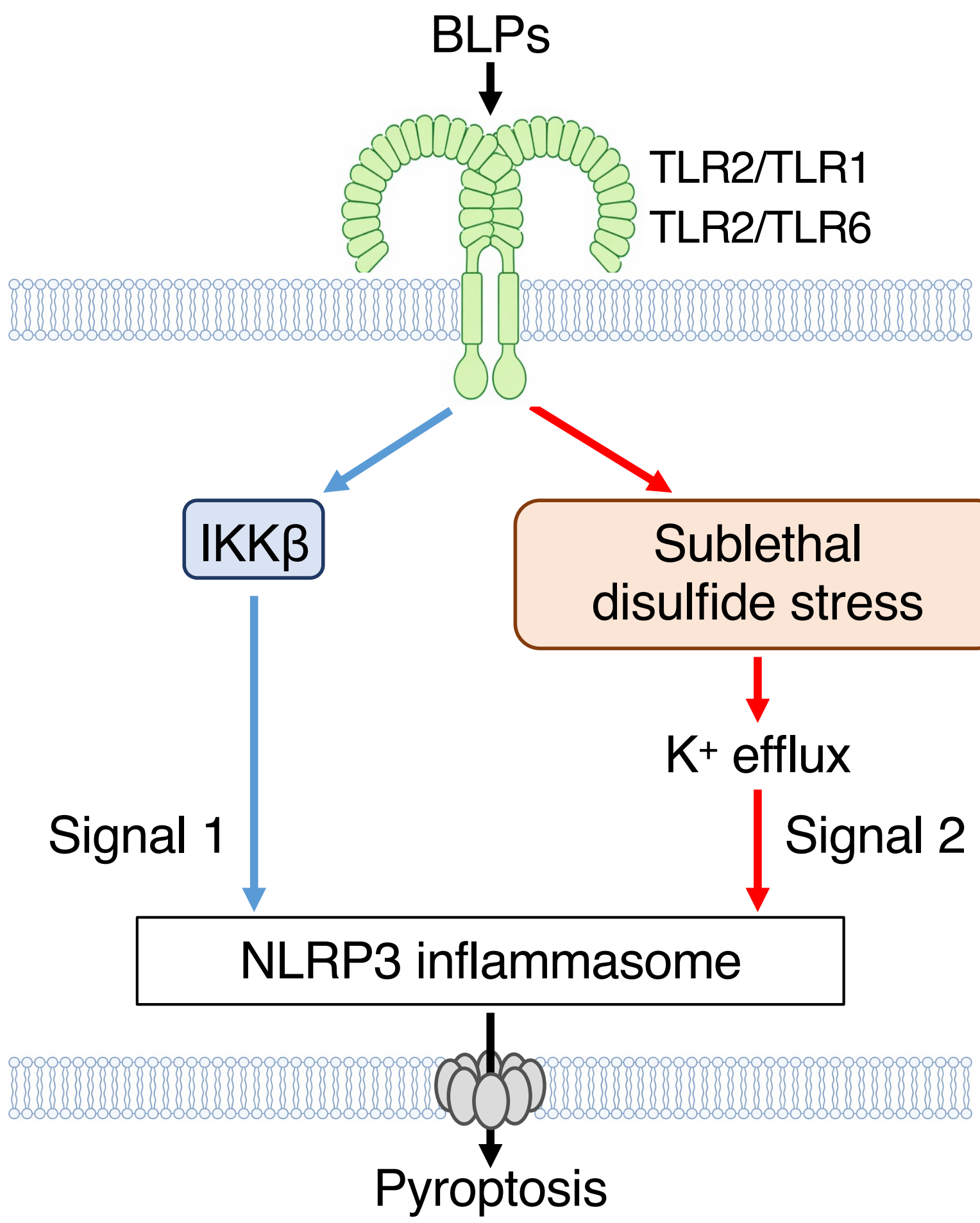

Supplementary Fig. 12. Schematic model proposed in this study.
Under high-density culture conditions in monocytic leukemia cells, TLR2 stimulation by BLPs induces disulfide stress, which promotes K^+ efflux probably through transient plasma membrane damage, thereby leading to NLRP3 inflammasome-dependent pyroptosis.
