# Supplementary material for: Sublethal disulfide stress facilitates TLR2-dependent NLRP3 inflammasome activation and pyroptosis in leukemia cells
Source: Cell Death Dis. 2026 Aug 1;17(1):919. doi: 10.1038/s41419-026-09164-4 (PMC13720097; doi:10.1038/s41419-026-09164-4)

Original Raw Data (Western blots)

Fig. 1B

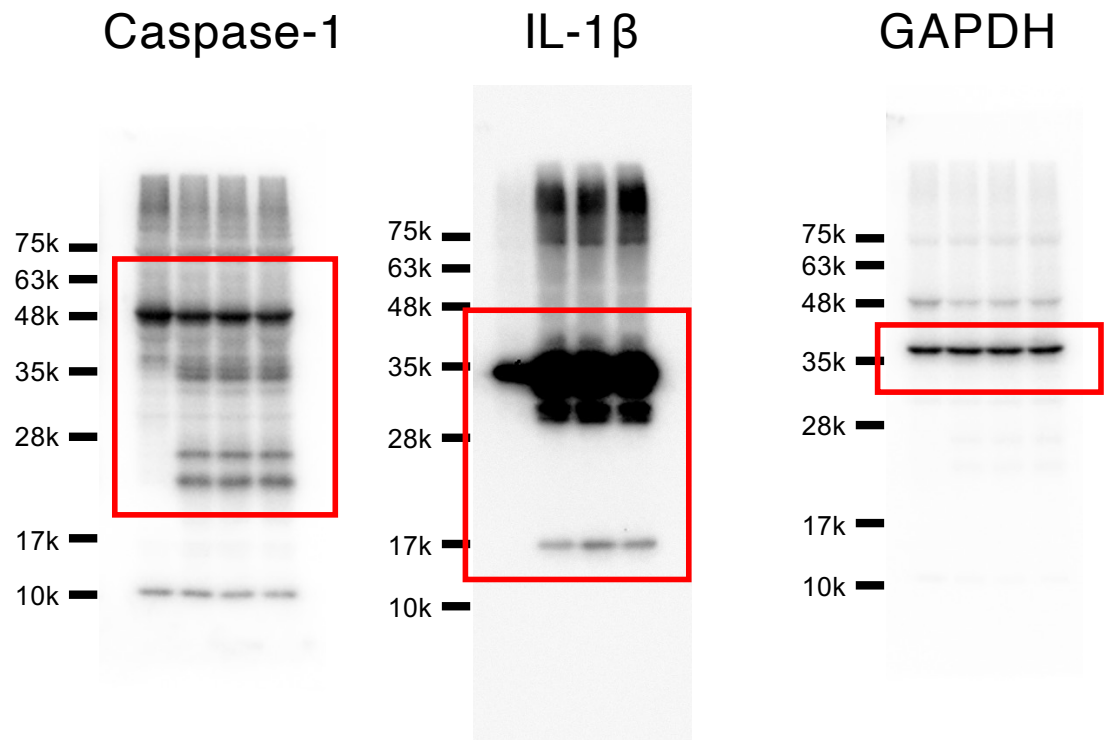

Fig. 1G

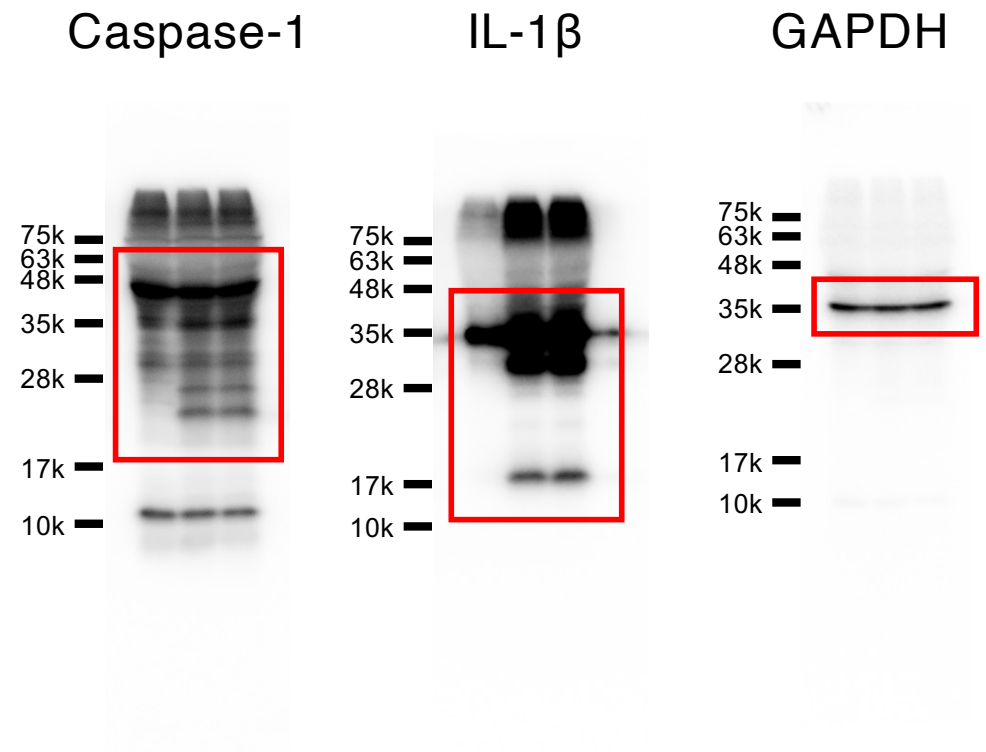

Fig. 1J

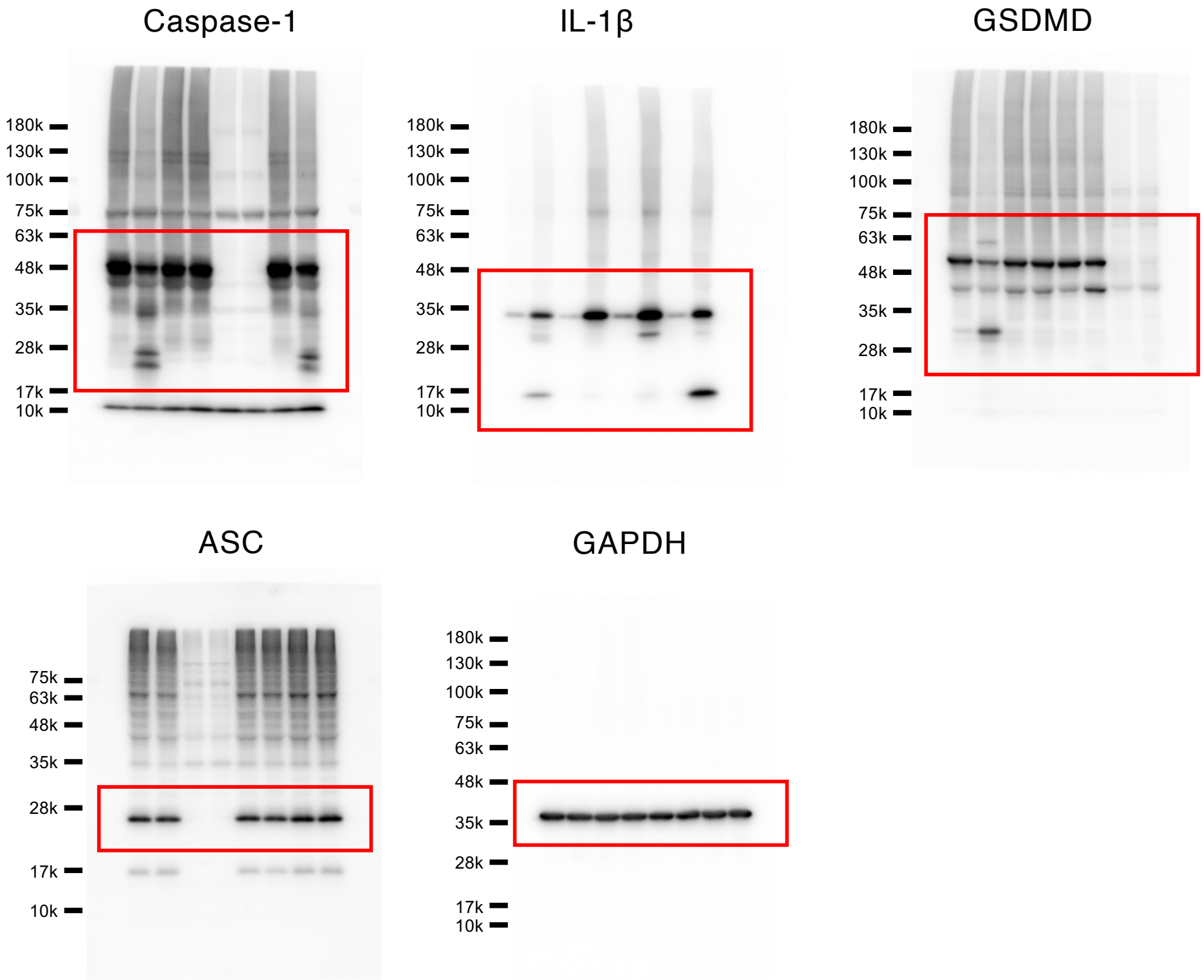

Fig. 2D

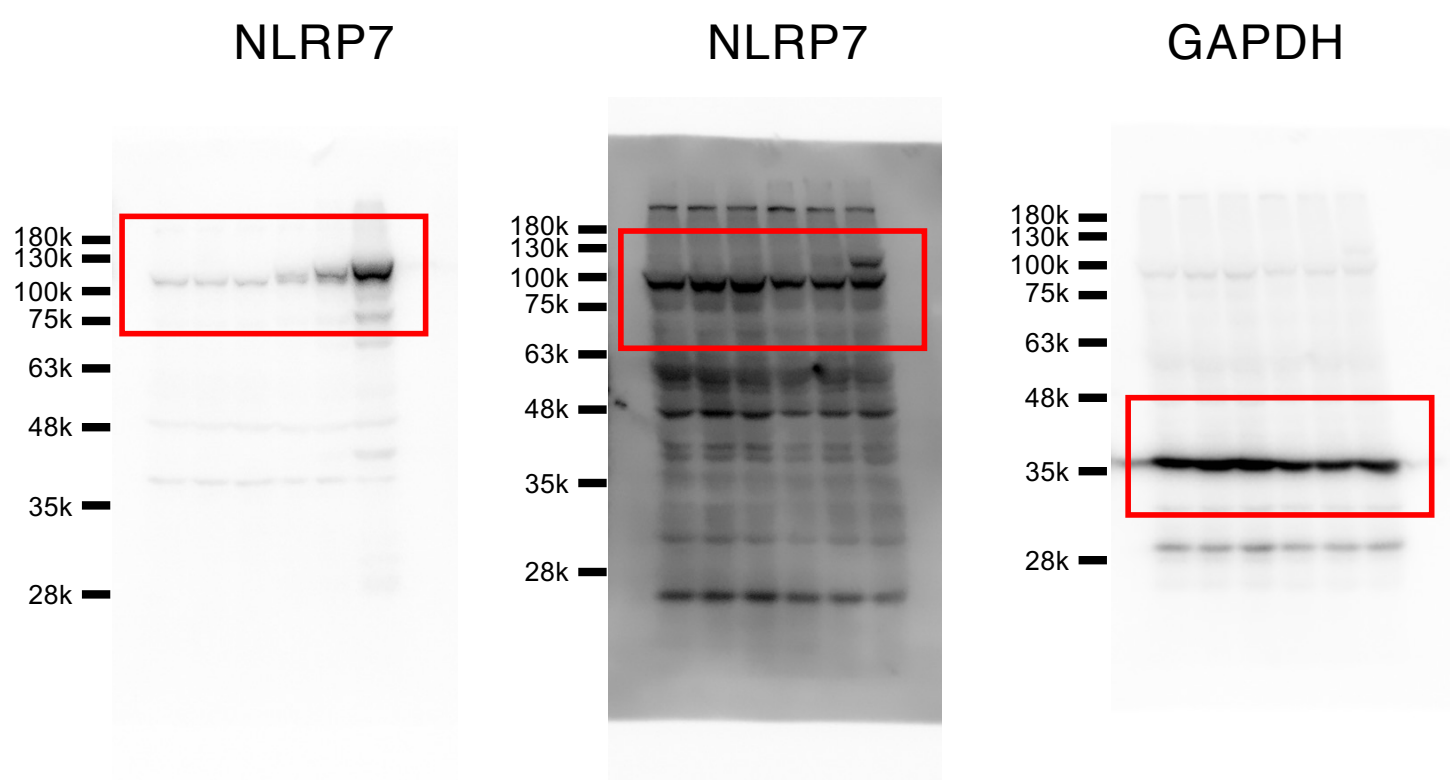

Fig. 2F

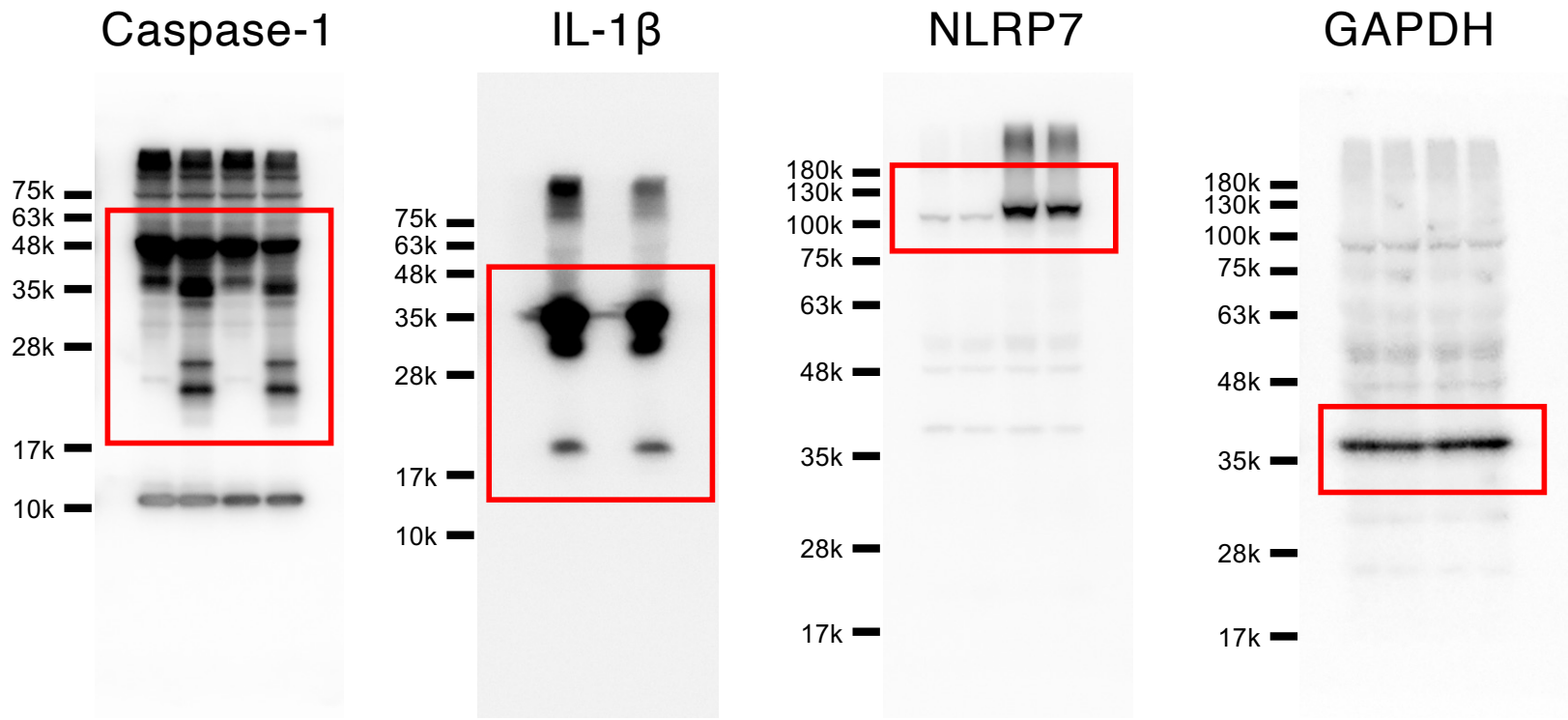

Fig. 2H

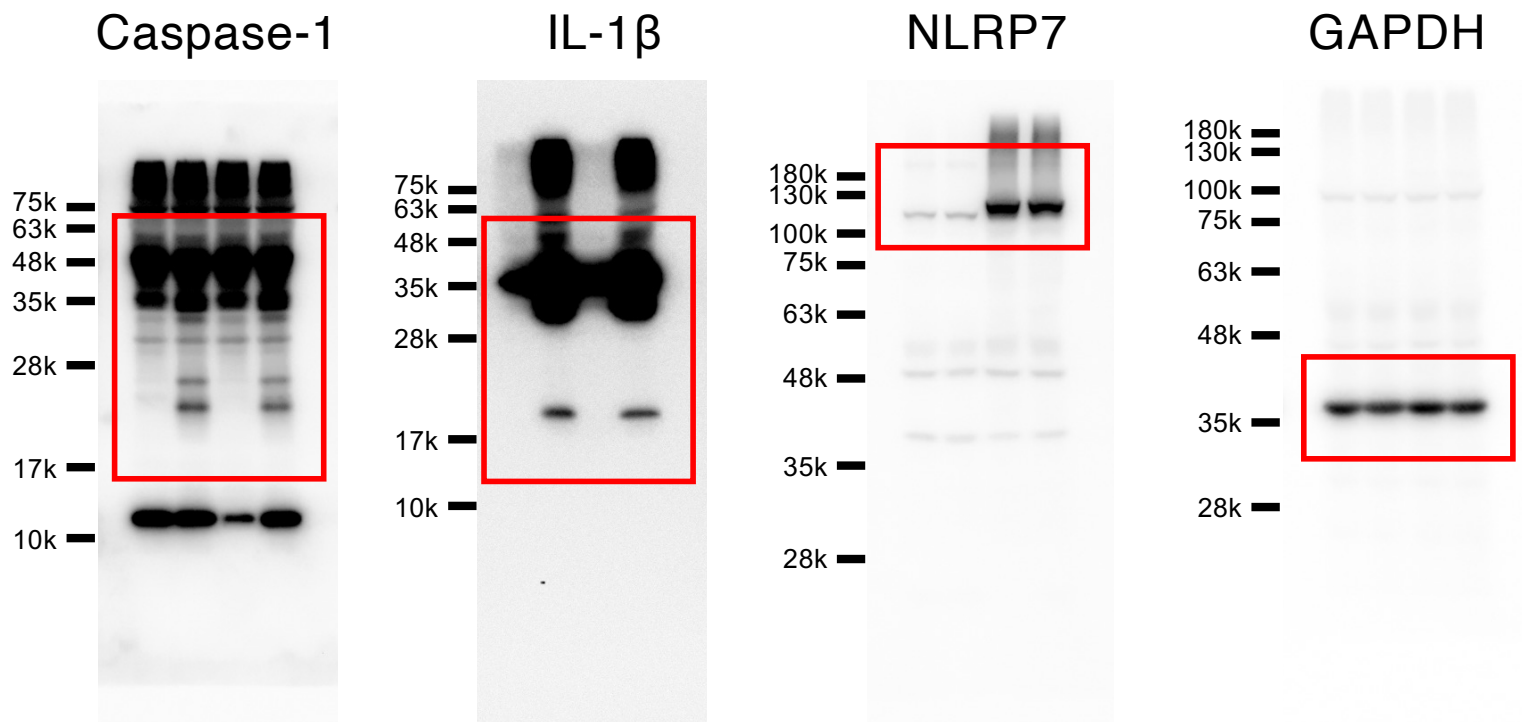

Fig. 3B

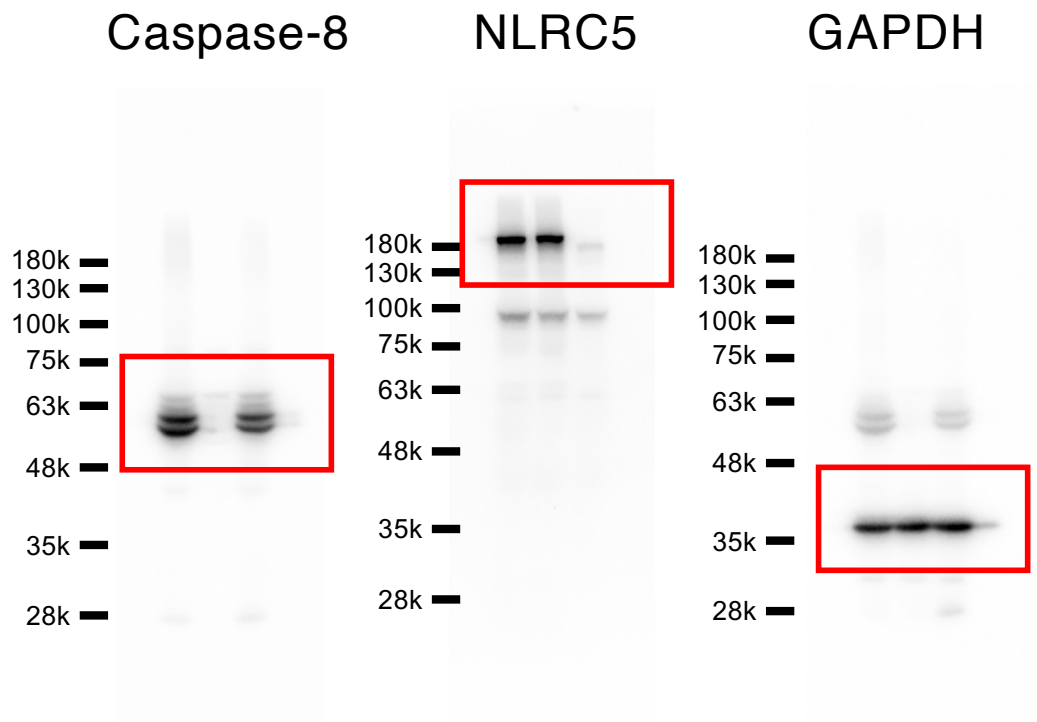

Fig. 3D

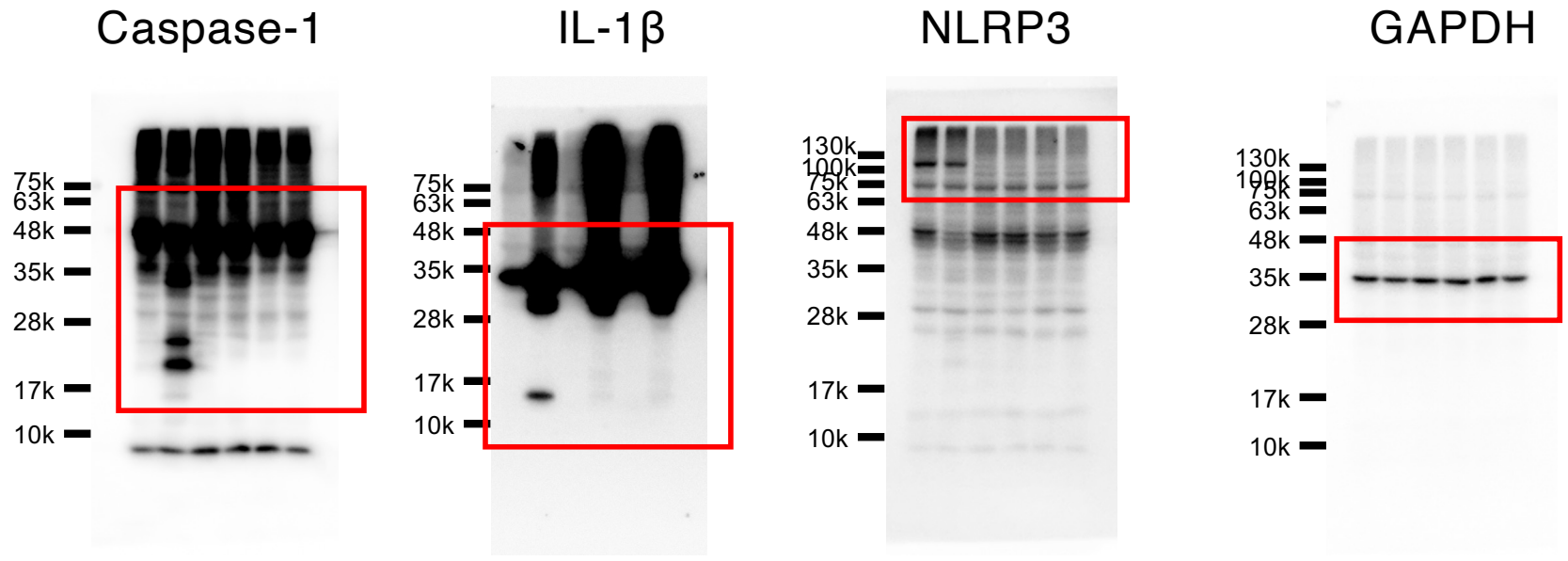

Fig. 3H

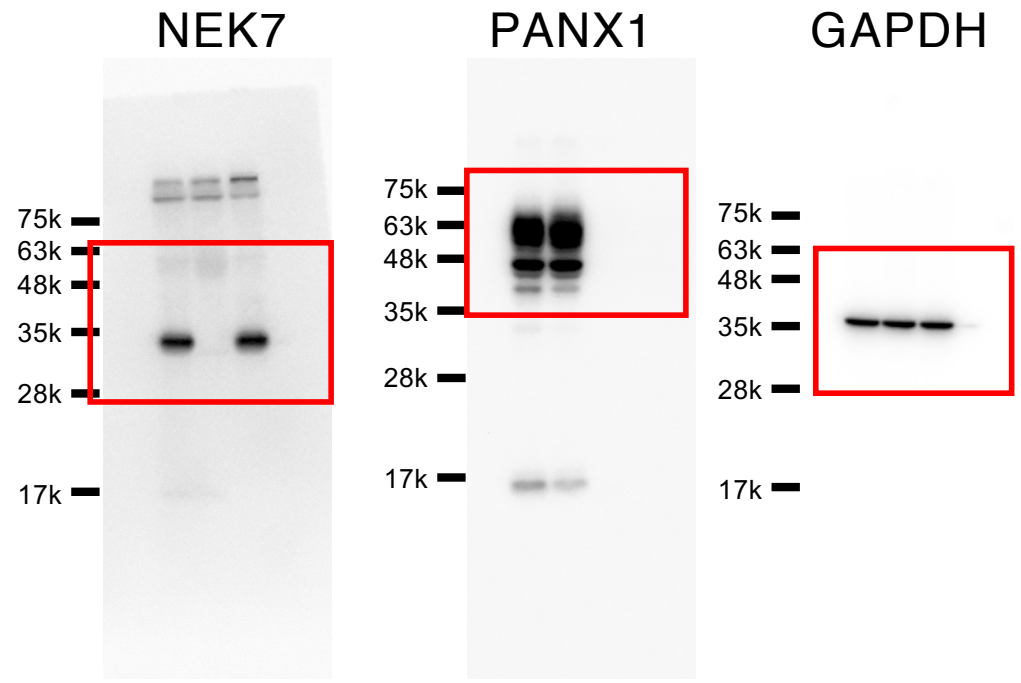

Fig. 4B

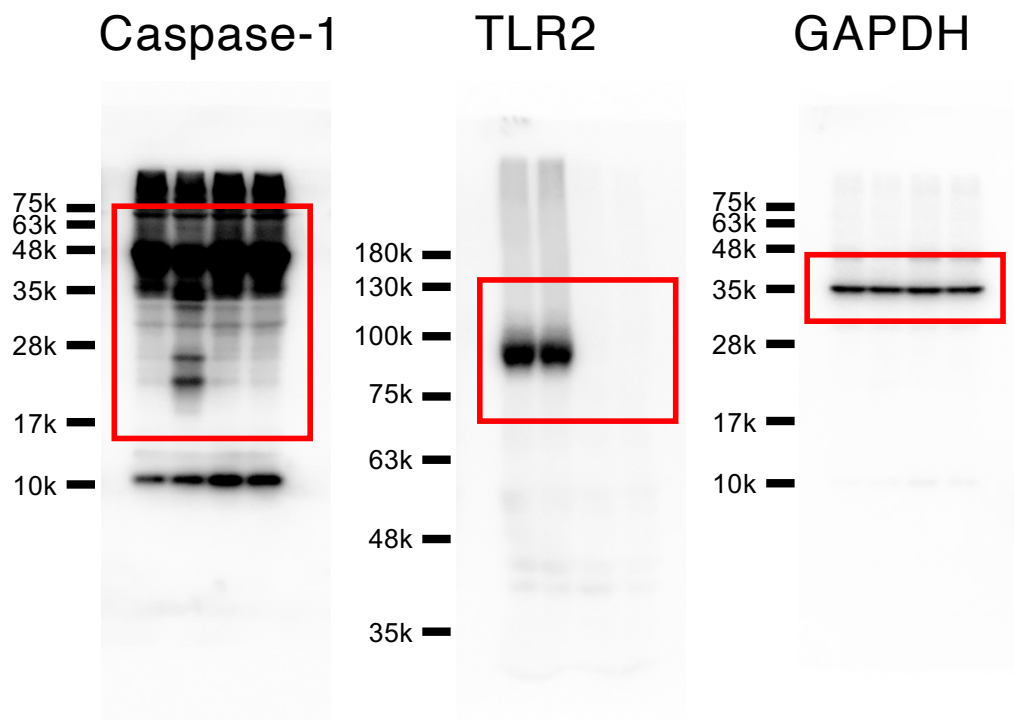

Fig. 4D

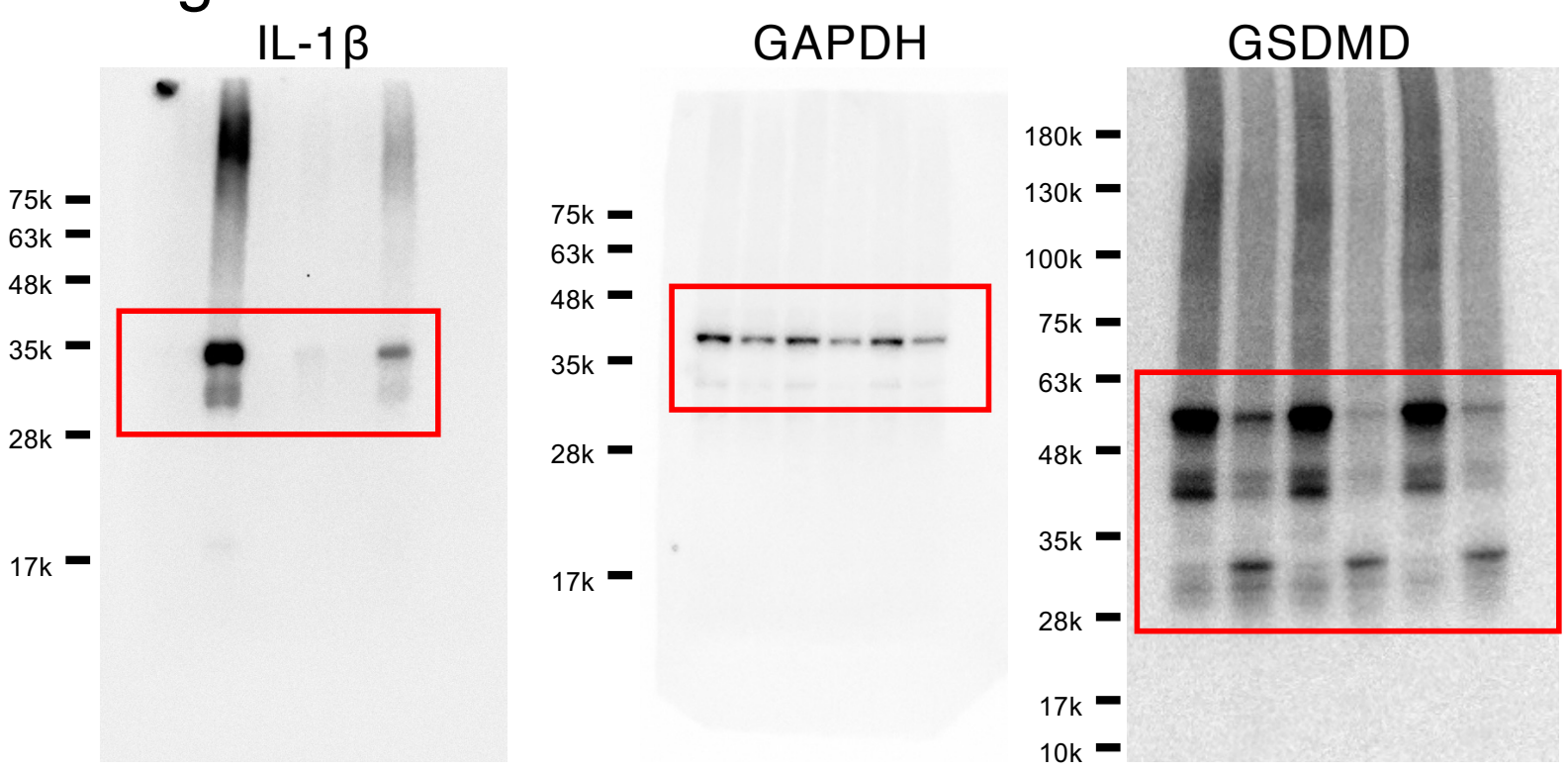

Fig. 4G

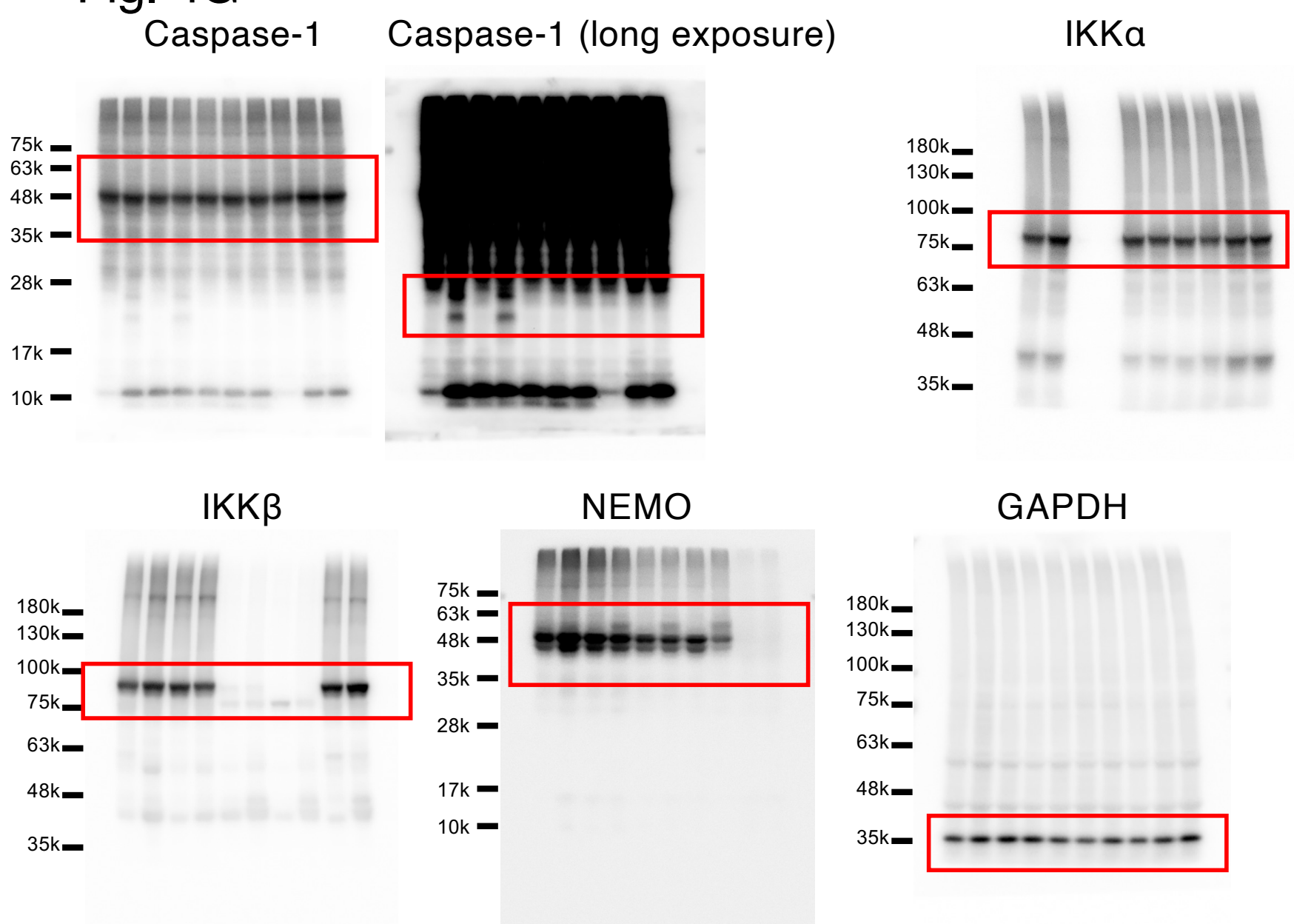

Fig. 5C

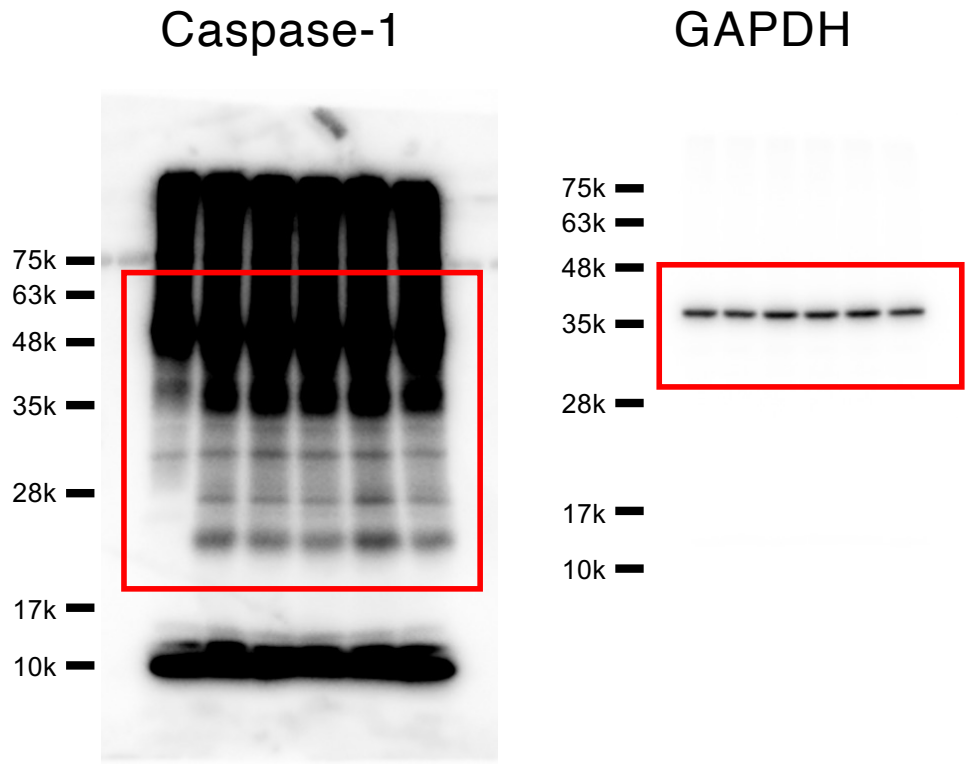

Fig. 5D

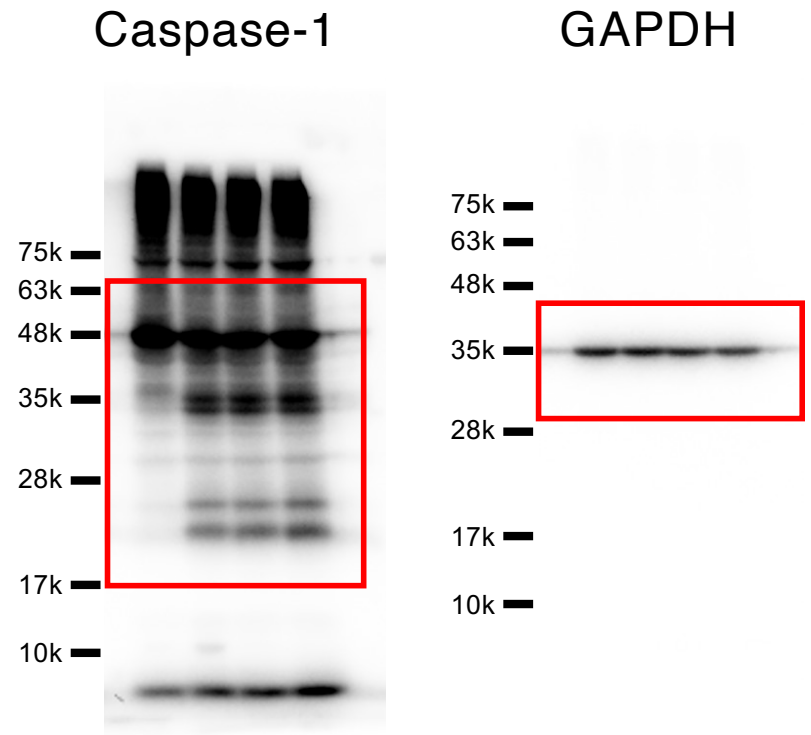

Fig. 6B

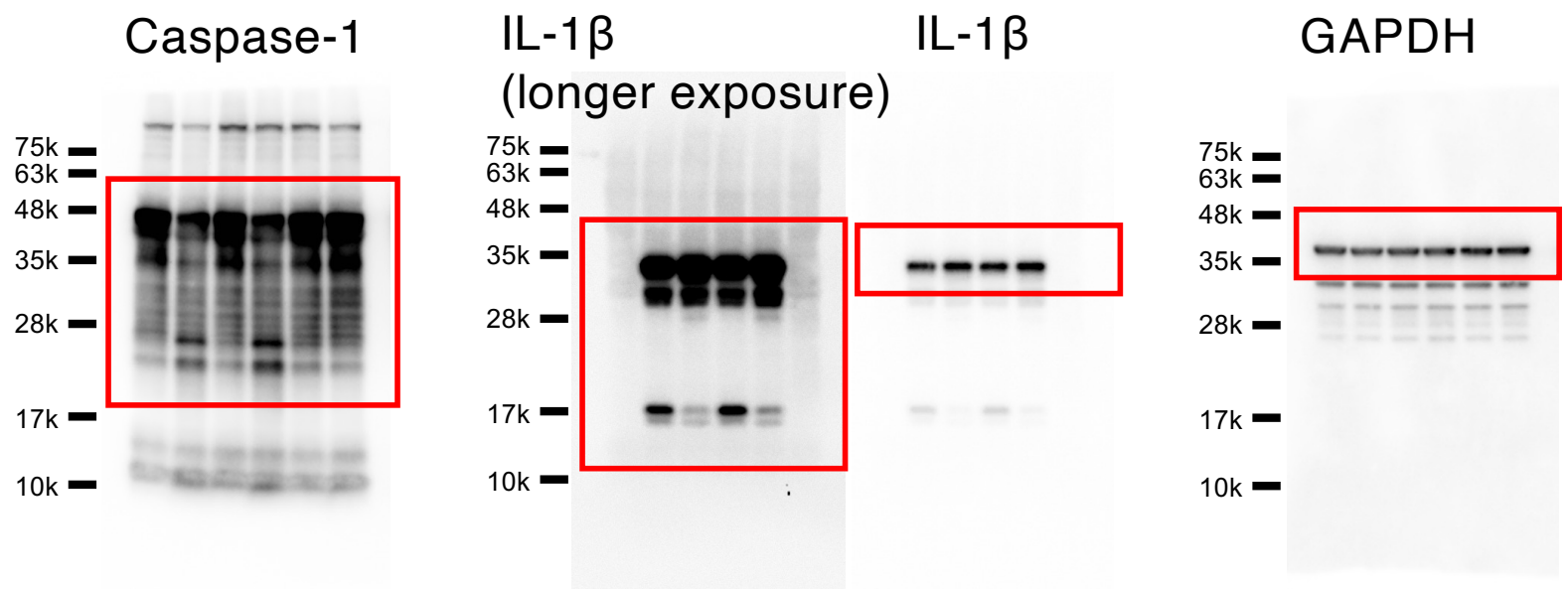

Fig. 6C

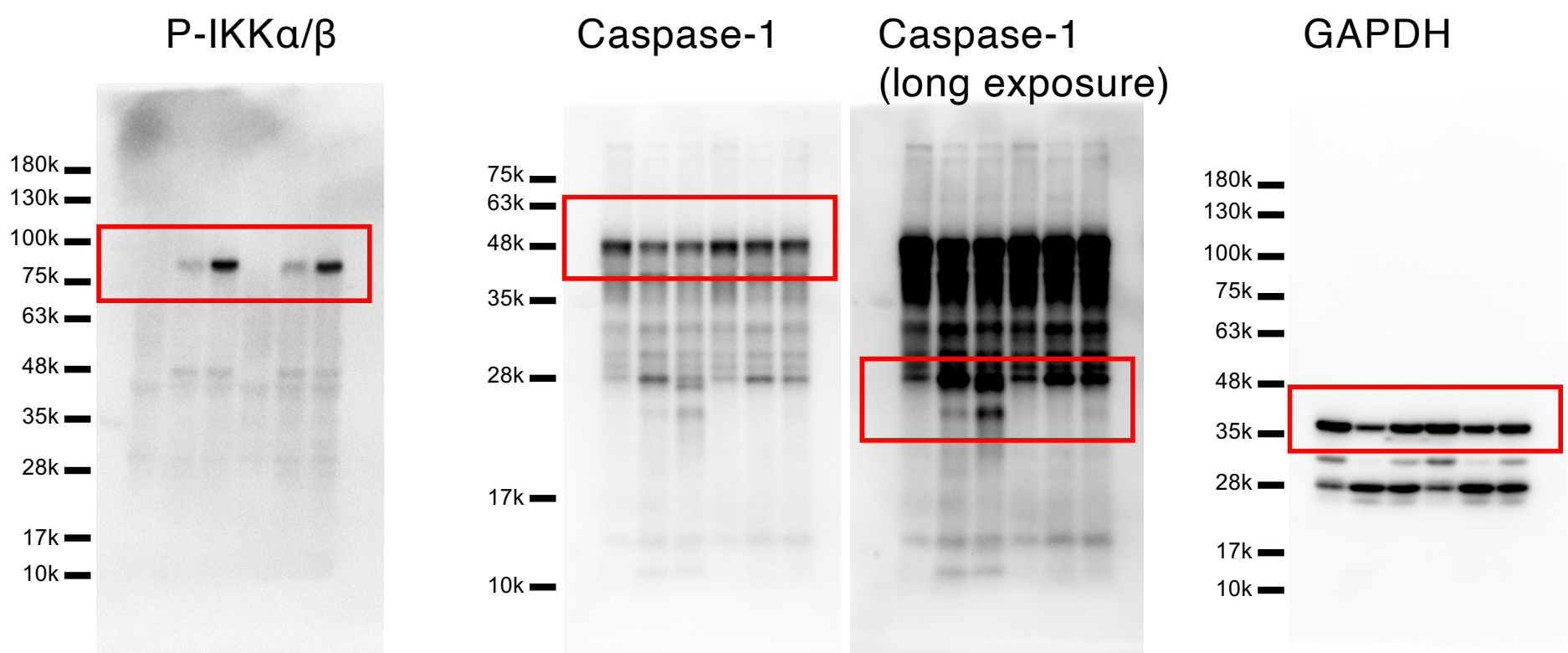

Fig. 6H

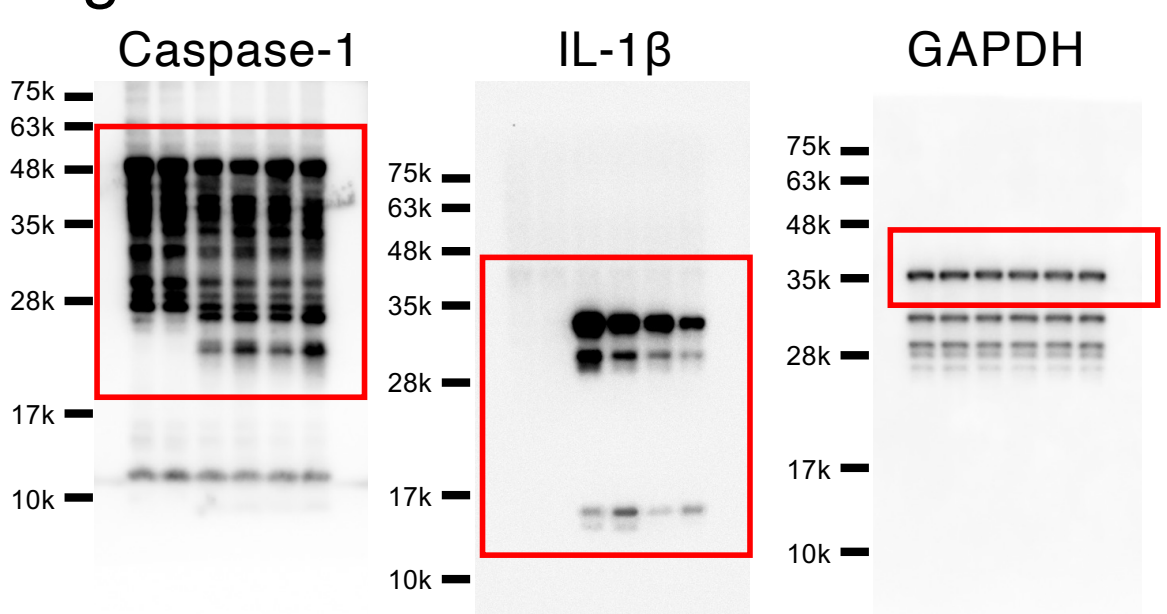

Fig. 6I

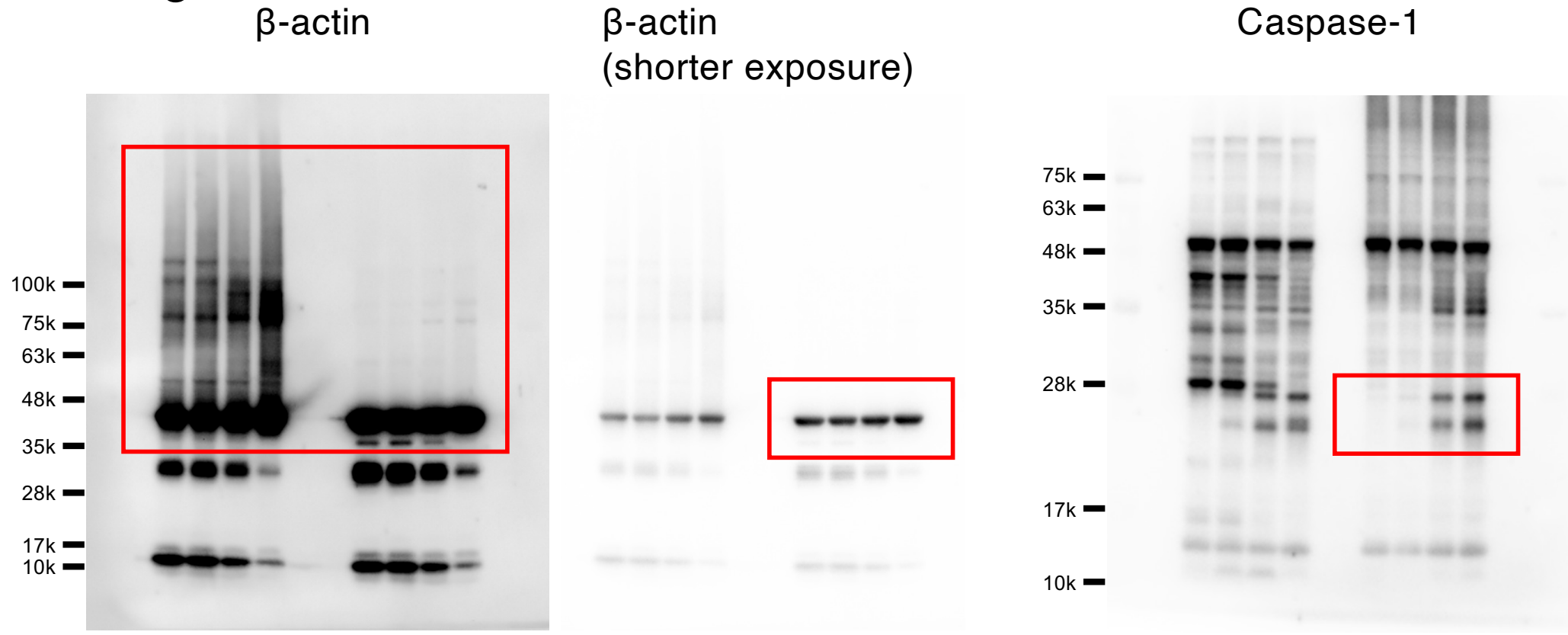

Fig. 7F

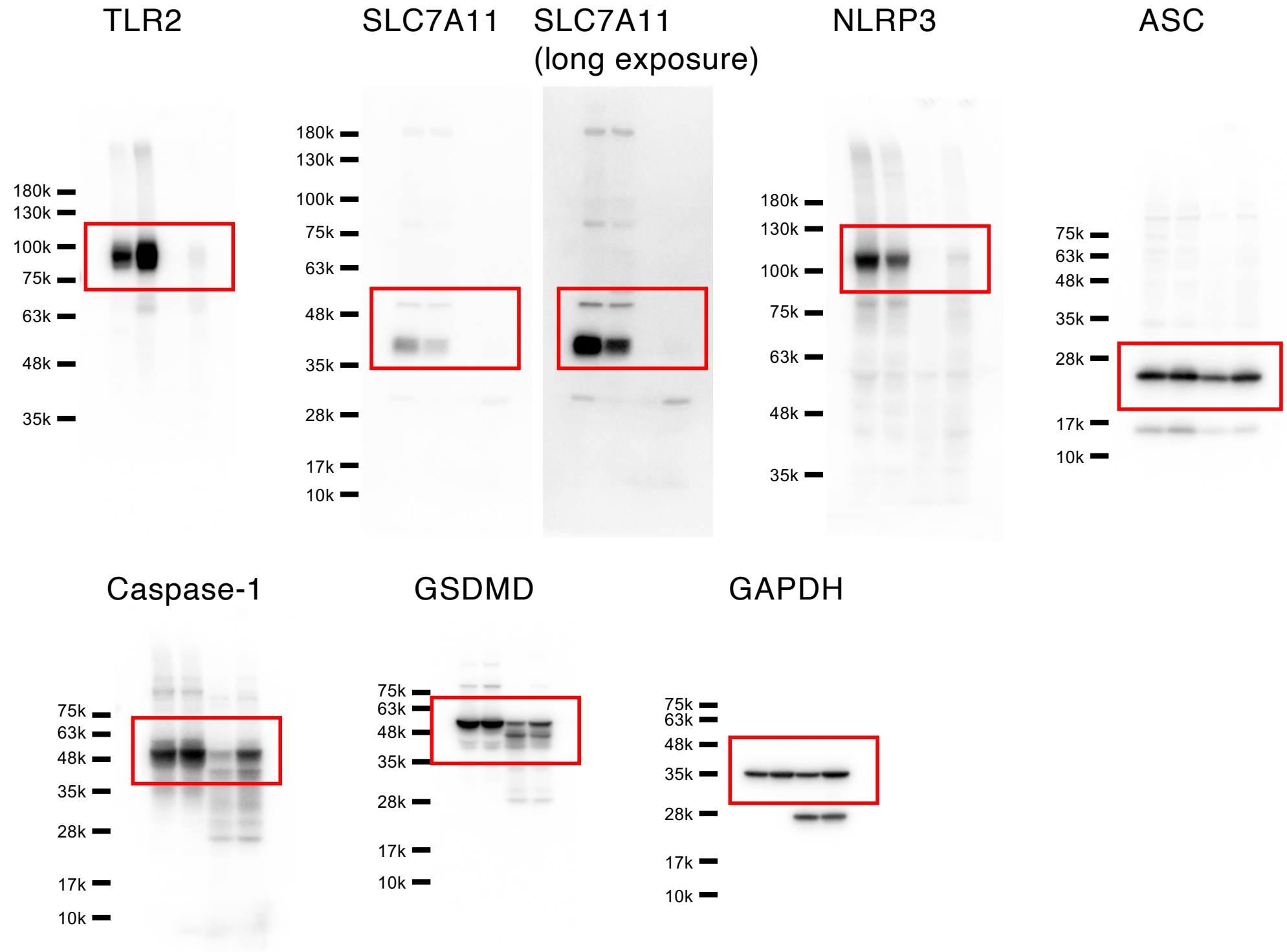

Fig. 7G

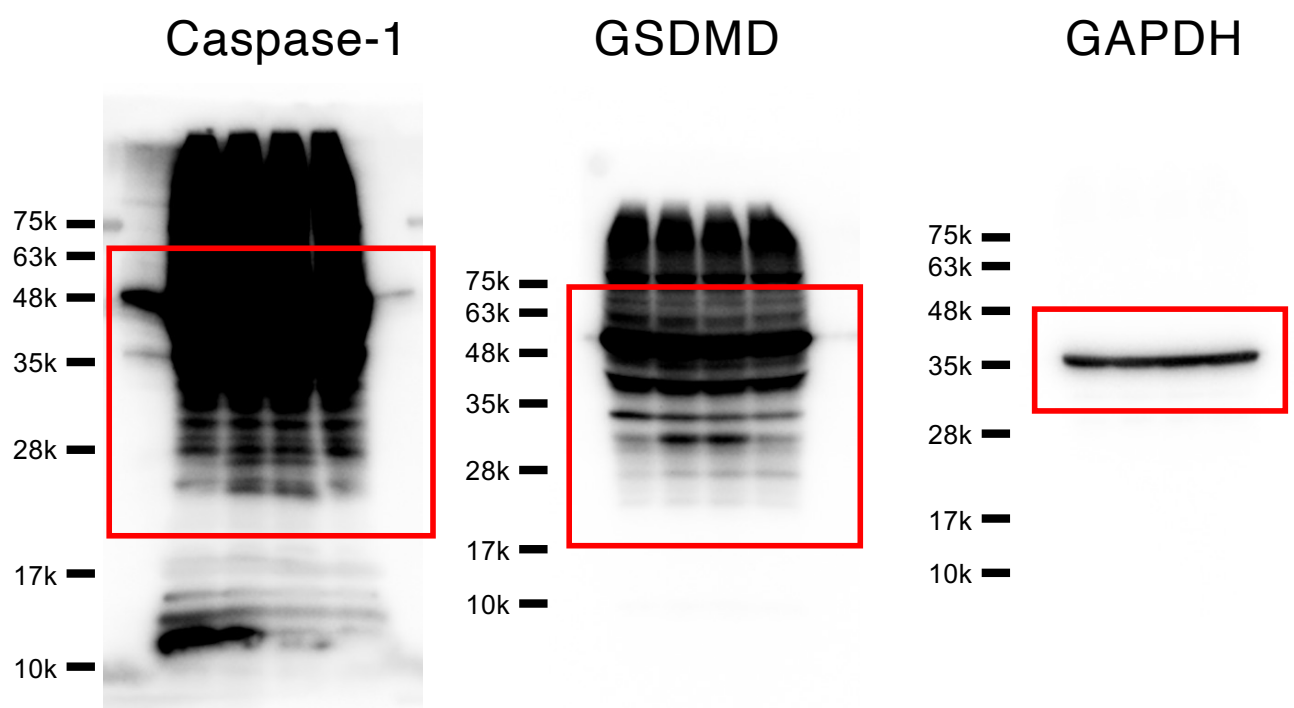

Supplementary Fig. 1C

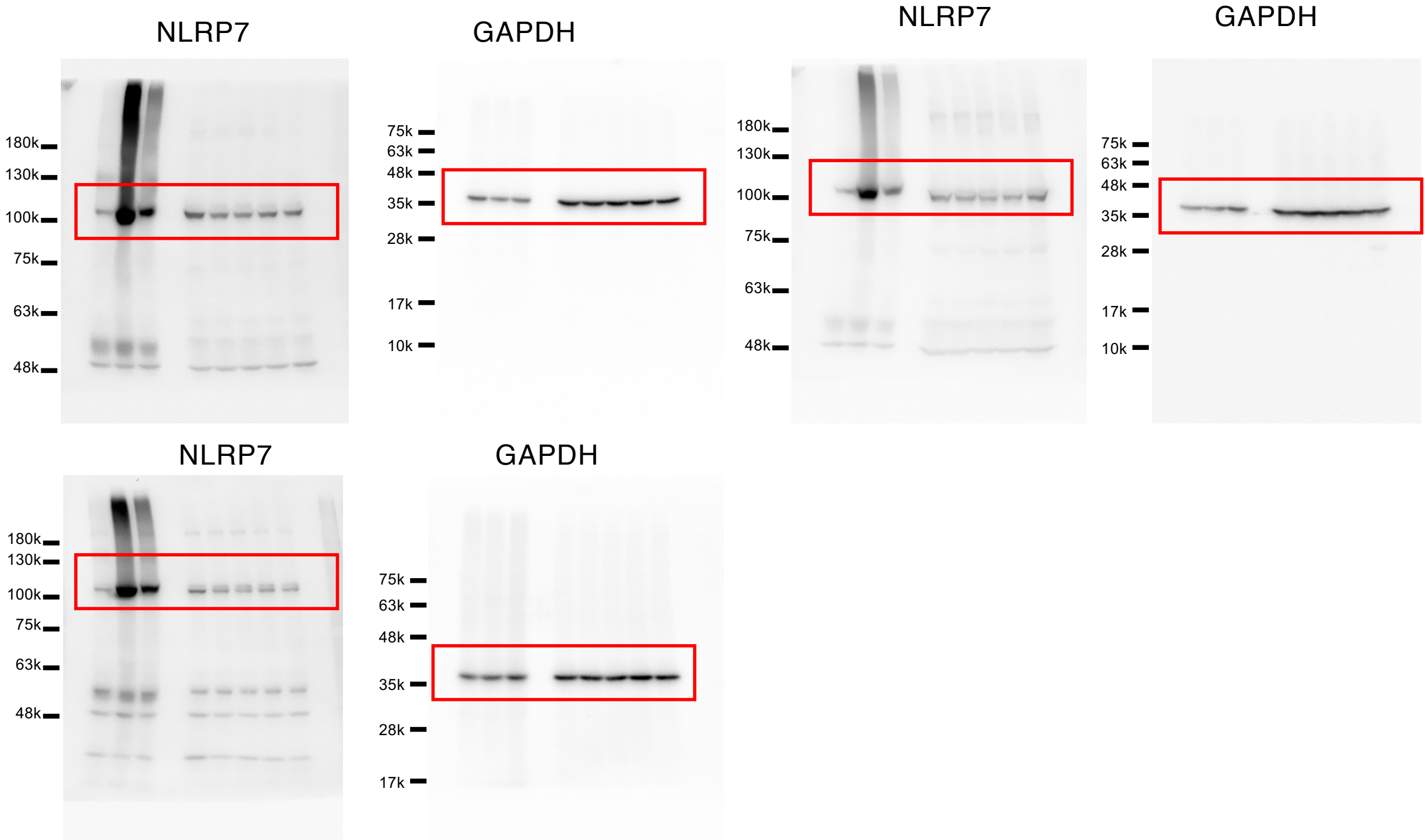

Supplementary Fig. 2A

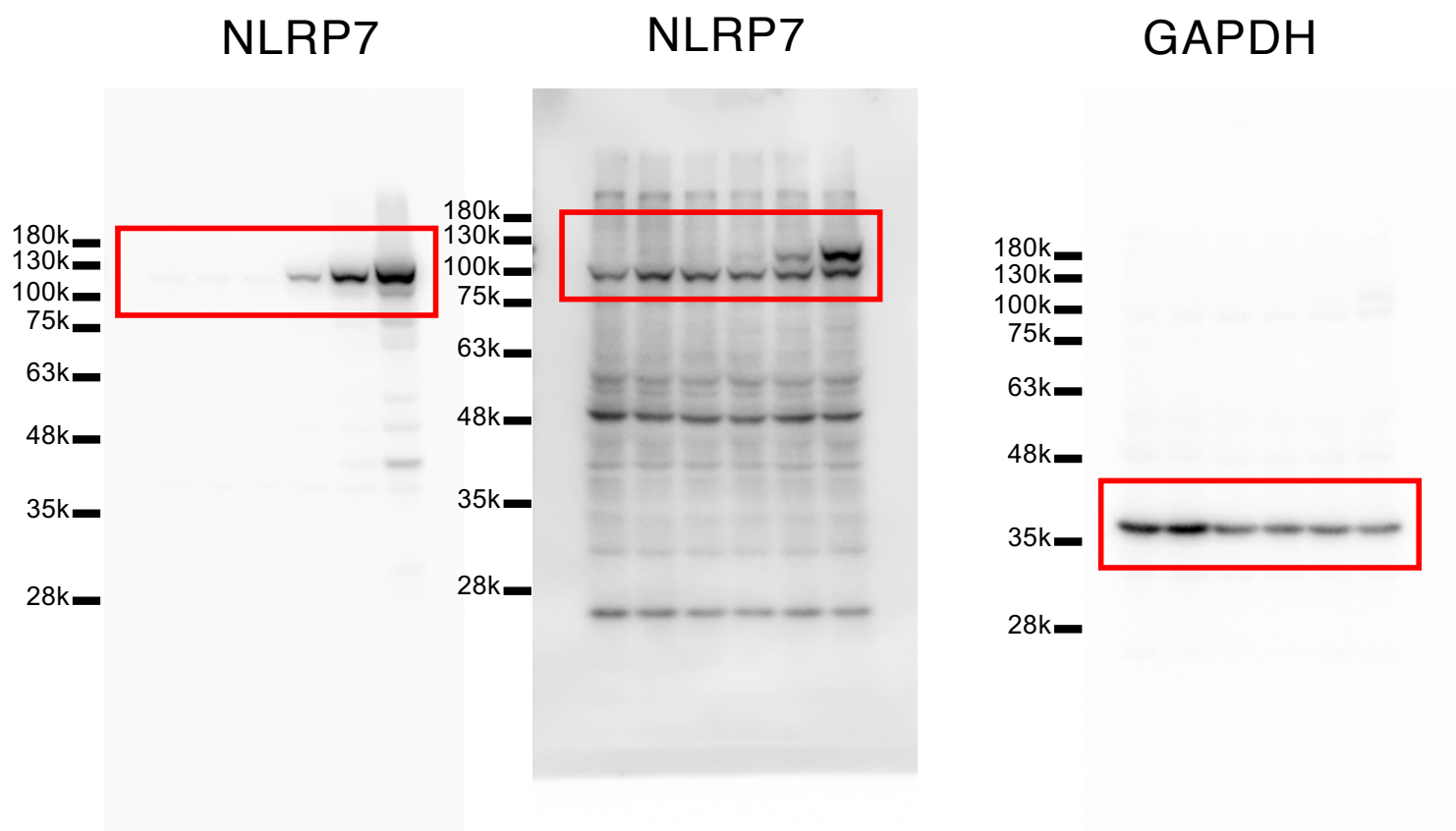

Supplementary Fig. 2D

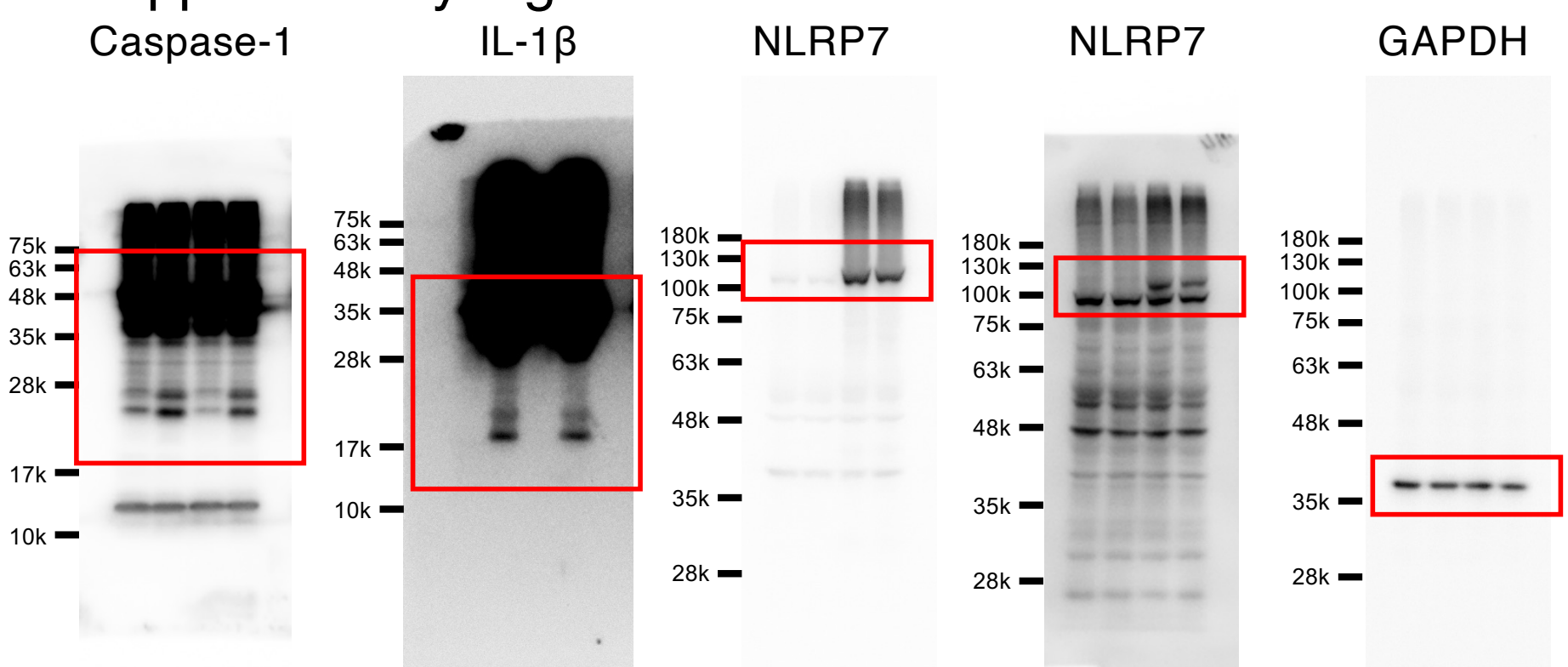

Supplementary Fig. 2E

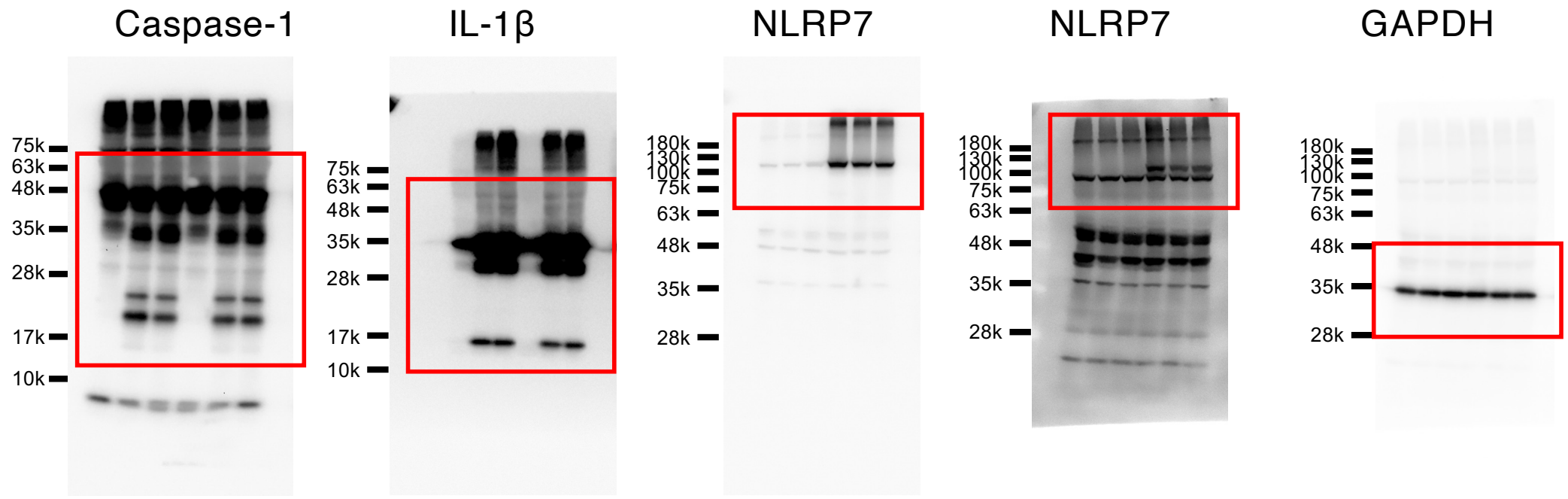

Supplementary Fig. 6B

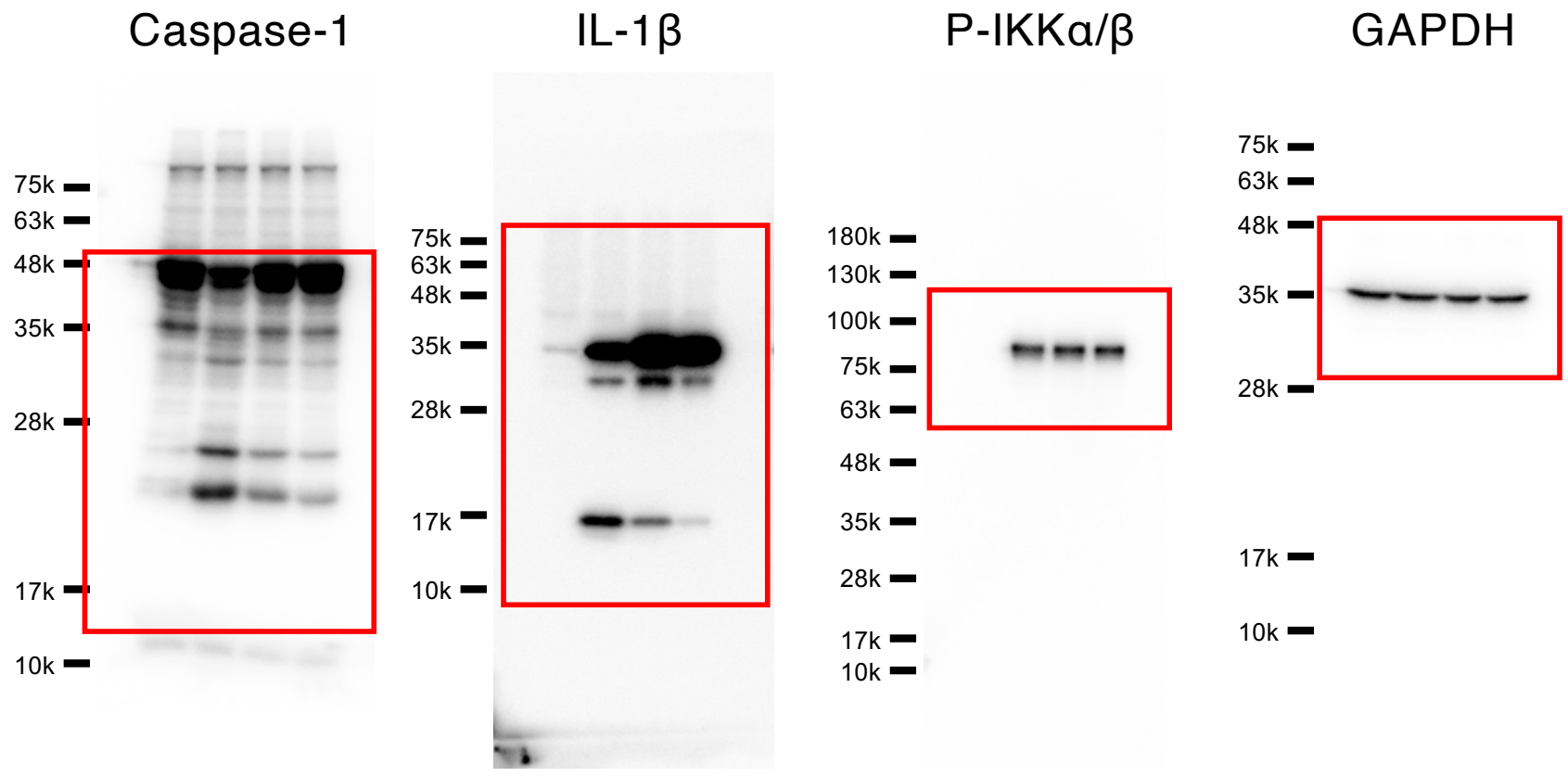

Supplementary Fig. 7A

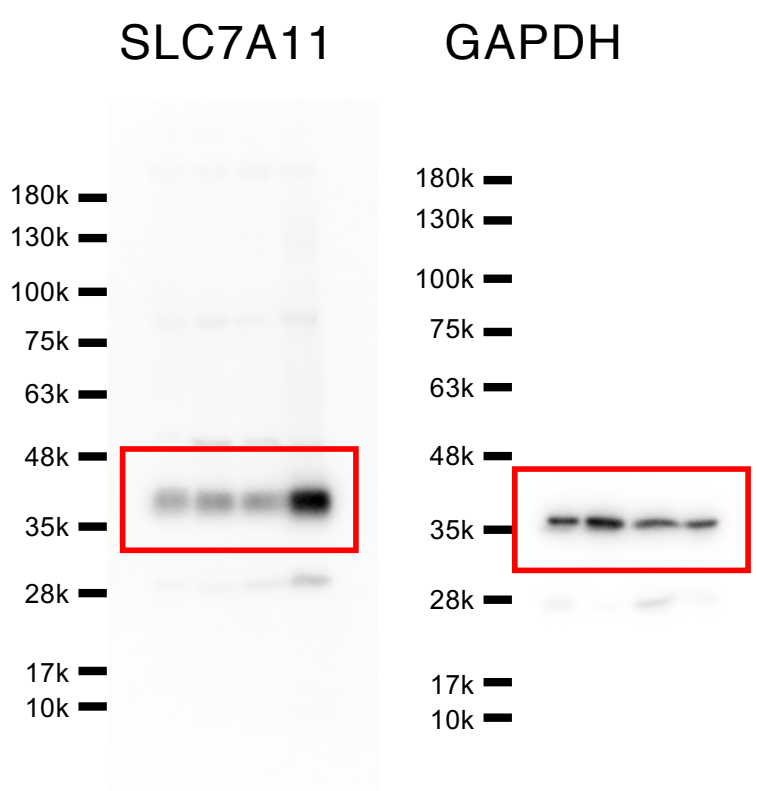

Supplementary Fig. 7B

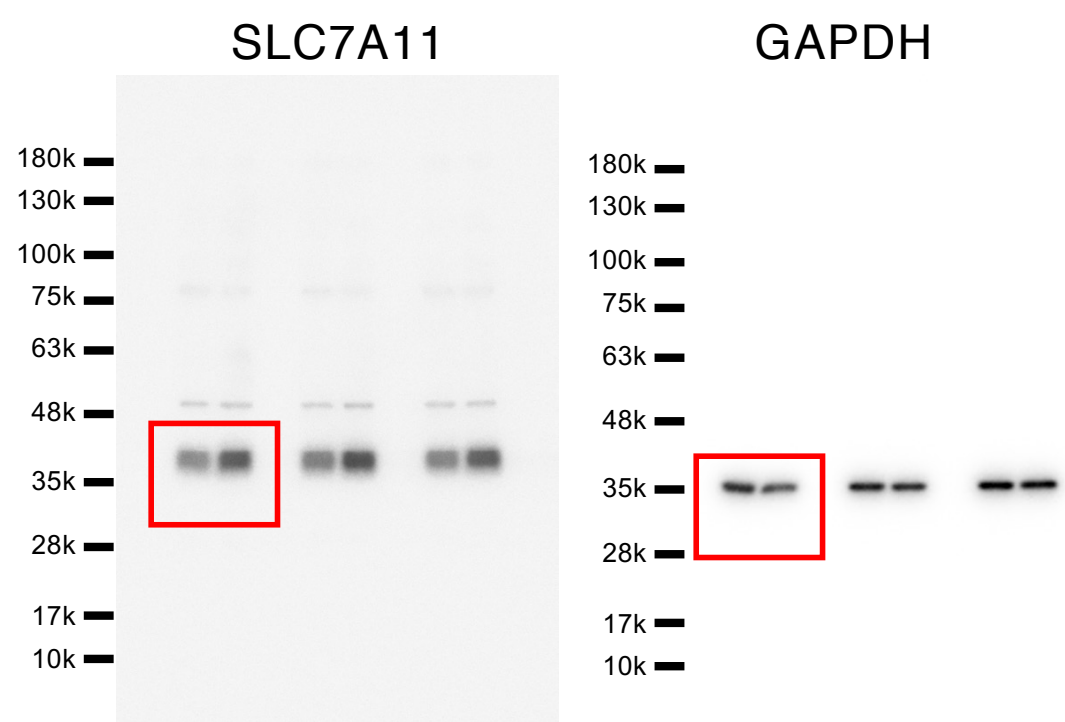

Supplementary Fig. 11B

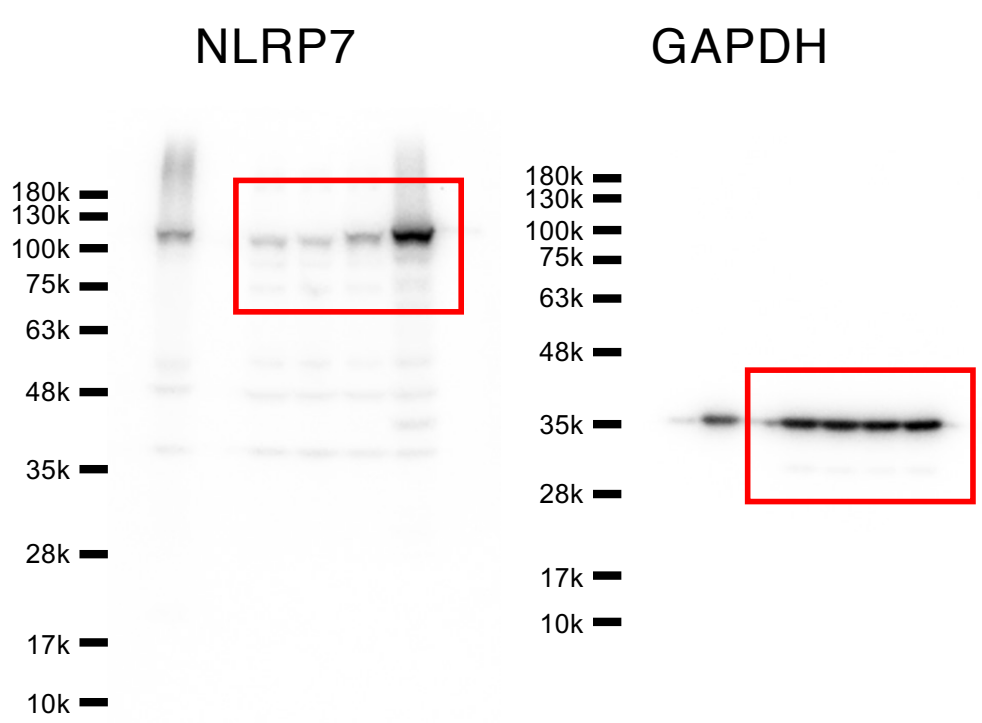

Supplement: Supplementary file 2 [file 41419_2026_9164_MOESM3_ESM.pdf]
