# Supplementary material for: Sublethal disulfide stress facilitates TLR2-dependent NLRP3 inflammasome activation and pyroptosis in leukemia cells
Source: Cell Death Dis. 2026 Aug 1;17(1):919. doi: 10.1038/s41419-026-09164-4 (PMC13720097; doi:10.1038/s41419-026-09164-4)
Supplement: Supplementary file 3 [file 41419_2026_9164_MOESM1_ESM.docx]

**Title: Sublethal Disulfide Stress Facilitates TLR2-Dependent NLRP3 Inflammasome Activation and Pyroptosis in Leukemia Cells**

**Running title: Disulfide stress in inflammasome activation**

Shoko Hosojima^1,2^, Shenghui Zhi^1,2^, Yamato Tanabe^3,4,5^, Takeshi Kinoshita^1,2^, Takayuki Hoshii^2^, Takashi Suda^1^, Kohsuke Tsuchiya^1,2,6,^*

^1^Division of Immunology and Molecular Biology, Cancer Research Institute, Kanazawa University, Kakuma-machi, Kanazawa, 920-1192, Japan.

^2^Division of Transcriptional Control and Engineering, Cancer Research Institute, Kanazawa University, Kakuma-machi, Kanazawa, 920-1192, Japan.

^3^Molecular Genetics, Graduate School of Medical Sciences, Kanazawa University, Takara-machi, Kanazawa, 920-8640, Japan.

^4^Division of Immune Environment Dynamics, Cancer Research Institute, Kanazawa University, Kakuma-machi, Kanazawa, 920-1192, Japan.

^5^Immune Network Research Unit, Pursuit of Truth Research Core, Institute for Frontier Science Initiative, Kanazawa University, Kakuma-machi, Kanazawa, 920-1192, Japan.

^6^Cancer–Immune System Interactions Unit, Cancer Research Institute, Kanazawa University, Kanazawa, Japan.

* Address correspondence to Kohsuke Tsuchiya.

E-mail address: ktsuchiya@staff.kanazawa-u.ac.jp. Phone: +81 76 264 6721.

**Supplementary Materials and Methods**

**Reagents**

Nigericin (Cayman Chemical, 11437) was purchased from Cayman Chemical. Ultrapure LPS (*Escherichia coli* O111:B4; tlrl-3pelps), Pam3CSK4 (tlrl-pms), Pam2CSK4 (tlrl-pm2s-1), and FSL-1 (tlrl-fsl) were from InvivoGen. Lipopolysaccharides from *Escherichia coli* K-235 purified by phenol extraction (L2143), KL-11743 (SML3458), RPMI1640 (R8758), and DMEM (D5796) were from Sigma-Aldrich. Opti-MEM (31985070) was from Thermo Fisher Scientific. Propidium iodide (PI, 19174-31), cystine (13003-12), dimethyl sulfoxide (09659-14), Tris(2-carboxyethyl)phosphine hydrochloride (TCEP, 07277-61), and DTT (14130-41) were from Nacalai Tesque. MCC950 (AG-CR1-3615) was from AdipoGen. 2-mercaptoethanol (131-14572), tetraethylammonium chloride (TEA, 209-16321), tetrapentylammonium chloride (TePA, 207-13681), quinine (179-00461), cycloheximide (037-20991), chloroquine diphosphate (038-17971), and phorbol 12-myristate 13-acetate (PMA, 162-23591) were from FUJIFILM Wako. Diamide (A1458) was from Tokyo Chemical Industry. z-VAD-fmk (FMK001) was from R&D Systems. Glyburide (S1716), A-740003 (S0826), probenecid (S4022), carbenoxolone sodium (S4368), RS3 (S8155), Tempol (S2910), GSK872 (S8465), necrostatin-1 (S8037), deferoxamine mesylate (S5742), and ferrostatin-1 (S7243) were from Selleck Chemicals. Doxycycline hyclate (A2951) was from BioChemica. IPG-4 AM (3021G) was from ION BIOSCIENCES.

**Plasmids**

To construct the gRNA-expressing lentiviral transfer vector, LentiGuide-Puro (Addgene #52963) was digested with FastDigest BsmBI and dephosphorylated using calf intestinal alkaline phosphatase (Takara, #2250). Oligonucleotides (Supplementary Table 1) were phosphorylated with T4 polynucleotide kinase (Takara, #2021) and ligated into the digested LentiGuide-Puro vector using T4 DNA ligase (Takara, #2011). For the construction of inducible NLRP7 expression vectors, pCW-Tet-Cas9-BFP-PGK-Blast (Addgene #196720) was digested with NheI and PacI, and NLRP7 isoform 2 cDNA (NP_996611.2), NLRP7 isoform 3 cDNA (NP_001120727.1), or BFP (control) cDNA was inserted using the In-Fusion HD Cloning Kit (Takara #639650). An inducible SLC7A11 expression vector was generated by inserting SLC7A11 cDNA (NP_055146.1) into the NheI- and PacI-digested pCW-Tet-Cas9-BFP-PGK-Blast backbone using the In-Fusion HD Cloning Kit.

**Cell culture**

THP-1 cells (ATCC TIB-202), NOMO-1 cells (Health Science Research Resources Bank, IF050474), and HL-60 cells (Health Science Research Resources Bank, JCRB0085) were grown in RPMI 1640 supplemented with 10% fetal bovine serum (FBS), 100 U/ml penicillin, and 100 μg/ml streptomycin under a humidified atmosphere with 5% CO_2_ at 37°C. We confirmed that the cell lines were free of mycoplasma contamination. These cells at high density were diluted into a tenfold volume of fresh RPMI 1640 medium, followed by incubation for 7 days to allow them to reach a near-confluent state. Before stimulation, the cells were washed with either fresh RPMI 1640 or Opti-MEM and immediately subjected to experiments. For selected experiments (Fig. 1E), THP-1 cells prepared as described above and cultured for 6 days were refreshed with new RPMI 1640 medium and incubated for an additional 24 hours prior to use. For PMA-induced differentiation, THP-1 cells were stimulated with 100 nM PMA for 2 hours, washed, and then cultured for 24 hours. The adherent cells were subsequently used for experiments. HL-60 cells were differentiated with 1% DMSO for 3 days (hereafter referred to as dHL-60 cells).

**Lentiviral manipulation of THP-1 and NOMO-1 cells**

Cas9-expressing THP-1 cells were generated as follows. Lentiviruses were produced in 293FT cells co-transfected with the lentiviral packaging vectors pMD2.G and psPAX2 (Addgene #12259 and #12260) together with pLentiCas9-T2A-BFP (Addgene #78547) using Lipofectamine 2000 (Thermo Fisher Scientific, 11668019). Viral supernatants were collected, passed through a 0.45-μm filter, and stored at −80 °C. THP-1 cells were transduced with the lentiviral particles and maintained under selection with 10 µg/ml blasticidin for 1 week. BFP-positive cells were isolated by cell sorting three times using a FACSAria Fusion cell sorter (BD Biosciences) and used as Cas9-expressing THP-1 cells. Using the same procedure, lentiviruses carrying LentiGuide-Puro encoding individual gRNAs or gRNA pools were introduced into the Cas9-expressing THP-1 cells. Transduced cells were selected with puromycin (1 µg/ml) for more than one week. Gene-knockout clones were obtained by single-cell cloning, and successful knockout was confirmed either by Nanopore sequencing of the targeted genomic locus or by assessing protein expression via Western blotting. Inducible NLRP7 expression vectors were likewise introduced into THP-1 and NOMO-1 cells via lentiviral transduction. An inducible SLC7A11 expression vector was also introduced into THP-1 cells using the same lentiviral transduction approach.

**Stimulation of cells**

Undifferentiated and PMA-differentiated THP-1 cells, NOMO-1 cells, HL-60 cells, and dHL-60 cells were stimulated with lipopeptides or nigericin for the indicated durations to induce inflammasome activation. DTT, 2-ME, and TCEP were added immediately before stimulation, diamide was added 30 minutes after stimulation, and all other inhibitors were added 30 minutes prior to stimulation. Cystine overloading was performed by incubating cells with 1 mM cystine for 20 h. For the induction of necroptosis, THP-1 cells were treated with LPS (100 ng/ml) plus z-VAD-fmk (30 µM) for 24 h. Ferroptosis was induced by incubating THP-1 cells with RSL3 (20 µM) for 24 h.

**LDH release assay**

The LDH released from cells into culture supernatants was measured using the Cytotoxicity LDH Assay Kit-WST (DOJINDO, CK12) according to the manufacturer’s protocol. The percentage of LDH release was calculated as follows: % LDH release = (sample LDH activity-background LDH)/(total LDH activity-background LDH)×100.

**ELISA**

Culture supernatants were centrifuged at 1500 × *g* for 5 min at 4 °C to remove cells and debris. The supernatants were stored at -80°C until use. Levels of IL-1β and TNF-α in the specimens were assessed by sandwich ELISAs using ELISA MAX Deluxe Set Human IL-1β (BioLegend, 437004) and ELISA MAX Standard Set Human TNF-α (BioLegend, 430201) according to the kit instructions.

**Western blotting**

For immunoblot analysis of proteins in culture supernatants, cells were cultured in Opti-MEM. Proteins in culture supernatants were precipitated with 20% trichloroacetic acid, and the pellets were washed twice with 1 ml of 80% acetone, dried, and boiled in SDS-Sample Buffer containing 20 mM DTT for 5 min. Cells were solubilized in SDS-Sample Buffer with or without DTT and boiled at 95 °C for 20 min. For detection of SLC7A11, cells were lysed in PBS containing 1% Triton X-100, 2 mM EDTA, and a protease inhibitor cocktail (Nacalai Tesque, 25955-11). Insoluble fractions were removed by centrifugation (20,000 × g, 10 min). The protein concentration of the cell lysate was measured and adjusted using a BCA assay (Pierce, 23227), after which SDS-Sample Buffer with DTT was added, and the samples were used without boiling. The samples were subjected to SDS–PAGE and transferred to PVDF membranes (Merck). Membranes were blocked with nonfat dry milk or Blocking One (Nacalai Tesque) for 1 h at room temperature and incubated overnight at 4 °C with primary antibodies diluted in Immuno-Enhancer Reagent A (Wako, 295-68614). The following primary antibodies were used: NLRP3 (Proteintech, 68102-1-Ig), NLRP7 (Proteintech, 27897-1-AP; GeneTex, GTX85156), SLC7A11 (Proteintech 26864-1-AP), GSDMD (Sigma-Aldrich, HPA044487), Caspase-1 (AdipoGen, AG-20B-0048-C100), ASC (Santa Cruz Biotechnology, sc-22514-R), IL-1β (R&D Systems, MAB201), NEK7 (Bethyl Laboratories, A302-684), IKKα (Selleck, F0995), IKKβ (Selleck, F0333), β-actin–HRP (MBL, PM053-7), GAPDH–HRP (MBL, M171-7), and the following antibodies from Cell Signaling Technology: Caspase-8 (4790), NLRC5 (72379), Pannexin-1 (91137), NEMO (2685), phospho-IKKα/β (2697), and TLR2 (12276). Secondary antibodies diluted in Immuno-Enhancer Reagent B (Wako, 292-68624) were anti-rabbit IgG HRP-linked antibody (CST, 7074; 1:1000) and anti-mouse IgG HRP-linked antibody (CST, 7076; 1:1000). Band intensities were quantified using NIH ImageJ software.

**NADPH / NADP⁺ assay**
NADPH and NADP⁺ levels were measured using the NADP/NADPH Assay Kit-WST (DOJINDO, N510) according to the manufacturer’s instructions. Briefly, GSDMD-deficient THP-1 cells (2 × 10⁶) were stimulated with Pam_3_CSK_4_ (500 ng/ml) and washed once with PBS. The cells were then lysed in 0.3 ml of NADP/NADPH Extraction Buffer. The lysates were centrifuged at 12,000 × g for 5 min, passed through a 10 kDa MWCO filtration tube, and divided into two microtubes. One aliquot was incubated at 60°C for 1 h to degrade NADP⁺ and subsequently cooled to room temperature. Samples and standards (50 µl each) were mixed with an equal volume of Assay Working Solution in a 96-well plate and incubated at 37°C for 1 h. Optical density at 450 nm was measured using a microplate reader, and total NADP⁺/NADPH and NADPH levels were quantified based on the standard curve. The NADP⁺ concentration was calculated by subtracting the NADPH level from the total NADP⁺/NADPH level.

**Quantitative RT-PCR**

Total RNA was isolated from THP-1 cells using NucleoSpin RNA (MACHEREY-NAGEL, 740955) and reverse transcribed to produce cDNA with the SuperScript VILO cDNA Synthesis Kit (Thermo Fisher Scientific, 11754050) according to the manufacturer's instructions. Quantitative RT-PCR reactions were performed using Luna Universal qPCR Master Mix (NEB, M3003) and ViiA7 Real-Time PCR System (Thermo Fisher Scientific). The primers used for quantitative RT-PCR are as listed in Supplementary Table 1. Relative expression levels of each target gene to the internal control (GAPDH) were calculated as 2^ΔCt.

**CRISPR/Cas9-based screen of TLR signaling components**

Cas9-expressing THP-1 cells lentivirally transduced with a panel of gRNAs targeting TLR signaling molecules were stimulated with 500 ng/ml Pam_3_CSK_4_ for 2 h. The cells were stained with propidium iodide (PI; 1 µg/ml; Nacalai, 19174-31), and PI-negative (live) cells were isolated by sorting on a FACSAria Fusion cell sorter. The sorted cells were grown for 1 week and then lysed in 100 mM Tris (pH 8.0) containing 0.2% SDS, 5 mM EDTA, 200 mM NaCl, and 0.1 mg/ml proteinase K, followed by overnight incubation at 50°C. Debris were removed by centrifugation at 10,000 × g for 5 min, and genomic DNA was purified using the NucleoSpin gDNA Clean-up Mini kit (MACHEREY-NAGEL, 740230). Using 60 ng of the purified genomic DNA as a template, a 843-bp region of the LentiGuide-Puro vector containing the integrated gRNA sequences was amplified by PCR (TOYOBO, KOD-201). Purified PCR products were then used as templates for qPCR to determine the relative abundance of each gRNA using primers (LentiGuide_gRNAs2 and the antisense oligonucleotide of each gRNA) and Luna Universal qPCR Master Mix (10 pg DNA per reaction). The abundance of each gRNA was calculated as the ratio of its gRNA-specific amplicon to the total gRNA pool (2^ΔCt), where the total gRNA pool was quantified by amplification of a size-matched universal region (LentiGuide_gRNAs4 and LentiGuide_gRNAa3). To assess changes after Pam_3_CSK_4_ stimulation, the abundance of each gRNA was normalized to its corresponding pre-stimulation level.

**Fluorescence microscopy**

PMA-differentiated GSDMD KO THP-1 cells were seeded onto coverslips (Fisher Scientific, 12-541-000) and cultured in Opti-MEM. Cells were stimulated with or without Pam_3_CSK_4_ (500 ng/mL) for 90 min. Following stimulation, cells were fixed by adding an equal volume of 4% paraformaldehyde phosphate buffer solution (Nacalai Tesque, 09154-56) directly to the culture medium (final concentration: 2%) and incubated for 1 h at room temperature. Cells were then washed three times with PBS and blocked/permeabilized in blocking buffer (PBS containing 10% Blocking One, 0.2% Triton X-100, and 0.1% Tween 20) for 1 h at room temperature. F-actin was stained using Phalloidin-iFluor 594 Reagent (Abcam, ab176757; 1:1000 dilution) in blocking buffer for 1 h at room temperature. After staining, cells were washed four times with PBS containing 0.1% Tween 20, followed by a final wash with distilled water. Coverslips were mounted onto glass slides using Fluoromount/Plus (Diagnostic BioSystems, K048). Images were acquired using a fluorescence microscope (Keyence, BZ-X800).

**Flowcytometry**

For detection of necrotic cells, cells were stained with PI (1 µg/mL) and analyzed using a FACS Canto II flow cytometer (BD Biosciences), with the PE channel used to detect PI fluorescence. For IPG-4 AM staining, IPG-4 AM was dissolved in DMSO containing 8% Pluronic F-127 to prepare a 1.5 mM stock solution. This solution was diluted 1:200 in HBSS (+) to generate a working solution. Near-confluent GSDMD KO THP-1 cells (8 × 10^6^ cells) were washed with HBSS (+), and cells were incubated in 4.8 mL of the working solution for 60 min at 37°C. After staining, cells were washed twice with RPMI 1640 medium. Cells were then stimulated with Pam_3_CSK_4_ (500 ng/mL) or nigericin (10 µM) in the presence or absence of DTT (0.5 mM) or 2-ME (2 mM) for the indicated times. Subsequently, cells were analyzed using FACS Canto II, with the FITC channel used to detect IPG-4 fluorescence. Data were analyzed using FlowJo V10 software.

**Preparation of primary human monocytes**

Frozen human PBMCs were purchased from Precision for Medicine (Cat# 33000-10M, Lot# 201799475 and 3010116132). Cells were thawed, washed, resuspended in RPMI 1640 medium, and used as PBMCs. The remaining PBMCs were resuspended in 100 µL of staining buffer (PBS containing 0.5% BSA and 2 mM EDTA, degassed prior to use) and stained with 5 µL of FITC-conjugated anti-human CD14 antibody (BioLegend, 367115) for 10 min at 4°C. Cells were then washed, resuspended in 90 µL of staining buffer, and incubated with 10 µL of anti-FITC MicroBeads (Miltenyi Biotec, 130-097-050) for 15 min at 4°C. After washing, cells were resuspended in 500 µL of staining buffer. CD14-positive cells were enriched using MS columns (Miltenyi Biotec, 130-042-201) and a MiniMACS Separator (Miltenyi Biotec, 130-042-102) and used as monocytes. The purity of monocytes was assessed by flow cytometry using a FACS Canto II instrument based on CD14 (FITC) staining (Supplementary Fig. 9A).

**PCR amplicon nanopore sequencing**

Genomic DNA was purified as described above. A 3,280-bp genomic region spanning exon 2 to exon 4 of the NLRP7 gene was amplified by PCR, and the resulting PCR products were purified using the QuickStep2 PCR Purification Kit (EdgeBio, 92159). The PCR products were end-prepared according to protocols of native barcoding kits (SQK-NBD-114 from Oxford Nanopore Technologies, UK, https://nanoporetech.com/ja/document/ligation-sequencing-amplicons-native-barcoding-v14-sqk-nbd114-24). The amplicons were purified using AMPure XP beads (Beckman Coulter, A63881). The purified amplicons were barcoded using the native ONT barcoding kits SQK-NBD114, according to manufacturer’s instructions. The barcoded amplicon DNAs were pooled in equal concentrations and purified using AMPure XP beads. In the final step, adapter ligation was carried out according to SQK-NBD114 kit. PCR amplicon libraries were sequenced in a run of the PromethION 2 Solo device with MinKNOW software v.24.11.10 (Oxford Nanopore Technologies). ONT pod5 files were first converted to unaligned bam using dorado v.0.9.5 (https://github.com/nanoporetech/dorado) setting with sup@v5.0.0 --kit-name SQK-NBD114-24. Sequencing data were evaluated using NanoPlot (https://github.com/wdecoster/NanoPlot) and then trimmed for adapter using Porechop (https://github.com/rrwick/Porechop). Low-quality reads were removed using chopper (https://github.com/wdecoster/chopper) setting with -q 10 -l 100. To obtain consensus sequences, reads were assembled using Flye v2.9.5 (https://github.com/mikolmogorov/Flye/tree/flye), with following settings: --nano-hq -g 3000.

**Statistics**

Sample size was determined according to our experience and literature reporting similar experiments. Normality and variance were not formally tested due to the limited sample size. Statistical analyses were performed using an unpaired two-tailed t-test with Welch’s correction or one-way or two-way ANOVA followed by Dunnett’s multiple comparisons test or Tukey's multiple comparisons test, depending on the number of groups being compared, the number of independent variables, and whether comparisons were made against a single control group or among all groups. GraphPad Prism software ver. 9 was used for the statistical analyses. A *p*-value less than 0.05 was considered to be statistically significant.

**Supplementary Figure Legends**

**Supplementary Fig. 1. THP-1 cells do not express NLRP7 at substantial levels. A** Genomic sequences within the NLRP7 locus of each NLRP7 KO THP-1 clone (NLRP7 KO^1^, KO^2^, and KO^3^). The gRNA target sequences are highlighted in red. Dashes indicate deletions, and nucleotide substitutions are shown in blue. **B** Quantitative RT-PCR analysis of NLRP3 mRNA expression in WT THP-1 cells stimulated with or without LPS (100 ng/ml) for 24 h or in THP-1 cells transduced with DOX-inducible NLRP7 isoform 2 and treated with or without DOX (100 ng/ml) for 24 h. **C** Immunoblot detection of NLRP7 protein in cell lysates from WT THP-1 cells and control and NLRP7 KO THP-1 clones. Cell lysates from THP-1 cells expressing DOX-inducible NLRP7 (isoforms 2 and 3) were used as positive controls. The asterisks (*) indicate nonspecific bands. These bands were detected at comparable levels in control and *NLRP7*-disrupted cells, suggesting that they represent nonspecific signals rather than endogenous NLRP7. The graphs depict the mean ± SD of triplicate cultures (B). Data are from one representative of (B) or from (C) three biologically independent experiments with similar results.

**Supplementary Fig. 2. Overexpression of NLRP7 does not enhance BLP-induced inflammasome activation. A-D** Control (DOX-inducible BFP) and DOX-inducible NLRP7 isoform 2-transduced THP-1 cells were treated with DOX for 24 h (A-D) and then stimulated with FSL-1 or Pam3 (500 ng/ml) for 6 h (B-D). Immunoblot analysis of NLRP7 (A, D), caspase-1, and IL-1β (D) in cell lysates (A) or S+L (D). LDH release (B) and IL-1β levels in culture supernatants (C) were measured. **E** PMA-differentiated THP-1 cells harboring DOX-inducible NLRP7 isoform 2 or control were treated with DOX (1 μg/ml) for 24 h and then stimulated as in A. Immunoblot analysis in S+L. The asterisks (*) indicate nonspecific bands. The graphs depict the mean ± SD of triplicate cultures (B, C). Data are from one representative of three biologically independent experiments with similar results.

**Supplementary Fig. 3. BLP-induced pyroptosis depends on NLRP3. A** Control and NLRP3 KO THP-1 cells (undifferentiated, near-confluent) were stimulated with FSL-1 or Pam3 (500 ng/ml) for 24 h. **B, C** THP-1 cells harboring DOX-inducible NLRP7 isoform 3 or control were treated with DOX (1 μg/ml) for 24 h, pretreated with or without MCC950 (10 μM) for 30 min, and then stimulated as in A for 24 h. LDH release assay was performed (A, B). IL-1β levels in culture supernatants (C). The graph depicts the mean ± SD of triplicate cultures. Data are from one representative of three biologically independent experiments with similar results. P values were determined using two-way ANOVA followed by Dunnett's multiple comparisons test (A). P values are shown only for comparisons of interest, including between the indicated groups or versus the respective control groups. *P < 0.05; **P < 0.01; ***P < 0.001; n.s., not significant.

**Supplementary Fig. 4. Screening for TLR2 signaling components involved in pyroptosis induction by Pam_3_CSK_4_.** A gene-edited THP-1 cell population was generated by introducing a panel of gRNAs targeting TLR signaling components into Cas9-expressing THP-1 cells. The cells were stimulated with Pam3 (500 ng/ml) for 6 h, and surviving cells were isolated. Changes in the relative abundance of each gRNA before and after stimulation were quantified and expressed as fold changes (post-/pre-stimulation). The data correspond to those shown in the heat map in Fig. 4F and are presented here as a floating bar chart. The line within each bar indicates the median. Data are pooled from three biologically independent experiments.

**Supplementary Fig. 5. Validation of necroptosis and ferroptosis inhibitors used in this study. A** THP-1 cells were pretreated with the necroptosis inhibitors GSK872 (40 μM) or necrostatin-1 (Nec-1, 40 μM), or with DMSO as a control, for 30 min, and then treated with LPS (100 ng/ml) plus z-VAD-fmk (30 μM) for 24 h to induce necroptosis. **B** THP-1 cells were pretreated with the ferroptosis inhibitors DFO (100 μM) or ferrostatin-1 (Ferr-1, 20 μM), or with DMSO as a control, for 30 min, and then treated with RSL3 (20 μM) for 24 h to induce ferroptosis. Cell lysis was monitored by LDH release assay (A, B). The graphs depict the mean ± SD of duplicate (B) or triplicate (A) cultures. Data are from one representative of two biologically independent experiments with similar results. P values were determined using one-way (A) or two-way (B) ANOVA followed by Dunnett's multiple comparisons test. P values are shown only for comparisons of interest, including between the indicated groups or versus the respective control groups. *P < 0.05; **P < 0.01; ***P < 0.001; n.s., not significant.

**Supplementary Fig. 6. Effects of reducing agents on BLP- or nigericin-induced NLRP3 activation. A-D** THP-1 cells (A), PMA-differentiated THP-1 cells (C, D), or GSDMD KO THP-1 cells (B) were stimulated with 500 ng/mL of Pam3 (A-C) or 10 μM of nigericin (D) for 60 min (B), 90 min (A, D) or 3 h (C) in the presence or absence of the indicated concentrations of reducing agents. LDH release assay was performed (A-C). Immunoblot analysis of caspase-1, IL-1β, and phosphorylated IKKα/β in cell lysates (B). The graphs depict the mean ± SD of triplicate cultures. Data are from one representative of three biologically independent experiments with similar results. P values were determined using two-way ANOVA followed by Dunnett's multiple comparisons test (A, D) or Tukey's multiple comparisons test (C). P values are shown only for comparisons of interest, including between the indicated groups or versus the respective control groups. *P < 0.05; **P < 0.01; ***P < 0.001; n.s., not significant.

**Supplementary Fig. 7. NLRP3 is essentially required for the enhancement of Pam_3_CSK_4_-induced pyroptosis by diamide.** **A** Immunoblot analysis of SLC7A11 in cell lysates from control (DOX-inducible BFP) and DOX-inducible SLC7A11-transduced THP-1 cells treated with or without DOX for 24 h. **B** Immunoblot analysis of SLC7A11 in THP-1 cells cultured in fresh medium for 1 day (Re-cultured) or 7 days (Near-confluent). Band intensity was quantified (right panel). **C** Control and NLRP3 KO THP-1 cells were stimulated with Pam3 (500 ng/ml) for 3 h. Diamide was added to the cultures 30 min after stimulation. LDH release assay was performed. **D** Fluorescence microscopy images of F-actin staining in PMA-differentiated GSDMD KO THP-1 cells stimulated with or without Pam3 for 90 min. Scale bars, 20 μm. The graphs depict the mean ± SD of triplicate cultures (B, C). Data are from one representative of three biologically independent experiments with similar results. P values were determined using an unpaired t test with Welch’s correction (B, C). P values are shown only for comparisons of interest, including between the indicated groups or versus the respective control groups. *P < 0.05; **P < 0.01; ***P < 0.001; n.s., not significant.

**Supplementary Fig. 8. Disulfide stress-dependent K^+^ efflux upon TLR2 stimulation. A, B** GSDMD KO THP-1 cells were stained with or without IPG-4 AM and stimulated with or without nigericin (10 μM) for 20 min (A) or Pam3 (500 ng/ml) for 90 min (B). Left panel and right panel show gating strategy and representative histograms of IPG-4 fluorescence, respectively (A). Representative histograms of IPG-4 fluorescence in cells stimulated with or without Pam3 in the presence or absence of DTT (0.5 mM) or 2-ME (2 mM) (B). The same unstimulated control histogram is overlaid in each panel to allow direct comparison with each treatment condition (B). Data are from one of three independent experiments with similar results.

**Supplementary Fig. 9. Responses of PBMCs to TLR2 stimulation. A** Flow cytometric analysis of PBMCs and enriched monocytes derived from individual donors. Upper panels: forward scatter versus side scatter dot plots with gating strategy. Lower panels: histograms of CD14-FITC staining. **B-D** PBMCs were stimulated with Pam3 (500 ng/mL) for 6 h or left unstimulated. Alternatively, nigericin (10 μM) was added after 4 h of Pam3 stimulation, followed by an additional 2 h incubation. LDH release was measured (B), and IL-1β (C) and TNF-α (D) levels in culture supernatants were quantified by ELISA. The graphs depict the mean ± SD of triplicate cultures (B-D). P values were determined using one-way ANOVA followed by Dunnett's multiple comparisons test (B-D). P values are shown only for comparisons of interest, including between the indicated groups or versus the respective control groups. *P < 0.05; **P < 0.01; ***P < 0.001; n.s., not significant.

**Supplementary Fig. 10. Differential induction of pyroptosis among leukemia cell lines following TLR2 stimulation. A, B** THP-1, NOMO-1, and dHL-60 cells were stimulated with FSL-1 or Pam3CSK4 (500 ng/mL) or left unstimulated for 4 h. In some experiments, NOMO-1 cells were pretreated with MCC950 (10 μM) for 30 min or stimulated in the presence of 2-ME (2 mM) (B). Representative histograms of PI fluorescence in PI-stained cells analyzed by flow cytometry are shown (A, B). **C** Gating strategy. Data are representative of three independent biological experiments with similar results.

**Supplementary Fig. 11. NLRP7 does not promote BLP-induced pyroptosis in NOMO-1 cells. A** Quantitative RT-PCR analysis of NLRP7 mRNA in NOMO-1 cells stimulated with or without LPS (100 ng/ml) for 24 h, alongside THP-1 cells expressing DOX-inducible NLRP7 isoform 2 as a positive control. **B** DOX-inducible BFP (as a control) or NLRP7 isoform 3-transduced NOMO-1 cells were treated with DOX for 24 h, and NLRP7 protein levels were analyzed by immunoblotting. **C** Cells shown in B were pretreated with or without MCC950 (10 μM) for 30 min and then stimulated with Pam3 (500 ng/mL) or left unstimulated for 4 h. LDH release assay was performed. The graph depicts the mean ± SD of triplicate cultures. Data are from one of three independent experiments with similar results. P values were determined using one-way ANOVA followed by Dunnett's multiple comparisons test (A). P values are shown only for comparisons of interest, including between the indicated groups or versus the respective control groups. *P < 0.05; **P < 0.01; ***P < 0.001; n.s., not significant.

**Supplementary Fig. 12. Schematic model proposed in this study.** Under high-density culture conditions in monocytic leukemia cells, TLR2 stimulation by BLPs induces disulfide stress, which promotes K^+^ efflux probably through transient plasma membrane damage, thereby leading to NLRP3 inflammasome-dependent pyroptosis.

**Supplementary Table 1: Oligonucleotides used in this study**

| **Application** | **Oligo names** | **Sequences** |
| --- | --- | --- |
| gRNA | hASC_CRISPR_3S | CAC CGG TGA GGT CCA AGG CGT CCA |
| gRNA | hASC_CRISPR_3A | AAA CTG GAC GCC TTG GAC CTC ACC |
| gRNA | hCASP1_CRISPR_4S | CACCGCATTGAGTTGTAGTATATC |
| gRNA | hCASP1_CRISPR_4A | AAACGATATACTACAACTCAATGC |
| gRNA | hGSDMD_CRISPR_1S | CACCGCTTCCACTTCTACGATGCCA |
| gRNA | hGSDMD_CRISPR_1A | AAACTGGCATCGTAGAAGTGGAAGC |
| gRNA | hNLRP7_CRISPR_1S | CAC CGA AGA CCC CAT GGT CTG AGG |
| gRNA | hNLRP7_CRISPR_1A | AAA CCC TCA GAC CAT GGG GTC TTC |
| gRNA | hNLRP7_CRISPR_4S | CAC CGT CAA TGT CTC CTT GCC AAA |
| gRNA | hNLRP7_CRISPR_4A | AAA CTT TGG CAA GGA GAC ATT GAC |
| gRNA | hCASP8_CRISPR_1S | CAC CGC TCT TCC GAA TTA ATA GAC |
| gRNA | hCASP8_CRISPR_1A | AAA CGT CTA TTA ATT CGG AAG AGC |
| gRNA | hNLRC5_CRISPR_1S | CAC CGG CCC CCT CCG GAG TGT CTA |
| gRNA | hNLRC5_CRISPR_1A | AAA CTA GAC ACT CCG GAG GGG GCC |
| gRNA | hNLRP3_CRISPR_1S | CAC CGG CTG CAT CCC CCT CCC GAG |
| gRNA | hNLRP3_CRISPR_1A | AAA CCT CGG GAG GGG GAT GCA GCC |
| gRNA | hNLRP3_CRISPR_2S | CAC CGG ATC TTC GCT GCG ATC AAC |
| gRNA | hNLRP3_CRISPR_2A | AAA CGT TGA TCG CAG CGA AGA TCC |
| gRNA | hNEK7_CRISPR_1S | CAC CGA AGG CCT TAC GAC CGG ATA |
| gRNA | hNEK7_CRISPR_1A | AAA CTA TCC GGT CGT AAG GCC TTC |
| gRNA | hPANX1_CRISPR_1S | CAC CGC TGC GAA ACG CCA GAA CAG |
| gRNA | hPANX1_CRISPR_1A | AAA CCT GTT CTG GCG TTT CGC AGC |
| gRNA | hTLR2_CRISPR_1S | CAC CGC CTT GAC CTG TCC AAC AAC |
| gRNA & qPCR | hTLR2_CRISPR_1A | AAA CGT TGT TGG ACA GGT CAA GGC |
| gRNA | hTLR2_CRISPR_2S | CAC CGT GGA AAC GTT AAC AAT CCG G |
| gRNA & qPCR | hTLR2_CRISPR_2A | AAA CCC GGA TTG TTA ACG TTT CCA C |
| gRNA | hMyD88_CRISPR_1S | CAC CGT ACT TGG AGA TCC GGC AAC |
| gRNA & qPCR | hMyD88_CRISPR_1A | AAA CGT TGC CGG ATC TCC AAG TAC |
| gRNA | hMyD88_CRISPR_2S | CAC CGG ACA CAG GTG GCG GCC GAC |
| gRNA & qPCR | hMyD88_CRISPR_2A | AAA CGT CGG CCG CCA CCT GTG TCC |
| gRNA | hTIRAP_CRISPR_1S | CAC CGG AGG TGC TTT CTG GGG AGT |
| gRNA & qPCR | hTIRAP_CRISPR_1A | AAA CAC TCC CCA GAA AGC ACC TCC |
| gRNA | hTIRAP_CRISPR_2S | CAC CGG GTA GTG GGC TGT CCT GTG |
| gRNA & qPCR | hTIRAP_CRISPR_2A | AAA CCA CAG GAC AGC CCA CTA CCC |
| gRNA | hIRAK1_CRISPR_1S | CAC CGC GGG CGT TGC GGT TGA TCC A |
| gRNA & qPCR | hIRAK1_CRISPR_1A | AAA CTG GAT CAA CCG CAA CGC CCG C |
| gRNA | hIRAK1_CRISPR_2S | CAC CGT ACA AGA AGT GCT GGG CGC |
| gRNA & qPCR | hIRAK1_CRISPR_2A | AAA CGC GCC CAG CAC TTC TTG TAC |
| gRNA | hIRAK2_CRISPR_1S | CAC CGT CCA CAA GTT GCT GGA CGG |
| gRNA & qPCR | hIRAK2_CRISPR_1A | AAA CCC GTC CAG CAA CTT GTG GAC |
| gRNA | hIRAK2_CRISPR_2S | CAC CGG TCA GGT CTG TGA TCA CGT |
| gRNA & qPCR | hIRAK2_CRISPR_2A | AAA CAC GTG ATC ACA GAC CTG ACC |
| gRNA | hIRAK4_CRISPR_1S | CAC CGG CAC CAC AAA TTG CAC AGT |
| gRNA & qPCR | hIRAK4_CRISPR_1A | AAA CAC TGT GCA ATT TGT GGT GCC |
| gRNA | hIRAK4_CRISPR_2S | CAC CGT CAA ACA GTA ATT CAG AAG |
| gRNA & qPCR | hIRAK4_CRISPR_2A | AAA CCT TCT GAA TTA CTG TTT GAC |
| gRNA | hIRAKM_CRISPR_1S | CAC CGA CCT GTA AAA GGT CAC CGA |
| gRNA & qPCR | hIRAKM_CRISPR_1A | AAA CTC GGT GAC CTT TTA CAG GTC |
| gRNA | hIRAKM_CRISPR_2S | CAC CGC ACA GAA AAA CAA GAC CAT |
| gRNA & qPCR | hIRAKM_CRISPR_2A | AAA CAT GGT CTT GTT TTT CTG TGC |
| gRNA | hTRAF6_CRISPR_1S | CAC CGA AGC AGT GCA AAC GCC ATG |
| gRNA & qPCR | hTRAF6_CRISPR_1A | AAA CCA TGG CGT TTG CAC TGC TTC |
| gRNA | hTRAF6_CRISPR_2S | CAC CGC AGT CTA GAT CAT AAT GAT T |
| gRNA & qPCR | hTRAF6_CRISPR_2A | AAA CAA TCA TTA TGA TCT AGA CTG C |
| gRNA | hNEMO_CRISPR_1S | CAC CGA GCG CCC TGT TCT GAA GGC |
| gRNA & qPCR | hNEMO_CRISPR_1A | AAA CGC CTT CAG AAC AGG GCG CTC |
| gRNA | hNEMO_CRISPR_2S | CAC CGT CTG CTG CCG GGC CAC CAC T |
| gRNA & qPCR | hNEMO_CRISPR_2A | AAA CAG TGG TGG CCC GGC AGC AGA C |
| gRNA | hSTAMBP_CRISPR_1S | CAC CGA GCG GAA GTA CCG ACG GGG |
| gRNA & qPCR | hSTAMBP_CRISPR_1A | AAA CCC CCG TCG GTA CTT CCG CTC |
| gRNA | hSTAMBP_CRISPR_2S | CAC CGC CCT CAC CCG GTC TTC GGG |
| gRNA & qPCR | hSTAMBP_CRISPR_2A | AAA CCC CGA AGA CCG GGT GAG GGC |
| gRNA | hIKKalpha_CRISPR_1S | CAC CGC AAT GGA ATA CTG TTC TGG |
| gRNA | hIKKalpha_CRISPR_1A | AAA CCC AGA ACA GTA TTC CAT TGC |
| gRNA | hIKKbeta_CRISPR_1S | CAC CGA TTT GGA AAT GTC ATC CGA |
| gRNA | hIKKbeta_CRISPR_1A | AAA CTC GGA TGA CAT TTC CAA ATC |
| gRNA | hIKKbeta_CRISPR_2S | CAC CGT TGT TAG CGA AGA CTT GAA |
| gRNA | hIKKbeta_CRISPR_2A | AAA CTT CAA GTC TTC GCT AAC AAC |
| RT-qPCR | hNLRP7_qPCR_117_S | CGA AGA CGT GCT ACA GAA GAC |
| RT-qPCR | hNLRP7_qPCR_117_A | TTC ACA GTC GCA TTC CTT ATC C |
| RT-qPCR | hNLRP3_qPCR_81_S | GAT CTT CGC TGC GAT CAA CAG |
| RT-qPCR | hNLRP3_qPCR_81_A | CGT GCA TTA TCT GAA CCC CAC |
| RT-qPCR | hGAPDHS2 | TGT TGC CAT CAA TGA CCC CTT |
| RT-qPCR | hGAPDHA2 | CTC CAC GAC GTA CTC AGC G |
| PCR amplicon nanopore sequencing | hNLRP7_exon2_S | CCC AGG TTC AAG TGT GAT TCT CC |
| PCR amplicon nanopore sequencing | hNLRP7_exon4_A | CTC CTT CTC CAG GGC GTA GAA C |
| Vector construction | hNLRP7_pCWTet_S | TGG AGA ATT GGC TAG GCC ACC ATG ACA TCG CCC CAG CTA GAG TGG AC |
| Vector construction | hNLRP7_pCWTet_A | AAC CCC AAC CCC TTA TCA GCA AAA AAA GTC ACA GCA CGG AGG TG |
| Vector construction | HpaBFPNhe_pCWTet_S | TGG AGA ATT GGC TAG CCA CCA TGG TTA ACA CGC GTG TGA GCA AGG GCG AGG AGC TGT |
| Vector construction | HpaBFPNhe_pCWTet_A | AAC CCC AAC CCC TTA TCA GCT AGC CTT GTA CAG CTC GTC CAT GCC GAG |
| Vector construction | hSLC7A11_pCWTet_S | TGG AGA ATT GGC TAG GCC ACC ATG GTC AGA AAG CCT GTT GTG TCC AC |
| Vector construction | hSLC7A11_pCWTet_A | AAC CCC AAC CCC TTA TTA TAA CTT ATC TTC TTC TGG TAC AAC |
| Vector sequencing | pCW_TREpro_S_seq | GAG AAC GTA TGT CGA GGT AGG |
| Vector sequencing | pCW_PKGpro_A_seq | TGC GAA CGG ACG TGA AGA ATG TG |
| Vector sequencing | hNLRP7_mid_seq_1S | TGA TCT CCA AAG ACT GGC CTG |
| Vector sequencing | hNLRP7_mid_seq_2S | ATC CAC CTC AGC TTC CAG CAG |
| Vector sequencing | hNLRP7_mid_seq_3S | TGG GAA CGC ACG ATG ATG CTG |
| Vector sequencing | hNLRP7_seq_A1 | AGA AGA ATT CAG CCC ACT GCT CC |
| CRISPR/Cas9-based screen | LentiGuide_gRNAs6 | TTGGAATCACACGACCTGGATGGA |
| CRISPR/Cas9-based screen | LentiGuide_gRNAa1_3 | TCTCTGCTGTCCCTGTAATAAACCCG |
| CRISPR/Cas9-based screen (qPCR) | LentiGuide_gRNAs2 | GACTATCATATGCTTACCGTAAC |
| CRISPR/Cas9-based screen (qPCR) | LentiGuide_gRNAs4 | AGCTTGGCGTAACTAGATCTTGAG |
| CRISPR/Cas9-based screen (qPCR) | LentiGuide_gRNAa3 | ACTATTCTTTCCCCTGCACTGT |
